# Evaluation of clinical value and potential mechanism of MTFR2 in lung adenocarcinoma via bioinformatics

**DOI:** 10.1186/s12885-021-08378-3

**Published:** 2021-05-26

**Authors:** Cheng Chen, Yang Tang, Wen-Dong Qu, Xu Han, Jie-Bin Zuo, Qing-Yong Cai, Gang Xu, Yong-Xiang Song, Xi-Xian Ke

**Affiliations:** Department of Thoracic Surgery, Affiliated Hospital of Zunyi Medical University, Zunyi, 563000 Guizhou China

**Keywords:** Lung adenocarcinoma, Prognosis, MTFR2, Bioinformatics, Biomarker

## Abstract

**Background:**

Mitochondrial fission regulator 2 (MTFR2) was involved in the progression and development of various cancers. However, the relationship between MTFR2 with lung adenocarcinoma (LUAD) had not been reported. Herein, this study analyzed the clinical significance and potential mechanisms of MTFR2 in LUAD via bioinformatics tools.

**Results:**

We found that the level of MTFR2 was increased, and correlated with sex, age, smoking history, neoplasm staging, histological subtype and TP53 mutation status in LUAD patients. Kaplan-Meier survival analysis showed LUAD patients with increased MTFR2 had a poor prognosis. In addition, univariate COX regression analysis showed neoplasm staging, T stage, distant metastasis and MTFR2 level were risk factors for the prognosis of LUAD. A total of 1127 genes were coexpressed with MTFR2, including 840 positive and 208 negative related genes. KEGG and GSEA found that MTFR2 participated in the progression of LUAD by affecting cell cycle, DNA replication, homologous recombination, p53 signaling pathway and other mechanisms. The top 10 coexpressed genes, namely CDK1, CDC20, CCNB1, PLK1, CCNA2, AURKB, CCNB2, BUB1B, MAD2L1 and BUB1 were highly expressed, and were associated with poor prognosis in LUAD.

**Conclusions:**

Consequently, we elucidated MTFR2 was a biomarker for diagnosis and poor prognosis in LUAD, and might participate in the progression of LUAD via affecting cell cycle, DNA replication, homologous recombination and p53 signaling pathway.

**Supplementary Information:**

The online version contains supplementary material available at 10.1186/s12885-021-08378-3.

## Introduction

Non-small cell lung cancer (NSCLC) was one of the most common malignant tumors in the world, and lung adenocarcinoma (LUAD) was one of the common subtype of NSCLC [1, 2]. LUAD patients in early stage got improved long-term prognosis by surgery and neoadjuvant chemotherapy, while due to incomplete excision, primary and secondary drug resistance and other reasons, recurrence and metastasis happened. The 5-year survival rate of the patients was still very low, and its treatment was extremely challenging [2, 3]. In recent years, targeted therapy had greatly improved the prognosis of patients with LUAD, but the mortality of patients with LUAD remained at a high level [4–6]. Therefore, it was of great significance to further discover the novel molecules and explore their regulatory mechanisms involved in the occurrence and development of LUAD resulting in finding novel therapeutic targets.

Mitochondrial fission regulator 2 (MTFR2), also known as FAM54A, was located on the 6q23.2 chromosome and belonged to the MTFR family. Abnormal mitosis of mitochondria was related to the pathogenesis of many diseases, as well as occurrence and prognosis of several tumors [7–11]. MTFR2 was up-regulated in breast cancer and was related to age, lymph node metastasis, and prognosis in HER2 positive breast cancer patients. Knockout of MTFR2 prohibited the proliferation, migration and invasion of breast cancer cells, and blocked epithelial-stromal transformation (EMT) [8]. MTFR2 affected the transcription of TTK by activating the transcriptional promoter. MTFR2-dependent TTK regulation played a key role in maintaining GSC (Glioma stem-like cells) in glioma, and may be a target molecule of new drugs for glioma patients [12]. Whereas, at present, rare researches were focused on MTFR2 in other tumors including LUAD, except for those cancers listed above. Herein, the present study explored the clinical value and potential regulatory mechanism of MTFR2 in the progression of LUAD through multiple databases.

## Results

### MTFR2 was upregulated and correlated with sex, age, smoking history, cancer stage, histological subtype and TP53 mutation status in LUAD patients

**Fig. 1:**
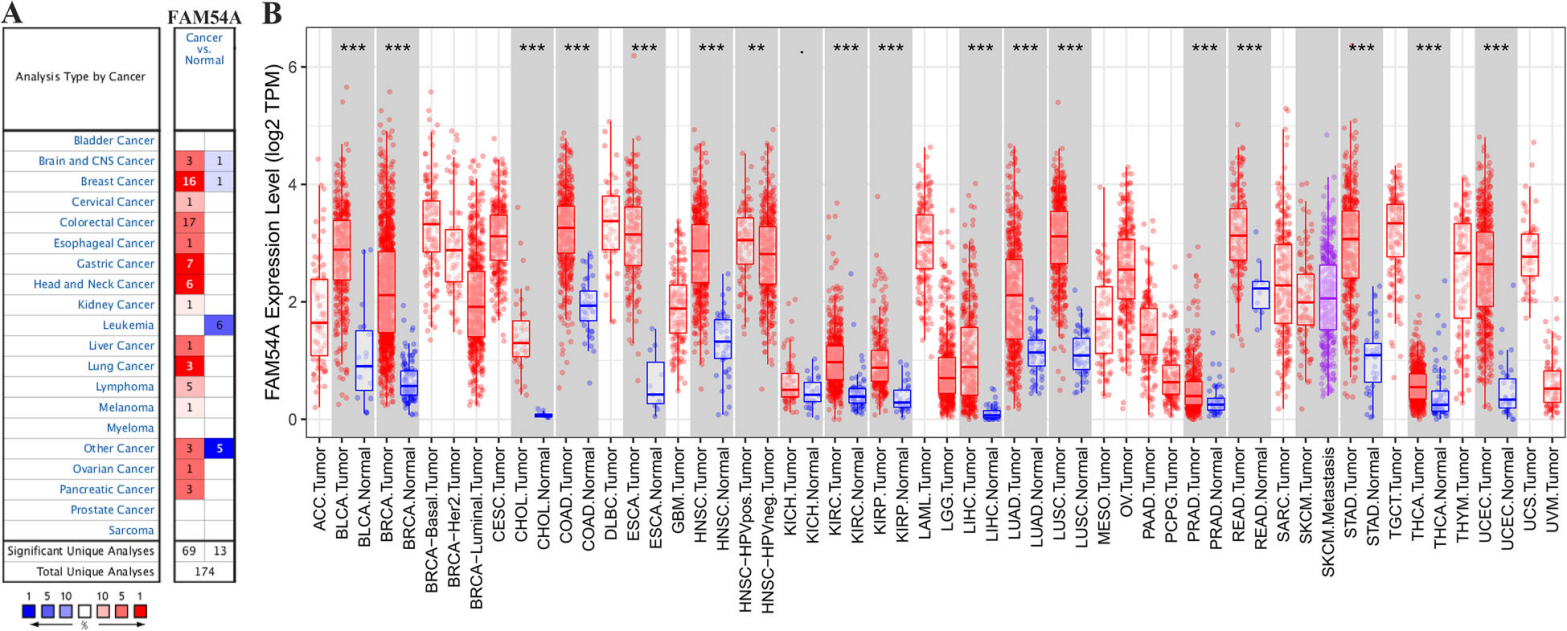
MTFR2 expressions in cancer tissues. **A** Oncomine database; **B** Timer database. Note: **A** Red, increased expression of MTFR2; blue, decreased expression of MTFR2; **B** red, cancer tissues; blue, normal tissues. *, *p* < 0.05; **, *p* < 0.01; ***, *p* < 0.001

MTFR2 was abnormally expressed in pan-cancerous tissues, and was mainly over-expressed in a variety of tumor tissues (Fig. 1). In detail, in the Oncomine database, MTFR2 was highly expressed in breast cancer, colorectal cancer, gastric cancer, lung cancer and other tumor tissues, while lowly- expressed in brain nerve tumor, breast cancer, leukemia and other tumor tissues (Fig. 1A). In the Timer database, MTFR2 was highly expressed in BLCA, BRCA, CHOL, LUSC, LUAD and other tumor tissues (Fig. 1B).

**Fig. 2:**
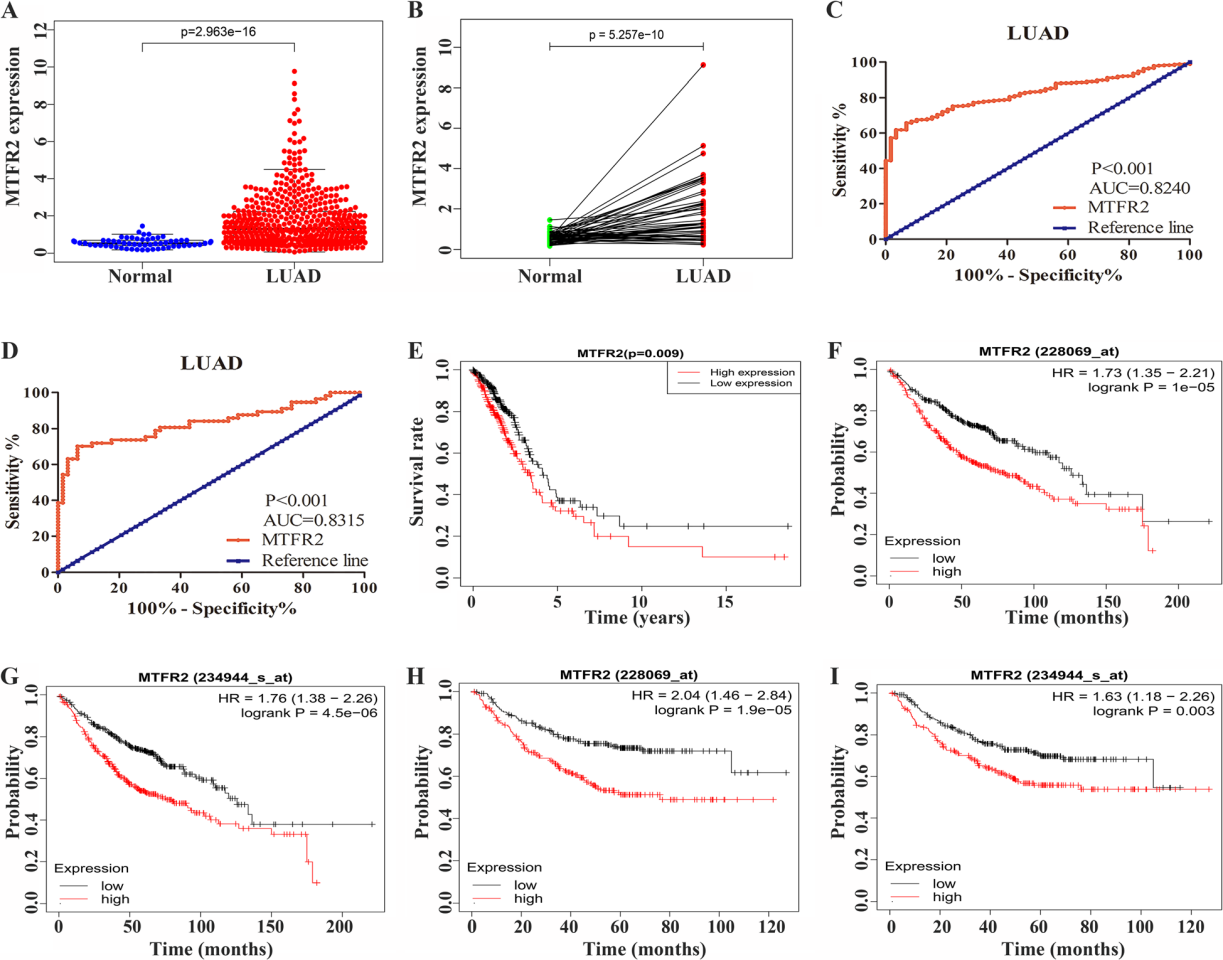
The expression of MTFR2 was correlated to diagnosis and prognosis in LUAD patients. **A** Expression of MTFR2 in unpaired LUAD tissues; **B** Expression of MTFR2 in 57 paired LUAD tissues; **C** The diagnostic value of MTFR2 in unpaired LUAD tissues; **D** The diagnostic value of MTFR2 in 57 paired LUAD tissues; **E** The expression of MTFR2 in TCGA database was correlated with the OS of LUAD patients; **F**-**G** The levels of MTFR2 (228069_at) and MTFR2 (234944_at) were correlated to the OS of LUAD patients with Kaplan-Meier Plotter analysis; **H**-**I** The levels of MTFR2 (228069_at) and MTFR2 (234944_at) were correlated to the early progression of LUAD patients with Kaplan-Meier Plotter analysis. Note: Normal, normal lung tissues; LUAD, lung adenocarcinoma tissues; AUC, Area under curve

In addition, we found consistent results in the Ualcan database, where MTFR2 was highly expressed in LUAD tissues (Table 1). In addition, in the TCGA database, the expression of MTFR2 was increased in unpaired LUAD patients (Fig. 2A) and in 57 pairs of LUAD patients (Fig. 2B).

**Table 1:** The expression of MTFR2 in Ualcan database was correlated to the clinicopathological characteristics in LUAD patients

Clinicopathological characteristics	*p* value
Expression	
Normal-vs-Primary	1.62447832963153E-12
Gender	
Male-vs-Female	4.185800E-02
Age	
Age(41-60Yrs)-vs-Age(61-80Yrs)	1.089890E-02
Smoking	
Non smoker-vs-Smoker	7.3849000004067E-07
Non smoker-vs-Reformed smoker2	6.560800E-03
Smoker-vs-Reformed smoker1	1.53600000496468E-08
Smoker-vs-Reformed smoker2	1.978210E-03
Reformed smoker1-vs-Reformed smoker2	1.955780E-04
Cancer stages	
Stage1-vs-Stage2	4.427500E-02
Tissue types	
NOS-vs-Mixed	2.900200E-02
NOS-vs-LBC-Non Mucinous	2.038200E-03
NOS-vs-Solid Pattern Predominant	2.599600E-02
NOS-vs-Acinar	5.378900E-03
NOS-vs-LBC-Mucinous	1.70860000003881E-06
NOS-vs-Papillary	1.027880E-04
Mixed-vs-Solid Pattern Predominant	1.196000E-02
Mixed-vs-LBC-Mucinous	4.00150000001709E-06
Mixed-vs-Papillary	4.149500E-02
Clear Cell-vs-LBC-Non Mucinous	4.028200E-02
Clear Cell-vs-Papillary	2.063500E-02
LBC-Non Mucinous-vs-Solid Pattern Predominant	4.452100E-02
LBC-Non Mucinous-vs-LBC-Mucinous	1.174210E-02
Solid Pattern Predominant-vs-LBC-Mucinous	1.926450E-02
Solid Pattern Predominant-vs-Mucinous carcinoma	4.480600E-03
Solid Pattern Predominant-vs-Papillary	3.801800E-02
Acinar-vs-LBC-Mucinous	1.693590E-02
LBC-Mucinous-vs-Papillary	1.804030E-02
TP53 Mutant	
TP53-Mutant-vs-TP53-Non Mutant	1.81166193158333E-12

Note: Reformed smoker 1, Reformed smoker (< 15 years); Reformed smoker 2, Reformed smoker (> 15 years); NOS, Lung Adenocarcinoma-Not Otherwise Specified; Mixed, Lung Adenocarcinoma Mixed subtype; Clear Cell, Lung Clear Cell Adenocarcinoma; LBC-Nonmucinous, Lung Bronchioloalveolar Carcinoma Non mucinous; Solid Pattern Predominant, Lung Solid Pattern Predominant Adenocarcinoma; Acinar, Lung Acinar Adenocarcinoma; LBC-Mucinous, Lung Bronchioloalveolar Carcinoma Mucinous; Mucinous, Mucinous (Colloid) Carcinoma; Papillary, Lung Papillary Adenocarcinoma; Mucinous, Lung Mucinous Adenocarcinoma; Micropapillary, Lung Micropapillary Adenocarcinoma; Signet Ring, Lung Signet Ring Adenocarcinoma

In the Ualcan database, we found that the expression of MTFR2 was related to the clinicopathological features of LUAD patients (Table 1). In detail, the level of MTFR2 was related to the sex (Male vs Female), Age (Age(41-60Yrs) vs Age (61-80Yrs)), Smoking (Non smoker vs Smoker; Non smoker vs Reformed smoker2; Smoker vs Reformed smoker1; Smoker vs Reformed smoker2; Reformed smoker1 vs Reformed smoker2), histological subtype (NOS vs Mixed; NOS vs LBC-Non Mucinous; NOS vs Solid Pattern Predominant; NOS vs Acinar; NOS vs LBC-Mucinous; NOS vs Papillary; Mixed vs Solid Pattern Predominant; Mixed vs LBC-Mucinous; Mixed vs Papillary; Clear Cell vs LBC-Non Mucinous; Clear Cell vs Papillary; LBC-Non Mucinous vs Solid Pattern Predominant; LBC-Non Mucinous vs LBC-Mucinous; Solid Pattern Predominant vs LBC-Mucinous; Solid Pattern Predominant vs Mucinous carcinoma; Solid Pattern Predominant vs Papillary; Acinar vs LBC-Mucinous; LBC-Mucinous vs Papillary) and TP53 mutation (TP53-Mutant vs TP53-Non Mutant) of LUAD patients.

In TCGA database, the area under the curve (AUC) showed that the level of MTFR2 had a diagnostic value in LUAD patients via the ROC analysis (Fig. 2C and D). Further Kaplan-Meier analysis showed that the LUAD patients with increased MTFR2 had a poor prognosis (Fig. 2E). In addition, in the Kaplan-Meier Plotter database, the levels of MTFR2 (228069_at) and MTFR2 (234944_at) were associated with the overall survival and early disease progression of LUAD patients (Fig. 2F-I). Univariate Cox analysis of complete clinical data of LUAD patients in TCGA database showed that cancer stage, T stage, lymphatic metastasis as well as MTFR2 expression were risk factors for the prognosis of LUAD patients (Table 2). Further multivariate Cox analysis showed that cancer stage was an independent risk factor for the prognosis of LUAD patients (Table 2).

**Table 2:** COX regression analysis of the correlation between clinicopathological features with prognosis

Clinical characteristics	HR	HR 95% CI low	HR 95% CI up	*p* value
a				
Age	1.057406203	0.713393673	1.567308372	0.781014797
Gender	1.00097432	0.698837323	1.433737948	0.995761563
Stage	1.64465101	1.396688	1.93663649	2.42E-09
T	1.623091548	1.309819761	2.011289072	9.57E-06
M	1.681168333	0.923680619	3.059853055	0.08910352
N	1.792676516	1.464854278	2.193862653	1.47E-08
MTFR2	1.096892408	1.002541345	1.200123029	0.043876089
b				
Stage	1.37421707	1.094758223	1.725013356	0.006135636
T	1.223211397	0.971744105	1.539753227	0.086187124
N	1.283860776	0.991173421	1.662976889	0.05838382
MTFR2	1.062548364	0.964968929	1.169995211	0.217045634

### Screening of MTFR2 co-expressed genes

**Fig. 3:**
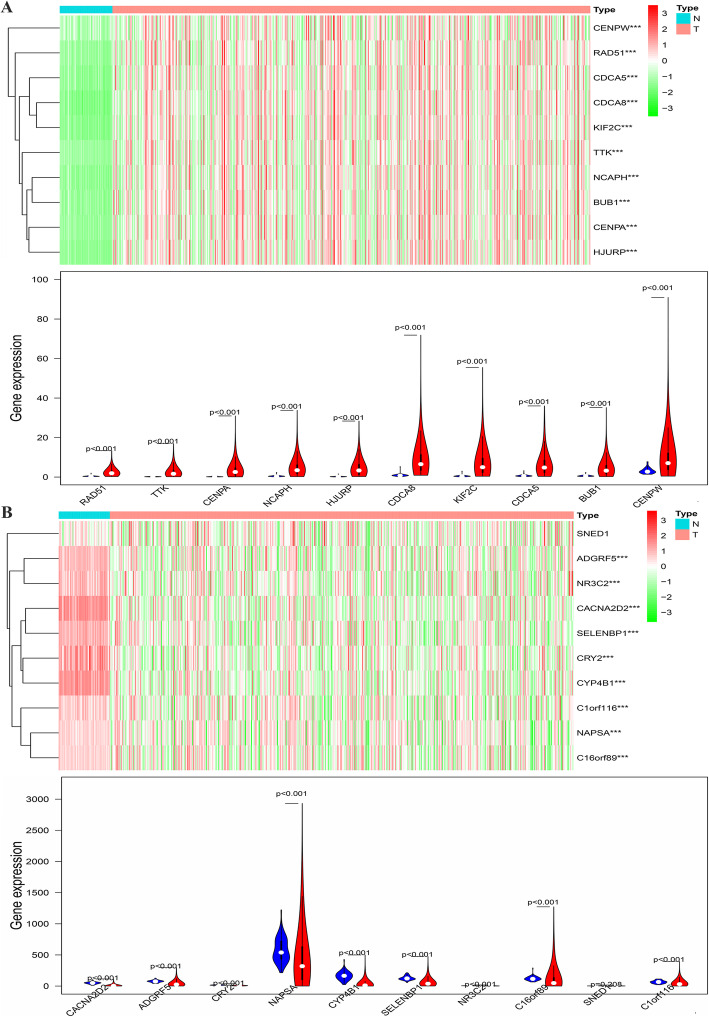
The expressions of the top 10 positive and negative related genes of MTFR2 in LUAD tissues. **A** The top 10 positively correlated genes; **B** The top 10 negatively correlated genes. Note: ***, *p* < 0.001; N, normal lung tissue; T, lung adenocarcinoma tissue

We screened 1127 MTFR2 co-expressed genes in TCGA database by screening conditions, most of which were positive related genes. Among them, there were 840 positive and 208 negative related genes (S1 Table). The top 10 genes that were positively and negatively related to MTFR2 (Table 3). The expression of the top 10 positive and negative related genes of MTFR2 in LUAD tissues from TCGA were abnormally expressed (Fig. 3).

**Table 3:** The top 10 genes with positive and negative correlation to MTFR2

Gene	Corgene	Cor	*p* value
MTFR2	CENPW	0.876	1.08E-170
MTFR2	TTK	0.849	7.63E-150
MTFR2	NCAPH	0.848	4.00E-149
MTFR2	RAD51	0.835	1.43E-140
MTFR2	CENPA	0.827	2.59E-135
MTFR2	KIF2C	0.817	2.83E-129
MTFR2	BUB1	0.817	1.98E-129
MTFR2	CDCA8	0.816	5.28E-129
MTFR2	HJURP	0.814	7.81E-128
MTFR2	CDCA5	0.814	1.26E-127
MTFR2	C16orf89	−0.655	7.35E-67
MTFR2	CRY2	−0.628	3.92E-60
MTFR2	C1orf116	−0.585	1.60E-50
MTFR2	CYP4B1	−0.583	5.72E-50
MTFR2	SELENBP1	−0.58	2.16E-49
MTFR2	CACNA2D2	−0.576	1.56E-48
MTFR2	ADGRF5	−0.568	4.68E-47
MTFR2	NAPSA	−0.561	1.09E-45
MTFR2	SNED1	−0.558	4.89E-45
MTFR2	NR3C2	−0.557	6.44E-45

Note: Corgene, co-expressed gene; Cor, correlation coefficient

### Analysis of MTFR2 function and its coexpressed genes by GO, KEGG and GSEA

**Fig. 4:**
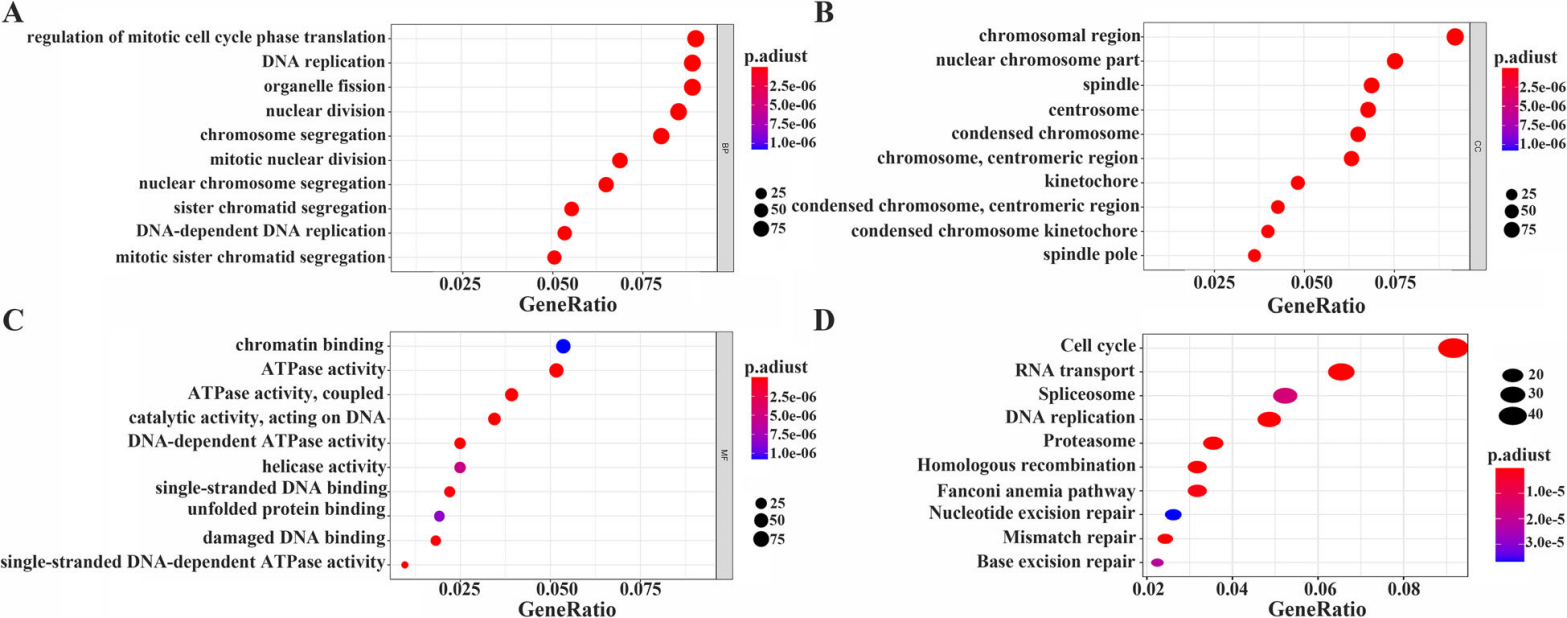
Analysis of MTFR2 co-expressed genes by GO and KEGG. **A** BP; **B** CC; **C** MF; **D** KEGG

**Fig. 5:**
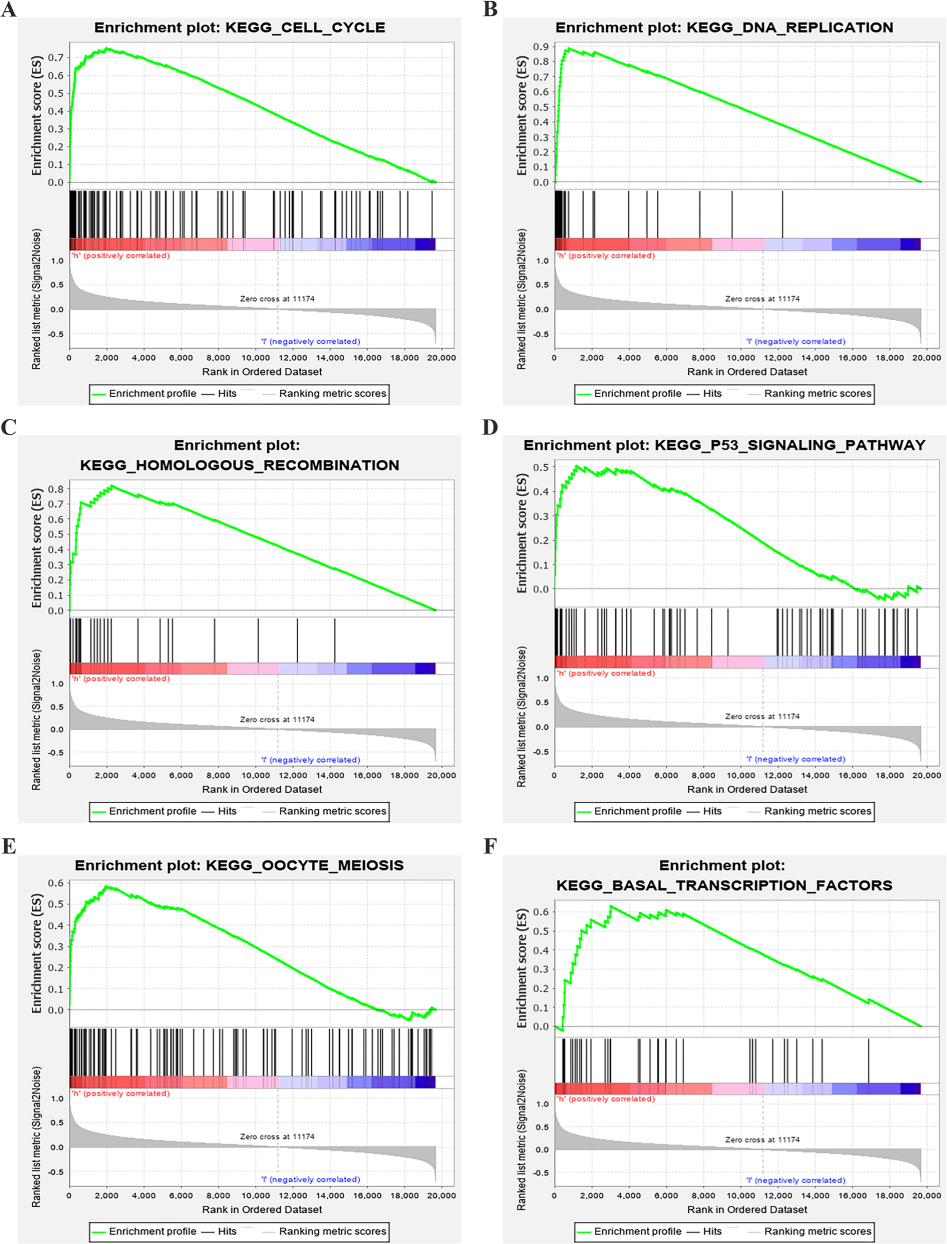
GSEA displayed the main signal pathways derived from high expression of MTFR2 enrichment. **A** Cell cycle; **B** DNA replication; **C** Homologous recombination; **D** p53 signaling pathway; **E** Oocyte meiosis; **F** Base_excision_ repair

In order to further understand the potential function of MTFR2 in the development of LUAD, we carried out GO and KEGG analysis of MTFR2 co-expressed genes. MTFR2 coexpressed genes were involved in BP, CC and MF (Fig. 4A-C), and in details, were mainly involved in DNA replication, chromosome segregation, mitotic nuclear division, mitotic cell cycle phase transition and other processes (S2 Table). KEGG pathway analysis indicated MTFR2 co-expressed genes were mainly involved in cell cycle, DNA replication, proteasome, homologous recombination, spliceosome, nucleotide excision repair, human T-cell leukemia virus 1 infection, p53 signaling pathway and other signal pathways (Fig. 4D and Table 4). What’s more, in GSEA database, we found that cell cycle, DNA_replication, homologous recombination, p53 signal pathway, oocyte meiosis and base_excision repair signaling pathways were highly enriched in MTFR2 highly expressed group (Fig. 5 and Table 5). To sum up, these results suggested that MTFR2 might regulate the progress of LUAD through cell cycle, DNA replication, homologous recombination and p53 signaling pathway.

**Table 4:** MTFR2 co-expressed genes by KEGG

ID	Description	Count	*p* value	*p* adjust
hsa04110	Cell cycle	49	2.54E-26	7.53E-24
hsa03030	DNA replication	26	1.92E-23	2.84E-21
hsa03050	Proteasome	19	2.45E-11	2.42E-09
hsa03440	Homologous recombination	17	2.60E-10	1.59E-08
hsa03430	Mismatch repair	13	2.69E-10	1.59E-08
hsa03013	RNA transport	35	5.88E-09	2.90E-07
hsa03460	Fanconi anemia pathway	17	3.58E-08	1.52E-06
hsa03040	Spliceosome	28	4.24E-07	1.57E-05
hsa03410	Base excision repair	12	6.52E-07	2.14E-05
hsa03420	Nucleotide excision repair	14	1.25E-06	3.69E-05
hsa04114	Oocyte meiosis	24	2.97E-06	8.00E-05
hsa00670	One carbon pool by folate	8	2.25E-05	0.000555487
hsa03008	Ribosome biogenesis in eukaryotes	20	3.63E-05	0.000826201
hsa04218	Cellular senescence	24	0.000136465	0.002885268
hsa04914	Progesterone-mediated oocyte maturation	17	0.000252864	0.004989844
hsa00240	Pyrimidine metabolism	12	0.000297856	0.00551034
hsa05012	Parkinson disease	31	0.000505075	0.008794241
hsa05166	Human T-cell leukemia virus 1 infection	28	0.000610833	0.010044803
hsa00270	Cysteine and methionine metabolism	10	0.001213762	0.018909138
hsa04115	p53 signaling pathway	12	0.002598093	0.038451783

**Table 5:** GSEA displayed the main signal pathways derived from high expression of MTFR2 enrichment

Name	Size	Nes	Nom *p* value
KEGG_CELL_CYCLE	124	2.5325148	0
KEGG_SPLICEOSOME	126	2.342574	0
KEGG_OOCYTE_MEIOSIS	112	2.3013418	0
KEGG_MISMATCH_REPAIR	23	2.2582684	0
KEGG_DNA_REPLICATION	36	2.210251	0
KEGG_HOMOLOGOUS_RECOMBINATION	28	2.207216	0
KEGG_RNA_DEGRADATION	57	2.2033587	0
KEGG_PROGESTERONE_MEDIATED_OOCYTE_MATURATION	85	2.1570013	0
KEGG_PROTEASOME	44	2.108183	0
KEGG_BASE_EXCISION_REPAIR	33	2.097559	0
KEGG_PATHOGENIC_ESCHERICHIA_COLI_INFECTION	55	2.096588	0
KEGG_GLYOXYLATE_AND_DICARBOXYLATE_METABOLISM	16	2.0935147	0
KEGG_BASAL_TRANSCRIPTION_FACTORS	35	2.051244	0
KEGG_PYRIMIDINE_METABOLISM	98	2.0281448	0
KEGG_ONE_CARBON_POOL_BY_FOLATE	17	2.0059762	0
KEGG_P53_SIGNALING_PATHWAY	68	1.96642	0
KEGG_PURINE_METABOLISM	157	1.9532791	0
KEGG_NUCLEOTIDE_EXCISION_REPAIR	44	2.0500638	0.001831502
KEGG_UBIQUITIN_MEDIATED_PROTEOLYSIS	133	1.7833992	0.003868472
KEGG_CYSTEINE_AND_METHIONINE_METABOLISM	34	1.8469852	0.003883495
KEGG_FRUCTOSE_AND_MANNOSE_METABOLISM	33	1.821762	0.011516315
KEGG_PENTOSE_PHOSPHATE_PATHWAY	27	1.7594053	0.011650485
KEGG_N_GLYCAN_BIOSYNTHESIS	46	1.6938915	0.023346303
KEGG_BLADDER_CANCER	42	1.5152026	0.0251938
KEGG_RIBOFLAVIN_METABOLISM	16	1.5648233	0.027675277
KEGG_PANCREATIC_CANCER	70	1.4912151	0.03131524
KEGG_GLYCOLYSIS_GLUCONEOGENESIS	62	1.5601822	0.036398467
KEGG_HUNTINGTONS_DISEASE	177	1.6180334	0.046511628
KEGG_RNA_POLYMERASE	29	1.5426548	0.048732944

### Construction of PPI network and the expressions of hub genes and their prognostic value

**Fig. 6:**
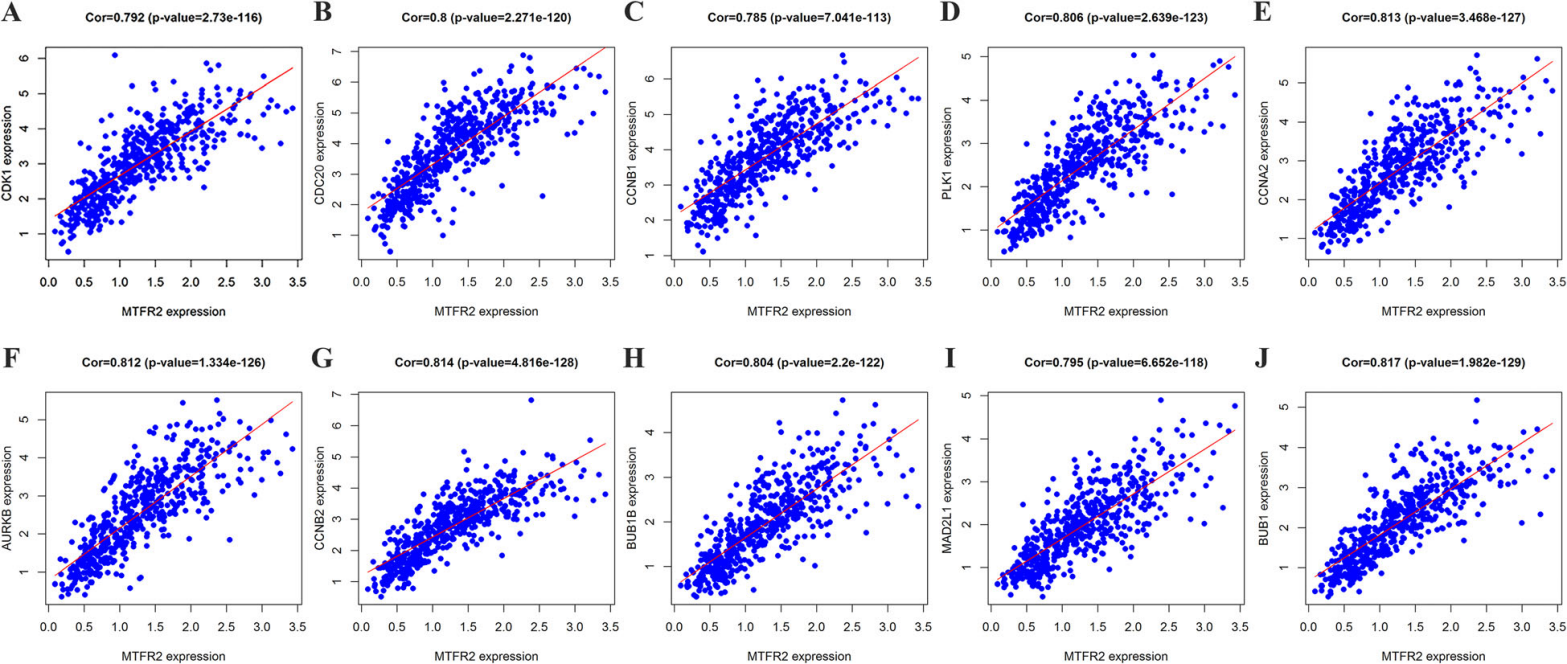
The expression of MTFR2 was correlated to the top 10 Hub genes. **A** CDK1; **B** CDC20; **C** CCNB1; **D** PLK1; **E** CCNA2; **F** AURKB; **G** CCNB2; **H** BUB1B; **I** MAD2L1; **J** BUB1

**Fig. 7:**
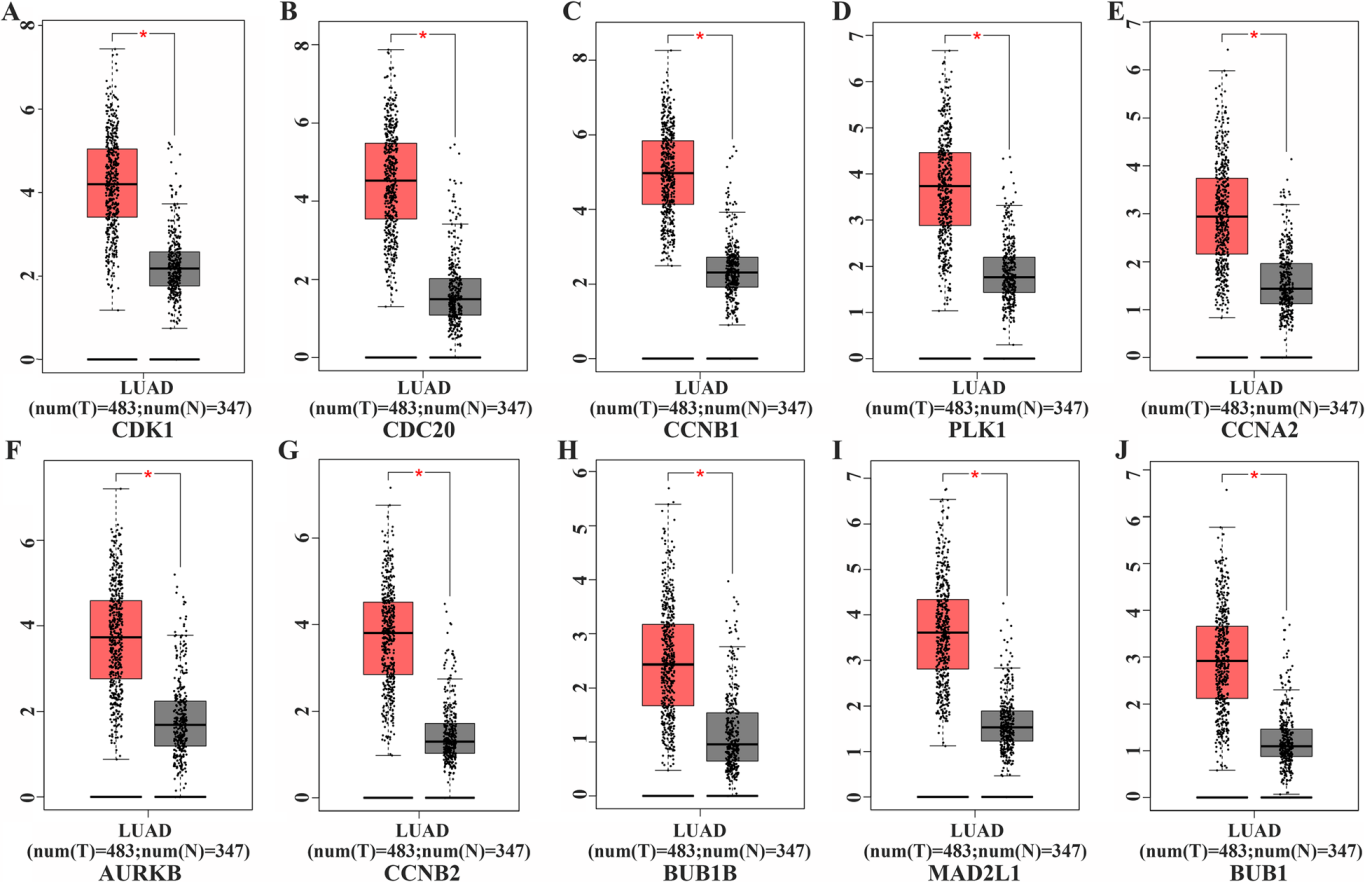
The expressions of the top10 Hub genes in LUAD tissues from TCGA Database. **A** CDK1; **B** CDC20; **C** CCNB1; **D** PLK1; **E** CCNA2; **F** AURKB; **G** CCNB2; **H** BUB1B; **I** MAD2L1; (J) BUB1. Note: T, LUAD tissues; N, normal tissues; red, LUAD tissues; gray, normal lung tissues; *, *p* < 0.05

**Fig. 8:**
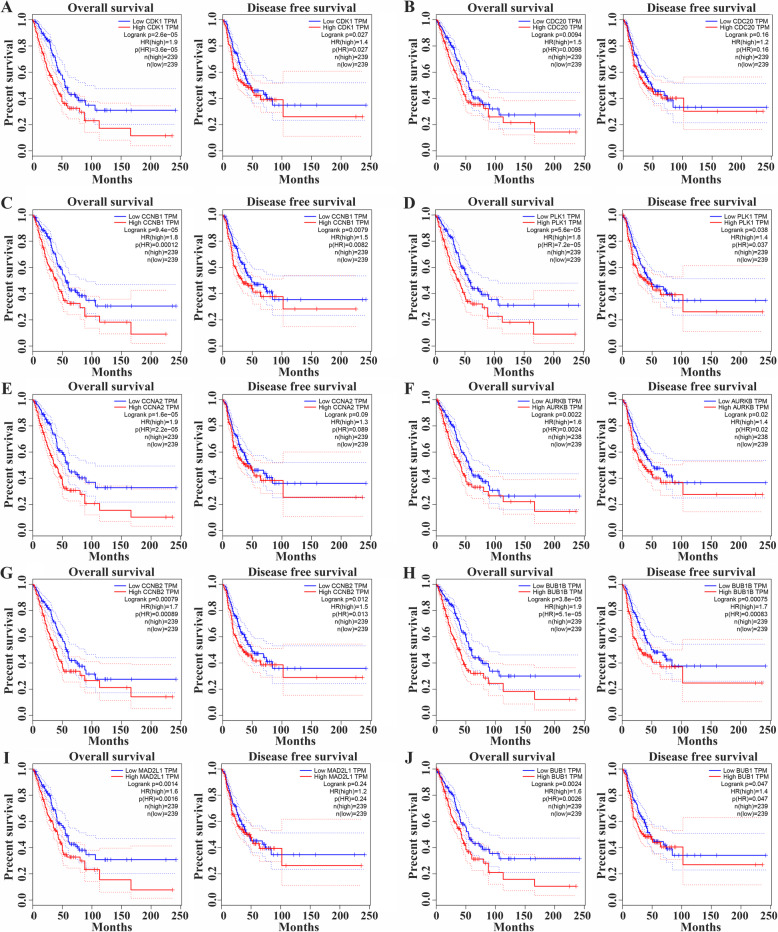
The prognosis values of Hub genes in LUAD from GEPIA. **A** CDK1; **B** CDC20; **C** CCNB1; **D** PLK1; **E** CCNA2; **F** AURKB; **G** CCNB2; **H** BUB1B; **I** MAD2L1; **J** BUB1

By identifying the function of MTFR2 co-expressed genes, the potential biological function of MTFR2 was also inferred. The top 10 Hub genes in the PPI network were selected by the CytoHubba plug-in as CDK1, CDC20, CCNB1, PLK1, CCNA2, AURKB, CCNB2, BUB1B, MAD2L1 and BUB1 (S1 Fig and Table 6). The expression of MTFR2 was correlated with that of CDK1, CDC20, CCNB1, PLK1, CCNA2, AURKB, CCNB2, BUB1B, MAD2L1 and BUB1 (Fig. 6). In addition, GEPIA database was used to analyze the expressions of Hub genes and their prognostic values. We found that the expressions of CDK1, CDC20, CCNB1, PLK1, CCNA2, AURKB, CCNB2, BUB1B, MAD2L1 and BUB1 genes were significantly increased in LUAD tissues (Fig. 7). The top10 Hub genes were associated with OS of LUAD patients, and CDK1, CCNB1, PLK1, AURKB, CCNB2, BUB1B and BUB1 were associated with DFS in LUAD patients with disease-free progression (Fig. 8).

**Table 6:** The top 10 Hub genes in PPI Network

Name	Description	Score
CDK1	Cyclin dependent kinase 1	192
CDC20	Cell division cycle 20	160
CCNB1	Cyclin B1	138
PLK1	Polo like kinase 1	134
CCNA2	Cyclin A2	129
AURKB	Aurora kinase B	127
CCNB2	Cyclin B2	124
BUB1B	BUB1 mitotic checkpoint serine/threonine kinase B	118
MAD2L1	Mitotic arrest deficient 2 like 1	117
BUB1	BUB1 mitotic checkpoint serine/threonine kinase	105

## Discussion

Mitochondria, via regulating the membrane potential, participated in programmed cell death as well as cell proliferation [13, 14]. MTFR2 played an vital role in promoting the division of mitochondria in cells. Under pathological condition, abnormal mitosis of mitochondria was related to the development of tumors [10, 11]. At present, studies found that the expression of MTFR2 in breast cancer tissues were increased, and was correlated to the clinicopathological features and poor prognosis of patients. Interfering with MTFR2 inhibited the growth and migration of breast cancer cells, indicating its biological role as oncogene and with prognostic value [8]. However, the role of MTFR2 in LUAD was still not been elucidated. In present study, it was found for the first time that MTFR2 was highly expressed in LUAD tissues in multiple databases, and the level of MTFR2 was related to sex, age, smoking history, cancer stage, histological subtypes and TP53 mutation status in LUAD patients. ROC analysis showed that the expression of MTFR2 had diagnostic value in LUAD. Kaplan-Meier analysis showed that the overall survival time of LUAD patients with increased MTFR2 expression was related to the early disease progression. Univariate Cox analysis showed that cancer stage, T stage, distant metastasis and the level of MTFR2 were risk factors for the prognosis in LUAD. These results indicated that MTFR2 played an important role in the development of LUAD.

Abnormal cell cycle leaded to abnormal regulation of cell growth and induced overgrowth of cells, resulting in poor prognosis of tumor patients. Therefore, through a detailed understanding of the mechanism of tumor growth and inhibition of tumor occurrence and development, the prognosis of patients could be improved [15–17]. Nichols et al. reported that high mutation burden in LUAD patients with increased HORMAD1 expression decreased the survival rate of LUAD patients. HORMAD1 was one of the key genes in the process of homologous recombination (HR), and promoted the formation of RAD51 filaments to participate in the process of homologous recombination. Interfering with HORMAD1 reduced the growth of tumor cells [18]. FAM111B was highly expressed in LUAD tissues. LUAD patients with high expression of FAD111B had low relapse-free survival (RFS) and overall survival (OS). Interfering with FAM111B inhibited the proliferation, cell cycle arrest and migration of LUAD cells and the ability of tumor formation in nude mice, but induced apoptosis. FAM111B might regulate the growth of LUAD through p53 signaling pathway, inhibited the expression of BAG3 and BCL2, and played an important role in cell cycle and apoptosis [19]. In present study, we found that MTFR2 coexpressed genes were mainly involved in cell cycle, DNA replication, homologous recombination, p53 signaling pathway and other signaling pathways by KEGG. Secondly, in GSEA, we also found that cell cycle, DNA replication, homologous recombination and p53 signaling pathways were highly enriched in MTFR2 highly-expressed group. Up to now, it was found that interfering with MTFR2 affected the process of EMT [8], but the role of MTFR2 in LUAD was still not been elucidated. However, MTFR2 promoted mitochondrial division in cells, which was related to tumor progression. Therefore, the interaction between MTFR2 with cell cycle, DNA replication, homologous recombination and p53 signaling pathway was worth in-depth study in LUAD.

Differential expression of genes in the process of tumorigenesis and development regulated the progression of cancers and affected the prognosis [20–23]. Polo-like kinase1 (PLK1) was a key regulatory factor in cell cycle and apoptosis. PLK1 was overexpressed in many kinds of Cutaneous T-cell lymphomas (CTCL) cells. Down-regulation of PLK1 inhibited cell growth, stability and proliferation, and also leaded to cell cycle arrest, mitotic protein changes, apoptosis and mitotic abnormalities [20]. CCNA2 was highly expressed, and was correlated to poor prognosis in NSCLC patients [22]. Topoisomerase IIA (TOP2A) was highly expressed, and was associated with poor prognosis in LUAD, indicating that TOP2A was a prognostic marker. Knockdown of TOP2A in A549 and GLC82 cells inhibited cell proliferation, migration and invasion, and decreased the expression of CCNB1 and CCNB2, indicating that it promoted the progress of LUAD by targeting CCNB1 and CCNB2 [23]. In this study, we found that the hub genes were involved in a variety of signal pathways including those in the occurrence and development of tumors. PPI network analyzed by GEPIA showed that the expressions of CDK1, CDC20, CCNB1, PLK1, CCNA2, AURKB, CCNB2, BUB1B, MAD2L1 and BUB1 were increased and related to the overall survival (OS), and CDK1, CCNB1, PLK1, AURKB, CCNB2, BUB1B and BUB1 were associated with disease-free survival (DFS) in LUAD patients. The expression of MTFR2 was also closely related to that of CDK1, CDC20, CCNB1, PLK1, CCNA2, AURKB, CCNB2, BUB1B, MAD2L1 and BUB1, which further indicated that MTFR2 and Hub genes played an vital role in the diagnosis, treatment and disease progression of LUAD.

Generally speaking, we found that the high level of MTFR2 was related to the clinicopathological features, diagnosis and prognosis, which might be used as a potential target for diagnosis and prognosis, and MTFR2 promoted the occurrence and development through cell cycle, DNA replication, homologous recombination and p53 signal pathway in LUAD.

## Materials and methods

### Oncomine and Timer database

The level of MTFR2 in pan-cancer tissues was analyzed by Oncomine database (https:// www.oncomine.org/resource/login) [24] and Timer database (https://cistrome.shinyapps.io/timer/) [25]. Screening criteria for Oncomine database: (1) Gene: FAM54A; (2) Analysis Type: Cancer vs Normal; (3) Date Type: mRNA; (4) *P* < 0.05; (5) Fold Chang ≥1.5. Search for FAM54A in the gene module of Timer database to analyze its level in pan-cancer tissues.

### Ualcan database

Ualcan database (http://ualcan.path.uab.edu) [26] was used to explore the expression of MTFR2, and the correlation between the level of MTFR2 with the clinicopathological parameters (including sex, age, race, smoking history, stage, histological subtype, etc.) in LUAD.

### TCGA and Kaplan-Meier plotter database

The gene expression data of 594 cases of LUAD with HTSeq-FPKM were downloaded from TCGA (https://portal.gdc.cancer.gov/projects/) [27], including 59 cases of normal lung tissues and 535 cases of LUAD tissues. Among them, the gene expression data of 57 normal lung tissues and 57 LUAD tissues came from the same patients. TCGA data were used to analyze the expression of MTFR2 and its diagnostic value via the ROC analysis.

The clinical data of 522 patients with LUAD were downloaded and screened. The patients with unknown or incomplete clinical characteristic information and lack of prognostic follow-up data were excluded, and the rest were retained for the survival analysis, univariate Cox analysis and multivariate Cox analysis. The mRNA data for Kaplan-Meier Plotter (http://kmplot.com/analysis/) analysis [28] were derived from TCGA and GEO databases. According to the median value of MTFR2 mRNA level, the data were divided into two groups: high and low expression groups to explore the correlation with prognosis.

### Screening of MTFR2 co-expressed genes

Pearson coefficient (r) was applied to indicate the correlation between genes and reflect the biological relationship between the two genes. MTFR2 co-expressed genes were screened in 535 cases of LUAD tissues from TCGA via the R (version 3.6.1) [29]. Screening criteria: *P* < 0.001 and Pearson coefficient (r > 0.4 or r < − 0.4), were defined as moderate or above.

### GO, KEGG and GSEA

The co-expressed genes of MTFR2 were analyzed by GO and KEGG [30] using R clusterProfiler package to explore biological function and relevant signal pathways in LUAD [27]. The gene expression data of 535 patients with LUAD from TCGA were divided into high and low expression groups according to the median MTFR2 level. The genes were arranged according to the degree of differential expression by GSEA (version 4.0.1) to explore the effect of MTFR2 on each gene. Each analysis was repeated 1000 times [29, 31]. Screening criteria: NOM *p* < 0.05.

### PPI network construction

STRING (https://string-db.org/) [32] database was used to analyze the protein-protein interaction (PPI) network. Screening condition: combined score > 0.9 was considered to be statistically significant. The obtained PPI network was introduced into Cytoscape 3.6.1 software, and the top 10 genes with high connectivity were screened by CytoHubba plug-in, and were defined as Hub genes [33].

### GEPIA database

The mRNA data from TCGA and GTEx, and data of normal lung tissues from GEPIA database were selected to verify the expression of Hub genes in LUAD compared with normal lung tissues. According to the median value of Hub gene expressions, the data were divided into two groups: high and low expression groups, and the relationships between their levels and OS and DFS were analyzed.

## Supplementary Information

**Additional file 1: Table S1**. Co-expressed gene of MTFR2.

**Additional file 2: Table S2**. GO analysis of MTFR2 co-expressed genes.

**Additional file 3: Figure S1**. PPI network displayed the relationship among MTFR2 coexpressed genes.

## Data Availability

Publicly available datasets were analyzed in this study. The datasets generated for this study could be found in TCGA (https://portal.gdc.cancer.gov/projects/TCGA-LUAD (HTSeq-FPKM)).

## Abbreviations

BP: Biological processes
CC: Cellular component
MF: Molecular function
OS: Overall survival
DFS: Disease-free survival
GEPIA: Gene Expression Profiling Interactive Analysis
MTFR2: Mitochondrial fission regulator 2
LUAD: Lung adenocarcinoma
NSCLC: Non-small cell lung cancer
EMT: Epithelial-stromal transformation
GSC: Glioma stem-like cells
RPA1: Replication protein A1
HR: Homologous recombination
RFS: Relapse-free survival
PLK1: Polo-likekinase1
CTCL: Cutaneous T-cell lymphomas
CDK: Cyclin-dependent kinase
