# Supplementary material for: Evaluation of clinical value and potential mechanism of MTFR2 in lung adenocarcinoma via bioinformatics
Source: BMC Cancer. 2021 May 26;21:619. doi: 10.1186/s12885-021-08378-3 (PMC8157440; doi:10.1186/s12885-021-08378-3)
Supplement: Supplementary file 1 — Additional file 1: Table S1. Co-expressed gene of MTFR2. [file 12885_2021_8378_MOESM1_ESM.docx]

Table S1. Co-expressed gene of MTFR2.

| Corgene | Cor | *p* value | Corgene | Cor | *p* value | Corgene | Cor | *p* value |
| --- | --- | --- | --- | --- | --- | --- | --- | --- |
| CENPW | 0.876 | 1.08E-170 | PSMD2 | 0.514 | 1.97E-37 | HDDC2 | 0.415 | 1.07E-23 |
| TTK | 0.849 | 7.63E-150 | PARL | 0.514 | 1.88E-37 | MRPS12 | 0.415 | 1.11E-23 |
| NCAPH | 0.848 | 4.00E-149 | ENOPH1 | 0.513 | 3.55E-37 | HDGF | 0.415 | 1.20E-23 |
| RAD51 | 0.835 | 1.43E-140 | HSPA14 | 0.513 | 2.83E-37 | SRSF12 | 0.415 | 1.13E-23 |
| CENPA | 0.827 | 2.59E-135 | PWP1 | 0.512 | 4.96E-37 | DCK | 0.415 | 1.12E-23 |
| KIF2C | 0.817 | 2.83E-129 | NME1 | 0.512 | 4.01E-37 | CCT2 | 0.415 | 1.01E-23 |
| BUB1 | 0.817 | 1.98E-129 | HNRNPL | 0.511 | 6.74E-37 | MRPS16 | 0.415 | 1.01E-23 |
| CDCA8 | 0.816 | 5.28E-129 | NUP107 | 0.511 | 5.86E-37 | MYL6B | 0.415 | 1.03E-23 |
| HJURP | 0.814 | 7.81E-128 | NUP43 | 0.511 | 5.98E-37 | SMNDC1 | 0.414 | 1.26E-23 |
| CDCA5 | 0.814 | 1.26E-127 | PRPF4 | 0.511 | 7.18E-37 | QRSL1 | 0.414 | 1.38E-23 |
| CCNB2 | 0.814 | 4.82E-128 | MAPRE1 | 0.51 | 8.70E-37 | ERAL1 | 0.414 | 1.53E-23 |
| CDC45 | 0.813 | 3.47E-127 | SELENOI | 0.509 | 1.40E-36 | ZYG11A | 0.414 | 1.57E-23 |
| CCNA2 | 0.813 | 3.47E-127 | SLBP | 0.509 | 1.29E-36 | NELFE | 0.414 | 1.34E-23 |
| SKA3 | 0.813 | 2.54E-127 | PAK1IP1 | 0.508 | 2.26E-36 | TTI1 | 0.413 | 1.94E-23 |
| AURKB | 0.812 | 1.33E-126 | RPP30 | 0.508 | 2.21E-36 | ACP1 | 0.413 | 1.65E-23 |
| TPX2 | 0.811 | 4.27E-126 | SPINDOC | 0.508 | 1.65E-36 | HMCES | 0.413 | 1.73E-23 |
| BIRC5 | 0.811 | 2.22E-126 | C9orf40 | 0.507 | 3.04E-36 | DRG1 | 0.413 | 1.67E-23 |
| SPC25 | 0.81 | 1.47E-125 | MTIF2 | 0.506 | 4.16E-36 | RPE | 0.413 | 1.64E-23 |
| MELK | 0.81 | 1.86E-125 | UCK2 | 0.506 | 3.55E-36 | NUP62 | 0.413 | 2.07E-23 |
| KIF23 | 0.809 | 7.52E-125 | MAP6D1 | 0.506 | 4.58E-36 | GLRX3 | 0.412 | 2.61E-23 |
| MCM10 | 0.808 | 2.66E-124 | TIMM23 | 0.506 | 4.37E-36 | RRP36 | 0.412 | 2.56E-23 |
| RAD51AP1 | 0.808 | 2.42E-124 | GAPDH | 0.505 | 4.80E-36 | PDCD2L | 0.412 | 2.33E-23 |
| PLK1 | 0.806 | 2.64E-123 | MSH6 | 0.504 | 7.50E-36 | SLC5A6 | 0.412 | 2.51E-23 |
| NCAPG | 0.804 | 1.30E-122 | XPOT | 0.504 | 8.88E-36 | LRPPRC | 0.412 | 2.58E-23 |
| NEK2 | 0.804 | 2.12E-122 | PPIH | 0.503 | 1.39E-35 | CEP78 | 0.412 | 2.66E-23 |
| AUNIP | 0.804 | 1.76E-122 | MRTO4 | 0.502 | 1.83E-35 | USP1 | 0.412 | 2.23E-23 |
| BUB1B | 0.804 | 2.20E-122 | POP1 | 0.502 | 1.42E-35 | RCOR2 | 0.412 | 2.49E-23 |
| KIF4A | 0.801 | 5.97E-121 | NDUFA9 | 0.502 | 1.44E-35 | ERO1A | 0.412 | 2.23E-23 |
| CDCA3 | 0.801 | 6.45E-121 | NABP2 | 0.502 | 1.64E-35 | BUD31 | 0.411 | 3.08E-23 |
| CDC20 | 0.8 | 2.27E-120 | MAGOH | 0.502 | 1.94E-35 | VPS29 | 0.411 | 3.25E-23 |
| NUF2 | 0.8 | 3.07E-120 | SSB | 0.501 | 2.79E-35 | MRPL9 | 0.411 | 3.08E-23 |
| CKAP2L | 0.798 | 1.97E-119 | ATAD5 | 0.501 | 2.51E-35 | ELOC | 0.411 | 3.33E-23 |
| MYBL2 | 0.796 | 1.80E-118 | DIABLO | 0.501 | 2.79E-35 | PHB | 0.411 | 3.18E-23 |
| SGO1 | 0.796 | 2.41E-118 | MYO19 | 0.501 | 2.75E-35 | ZFAND2A | 0.411 | 3.13E-23 |
| TROAP | 0.795 | 8.29E-118 | TMEM38B | 0.5 | 3.02E-35 | GLRX5 | 0.411 | 2.97E-23 |
| MAD2L1 | 0.795 | 6.65E-118 | PHF19 | 0.5 | 3.54E-35 | ARNTL2 | 0.41 | 4.09E-23 |
| CDC6 | 0.794 | 2.41E-117 | CCT6A | 0.5 | 3.03E-35 | CBX1 | 0.41 | 4.42E-23 |
| SKA1 | 0.794 | 1.99E-117 | ZC3H15 | 0.499 | 4.71E-35 | CDK4 | 0.41 | 4.60E-23 |
| NDC80 | 0.793 | 6.02E-117 | SMS | 0.499 | 4.43E-35 | IGF2BP1 | 0.41 | 4.30E-23 |
| CDK1 | 0.792 | 2.73E-116 | TSN | 0.499 | 5.71E-35 | MAGEA6 | 0.41 | 4.49E-23 |
| CEP55 | 0.789 | 4.48E-115 | LIG1 | 0.498 | 6.73E-35 | METTL2A | 0.409 | 5.02E-23 |
| GTSE1 | 0.788 | 3.69E-114 | PPAT | 0.498 | 6.29E-35 | HMGXB4 | 0.409 | 5.82E-23 |
| ORC6 | 0.787 | 5.76E-114 | RHEBL1 | 0.498 | 7.98E-35 | EIF3B | 0.409 | 5.59E-23 |
| NUSAP1 | 0.787 | 6.40E-114 | GPN1 | 0.498 | 6.52E-35 | ELOVL4 | 0.409 | 5.79E-23 |
| RACGAP1 | 0.787 | 9.48E-114 | WASF1 | 0.497 | 9.35E-35 | MTA2 | 0.409 | 5.96E-23 |
| PIMREG | 0.786 | 1.69E-113 | MMS22L | 0.497 | 8.54E-35 | RUVBL2 | 0.409 | 5.41E-23 |
| CCNB1 | 0.785 | 7.04E-113 | R3HDM1 | 0.496 | 1.37E-34 | ALG3 | 0.409 | 5.08E-23 |
| SPAG5 | 0.784 | 2.57E-112 | ASNS | 0.495 | 1.84E-34 | MRPL51 | 0.408 | 7.64E-23 |
| KIFC1 | 0.784 | 2.14E-112 | DUS4L | 0.495 | 2.18E-34 | RNF7 | 0.408 | 7.70E-23 |
| ORC1 | 0.783 | 4.53E-112 | C19orf48 | 0.495 | 1.75E-34 | SLIRP | 0.408 | 8.04E-23 |
| RRM2 | 0.782 | 1.73E-111 | LSG1 | 0.494 | 3.09E-34 | VPS25 | 0.408 | 8.03E-23 |
| CDKN3 | 0.78 | 2.14E-110 | UFD1 | 0.494 | 2.87E-34 | DPH2 | 0.408 | 6.51E-23 |
| CDC25C | 0.78 | 1.68E-110 | GLA | 0.494 | 2.61E-34 | PPP4C | 0.408 | 7.82E-23 |
| RAD54L | 0.779 | 2.76E-110 | PPIF | 0.494 | 3.33E-34 | MGME1 | 0.407 | 8.75E-23 |
| DLGAP5 | 0.779 | 3.15E-110 | POP7 | 0.494 | 2.84E-34 | REPS1 | 0.407 | 1.02E-22 |
| EXO1 | 0.777 | 4.87E-109 | STRAP | 0.493 | 3.62E-34 | CLPB | 0.407 | 8.30E-23 |
| FBXO5 | 0.775 | 1.97E-108 | RTN4IP1 | 0.493 | 4.22E-34 | WSB2 | 0.407 | 8.15E-23 |
| CIP2A | 0.775 | 3.98E-108 | RSRC1 | 0.493 | 4.42E-34 | GATC | 0.407 | 9.95E-23 |
| DEPDC1 | 0.774 | 7.72E-108 | UBE2N | 0.493 | 4.00E-34 | NUDT15 | 0.406 | 1.36E-22 |
| KPNA2 | 0.774 | 6.23E-108 | CEP57L1 | 0.493 | 4.01E-34 | GCLC | 0.405 | 1.47E-22 |
| PRC1 | 0.774 | 7.02E-108 | HPDL | 0.493 | 3.61E-34 | G2E3 | 0.405 | 1.75E-22 |
| TRIP13 | 0.773 | 3.17E-107 | PTGES3 | 0.492 | 5.00E-34 | RAD21 | 0.405 | 1.71E-22 |
| OIP5 | 0.773 | 1.56E-107 | UMPS | 0.492 | 6.59E-34 | MRPL48 | 0.405 | 1.59E-22 |
| FOXM1 | 0.772 | 6.13E-107 | WDR75 | 0.492 | 6.64E-34 | RCC2 | 0.405 | 1.59E-22 |
| KIF18B | 0.772 | 4.02E-107 | NOL10 | 0.492 | 5.20E-34 | TXNRD1 | 0.405 | 1.53E-22 |
| PRR11 | 0.771 | 1.16E-106 | NRM | 0.491 | 8.60E-34 | TRMT6 | 0.404 | 2.00E-22 |
| ZWINT | 0.77 | 6.22E-106 | GTF2E1 | 0.491 | 9.11E-34 | DNMT1 | 0.404 | 1.82E-22 |
| RFC4 | 0.765 | 5.47E-104 | RPA3 | 0.489 | 1.50E-33 | C12orf29 | 0.404 | 1.85E-22 |
| AURKA | 0.764 | 2.25E-103 | RAD51C | 0.489 | 1.54E-33 | HAUS2 | 0.404 | 1.98E-22 |
| DSCC1 | 0.763 | 4.69E-103 | TPI1 | 0.488 | 2.73E-33 | COPS3 | 0.404 | 2.16E-22 |
| ESPL1 | 0.762 | 7.38E-103 | SSX2IP | 0.488 | 2.61E-33 | SUMO2 | 0.404 | 1.93E-22 |
| KIF11 | 0.758 | 4.45E-101 | PSMB2 | 0.487 | 3.41E-33 | PPIA | 0.404 | 2.07E-22 |
| TRAIP | 0.758 | 6.82E-101 | PARP2 | 0.487 | 3.88E-33 | SNRNP40 | 0.403 | 2.61E-22 |
| TOP2A | 0.756 | 3.12E-100 | BRI3BP | 0.487 | 2.83E-33 | KHDRBS1 | 0.403 | 2.42E-22 |
| ASF1B | 0.755 | 1.46E-99 | MRPL21 | 0.487 | 3.30E-33 | SLC25A19 | 0.403 | 2.94E-22 |
| UBE2T | 0.754 | 2.26E-99 | TBPL1 | 0.486 | 4.14E-33 | NEDD1 | 0.403 | 2.95E-22 |
| DDIAS | 0.752 | 1.46E-98 | MRGBP | 0.486 | 4.33E-33 | ZP3 | 0.403 | 2.94E-22 |
| GINS1 | 0.751 | 2.93E-98 | NOL11 | 0.486 | 4.55E-33 | COX7A2 | 0.402 | 3.74E-22 |
| EME1 | 0.751 | 4.68E-98 | TONSL | 0.486 | 4.13E-33 | PDCL3 | 0.402 | 3.47E-22 |
| MCM6 | 0.748 | 6.66E-97 | CMAS | 0.485 | 7.52E-33 | SRM | 0.402 | 3.77E-22 |
| UBE2C | 0.747 | 2.16E-96 | CHAC2 | 0.485 | 6.37E-33 | RINT1 | 0.402 | 3.34E-22 |
| CDC25A | 0.745 | 8.14E-96 | MED27 | 0.485 | 5.67E-33 | GEMIN7 | 0.402 | 3.21E-22 |
| KIF20A | 0.743 | 3.73E-95 | EED | 0.484 | 9.81E-33 | PFDN2 | 0.402 | 3.31E-22 |
| CENPI | 0.742 | 1.38E-94 | WDR12 | 0.484 | 9.79E-33 | NOCT | 0.402 | 3.44E-22 |
| CENPN | 0.74 | 8.46E-94 | TPM3 | 0.484 | 9.66E-33 | SET | 0.401 | 4.17E-22 |
| KIF15 | 0.738 | 6.11E-93 | WDR43 | 0.484 | 8.43E-33 | SNRPD2 | 0.401 | 4.68E-22 |
| DEPDC1B | 0.737 | 9.22E-93 | RNASEH1 | 0.484 | 7.78E-33 | RCCD1 | 0.401 | 4.64E-22 |
| PKMYT1 | 0.737 | 1.47E-92 | PSMD12 | 0.484 | 7.99E-33 | PRPF40A | 0.401 | 4.23E-22 |
| PCLAF | 0.737 | 6.60E-93 | INTS13 | 0.483 | 1.34E-32 | MLPH | -0.401 | 4.61E-22 |
| PBK | 0.737 | 8.90E-93 | LRRC59 | 0.482 | 1.66E-32 | SPINK5 | -0.401 | 4.43E-22 |
| PLK4 | 0.736 | 2.08E-92 | ARL6IP1 | 0.482 | 1.82E-32 | MEGF6 | -0.401 | 4.84E-22 |
| SPC24 | 0.736 | 1.79E-92 | RAE1 | 0.481 | 2.19E-32 | AMIGO1 | -0.401 | 4.46E-22 |
| HMMR | 0.735 | 3.75E-92 | CACYBP | 0.481 | 2.75E-32 | REEP5 | -0.402 | 3.47E-22 |
| FANCI | 0.734 | 8.95E-92 | HAUS8 | 0.481 | 2.57E-32 | AOC3 | -0.402 | 3.81E-22 |
| POC1A | 0.734 | 1.23E-91 | YWHAQ | 0.481 | 2.34E-32 | BTNL9 | -0.402 | 3.32E-22 |
| SGO2 | 0.732 | 7.55E-91 | NIFK | 0.481 | 2.43E-32 | CD55 | -0.402 | 3.03E-22 |
| PARPBP | 0.732 | 1.01E-90 | TACO1 | 0.48 | 3.90E-32 | RASSF5 | -0.403 | 2.63E-22 |
| CENPU | 0.73 | 3.74E-90 | HAUS6 | 0.48 | 3.36E-32 | SECISBP2L | -0.404 | 2.04E-22 |
| CHEK1 | 0.729 | 9.82E-90 | VRK2 | 0.479 | 4.14E-32 | CYP4V2 | -0.404 | 2.21E-22 |
| ANLN | 0.722 | 3.65E-87 | EIF2B1 | 0.479 | 4.21E-32 | PAM | -0.404 | 2.02E-22 |
| SHCBP1 | 0.722 | 2.93E-87 | BCL2L12 | 0.479 | 5.45E-32 | VAMP2 | -0.404 | 1.79E-22 |
| DBF4 | 0.721 | 7.77E-87 | PSME3 | 0.479 | 5.18E-32 | C3 | -0.405 | 1.53E-22 |
| GINS2 | 0.721 | 4.14E-87 | APOBEC3B | 0.479 | 5.44E-32 | MFSD2A | -0.405 | 1.66E-22 |
| PSRC1 | 0.72 | 1.00E-86 | PUS1 | 0.478 | 7.40E-32 | PXMP4 | -0.406 | 1.31E-22 |
| CDT1 | 0.72 | 1.02E-86 | IFRD1 | 0.477 | 8.32E-32 | IL33 | -0.406 | 1.29E-22 |
| ARHGAP11A | 0.715 | 4.79E-85 | PLOD2 | 0.477 | 9.77E-32 | SMAD6 | -0.406 | 1.09E-22 |
| TICRR | 0.714 | 1.55E-84 | SENP1 | 0.476 | 1.46E-31 | FCGBP | -0.406 | 1.30E-22 |
| TYMS | 0.714 | 1.47E-84 | TEAD4 | 0.476 | 1.35E-31 | PYROXD2 | -0.408 | 6.79E-23 |
| TACC3 | 0.713 | 4.16E-84 | CUL2 | 0.475 | 2.06E-31 | AFF3 | -0.408 | 7.26E-23 |
| MCM2 | 0.713 | 2.59E-84 | PSMD3 | 0.475 | 1.58E-31 | GFRA1 | -0.408 | 6.54E-23 |
| CDCA2 | 0.713 | 3.87E-84 | CCT7 | 0.475 | 1.66E-31 | ALPL | -0.408 | 6.88E-23 |
| FEN1 | 0.707 | 2.51E-82 | TWF1 | 0.475 | 1.79E-31 | PLA2G4F | -0.408 | 7.65E-23 |
| FAM83D | 0.703 | 5.64E-81 | ZUP1 | 0.475 | 1.87E-31 | EMP2 | -0.408 | 6.30E-23 |
| VRK1 | 0.702 | 1.78E-80 | SNF8 | 0.475 | 1.76E-31 | KLF15 | -0.409 | 5.03E-23 |
| MCM4 | 0.702 | 1.81E-80 | PPP1CC | 0.475 | 1.67E-31 | BORCS7 | -0.409 | 5.66E-23 |
| C17orf53 | 0.702 | 1.25E-80 | KATNA1 | 0.475 | 1.87E-31 | MARF1 | -0.409 | 6.19E-23 |
| DTL | 0.702 | 1.80E-80 | DARS | 0.474 | 2.35E-31 | EPDR1 | -0.41 | 3.96E-23 |
| H2AFZ | 0.702 | 1.71E-80 | RHNO1 | 0.474 | 2.23E-31 | TMEM243 | -0.41 | 3.85E-23 |
| STIL | 0.7 | 5.67E-80 | MRPL13 | 0.474 | 2.81E-31 | FBLN5 | -0.41 | 4.68E-23 |
| ERCC6L | 0.7 | 7.34E-80 | TTL | 0.473 | 3.27E-31 | CES2 | -0.41 | 4.02E-23 |
| XRCC2 | 0.7 | 6.47E-80 | TTF2 | 0.473 | 3.27E-31 | ALOX15B | -0.41 | 4.50E-23 |
| HASPIN | 0.699 | 1.17E-79 | IMMT | 0.473 | 3.46E-31 | EFCAB14 | -0.411 | 3.33E-23 |
| KNSTRN | 0.697 | 3.66E-79 | KIF3C | 0.471 | 7.78E-31 | MACROD2 | -0.411 | 3.23E-23 |
| RFC5 | 0.694 | 4.50E-78 | GTPBP4 | 0.471 | 7.22E-31 | DISP1 | -0.412 | 2.63E-23 |
| ESCO2 | 0.693 | 8.89E-78 | MRPL15 | 0.471 | 7.53E-31 | TNXB | -0.412 | 2.59E-23 |
| RFC3 | 0.692 | 1.44E-77 | PCGF6 | 0.471 | 6.27E-31 | CST5 | -0.412 | 2.35E-23 |
| ZWILCH | 0.692 | 1.98E-77 | SKA2 | 0.471 | 6.03E-31 | TPPP | -0.412 | 2.51E-23 |
| NCAPG2 | 0.691 | 3.87E-77 | PSMG1 | 0.471 | 5.93E-31 | SLC41A1 | -0.413 | 1.84E-23 |
| UBE2S | 0.689 | 1.69E-76 | NUP155 | 0.47 | 1.05E-30 | RNF145 | -0.413 | 1.85E-23 |
| DIAPH3 | 0.689 | 1.77E-76 | ACTR3 | 0.47 | 8.03E-31 | WWP2 | -0.413 | 1.71E-23 |
| PRIM1 | 0.689 | 1.24E-76 | NAA50 | 0.47 | 9.03E-31 | AC012651.1 | -0.413 | 1.98E-23 |
| CENPM | 0.688 | 2.95E-76 | RFWD3 | 0.47 | 1.08E-30 | NKX2-1 | -0.414 | 1.53E-23 |
| BRCA1 | 0.687 | 6.15E-76 | GPI | 0.469 | 1.13E-30 | SGMS2 | -0.414 | 1.42E-23 |
| TEDC2 | 0.687 | 6.83E-76 | CCDC77 | 0.469 | 1.46E-30 | B3GNT8 | -0.414 | 1.51E-23 |
| TK1 | 0.687 | 4.73E-76 | RNF34 | 0.469 | 1.43E-30 | HSD17B6 | -0.415 | 9.90E-24 |
| CKS1B | 0.687 | 8.66E-76 | GEN1 | 0.469 | 1.27E-30 | EDNRB | -0.415 | 9.61E-24 |
| CENPH | 0.686 | 1.51E-75 | DDX55 | 0.468 | 1.66E-30 | TK2 | -0.415 | 1.04E-23 |
| KIF14 | 0.685 | 3.09E-75 | BARD1 | 0.468 | 2.01E-30 | GKN2 | -0.416 | 7.70E-24 |
| TCP1 | 0.685 | 2.78E-75 | EIPR1 | 0.467 | 2.50E-30 | CTSE | -0.416 | 8.08E-24 |
| BLM | 0.685 | 2.86E-75 | COCH | 0.467 | 2.61E-30 | ADAMTSL2 | -0.416 | 8.27E-24 |
| CDK2 | 0.682 | 2.32E-74 | MAGOHB | 0.467 | 2.51E-30 | PTPRE | -0.417 | 6.14E-24 |
| MCM7 | 0.682 | 2.58E-74 | MSANTD3 | 0.466 | 2.96E-30 | MYH11 | -0.417 | 7.08E-24 |
| RNASEH2A | 0.681 | 4.90E-74 | BOLA2-SMG1P6 | 0.466 | 2.95E-30 | SFTPA2 | -0.417 | 6.49E-24 |
| CENPE | 0.681 | 2.78E-74 | GNG4 | 0.465 | 4.19E-30 | ACSL5 | -0.417 | 6.99E-24 |
| MND1 | 0.676 | 9.74E-73 | NIF3L1 | 0.465 | 4.63E-30 | SLC44A4 | -0.417 | 6.67E-24 |
| CHAF1B | 0.676 | 1.21E-72 | MRPS35 | 0.464 | 6.15E-30 | CST3 | -0.418 | 5.54E-24 |
| FANCG | 0.675 | 2.06E-72 | BCCIP | 0.464 | 6.27E-30 | ARHGEF17 | -0.418 | 4.47E-24 |
| GINS4 | 0.671 | 2.41E-71 | PDCD10 | 0.464 | 7.42E-30 | SORCS2 | -0.418 | 4.94E-24 |
| MASTL | 0.67 | 7.44E-71 | SNRPB | 0.464 | 6.35E-30 | SYNE1 | -0.419 | 3.66E-24 |
| CENPO | 0.67 | 4.97E-71 | ORC5 | 0.464 | 5.91E-30 | FAM189A2 | -0.419 | 3.67E-24 |
| POLQ | 0.669 | 1.46E-70 | FIGNL1 | 0.463 | 8.75E-30 | DAPK2 | -0.42 | 2.60E-24 |
| WDR76 | 0.669 | 8.86E-71 | YWHAG | 0.463 | 8.62E-30 | CD81 | -0.42 | 2.88E-24 |
| TIMELESS | 0.669 | 9.67E-71 | CCDC43 | 0.463 | 1.01E-29 | EPHX2 | -0.42 | 3.06E-24 |
| CENPK | 0.667 | 3.49E-70 | HNRNPC | 0.462 | 1.15E-29 | ARHGAP24 | -0.42 | 2.74E-24 |
| E2F1 | 0.665 | 1.50E-69 | SLC7A5 | 0.462 | 1.33E-29 | CD1C | -0.42 | 2.79E-24 |
| PSMC3IP | 0.665 | 1.23E-69 | NUDCD1 | 0.462 | 1.05E-29 | CDKL2 | -0.421 | 2.29E-24 |
| KNL1 | 0.665 | 1.26E-69 | FAM133A | 0.462 | 1.16E-29 | DAAM2 | -0.421 | 2.42E-24 |
| UHRF1 | 0.665 | 1.61E-69 | DNAJB11 | 0.461 | 1.85E-29 | SIRT3 | -0.422 | 1.76E-24 |
| RANBP1 | 0.663 | 4.95E-69 | CSTF2 | 0.461 | 1.56E-29 | USP54 | -0.422 | 1.55E-24 |
| CCNE1 | 0.663 | 3.86E-69 | CCDC86 | 0.461 | 1.74E-29 | LIMD1 | -0.423 | 1.37E-24 |
| MKI67 | 0.663 | 4.75E-69 | POLR3G | 0.461 | 1.47E-29 | KLF2 | -0.424 | 1.01E-24 |
| CDCA4 | 0.663 | 4.03E-69 | RPP40 | 0.461 | 1.45E-29 | FCHO2 | -0.424 | 9.27E-25 |
| EZH2 | 0.662 | 8.56E-69 | RBM17 | 0.461 | 1.58E-29 | PHACTR1 | -0.425 | 7.69E-25 |
| E2F2 | 0.66 | 3.52E-68 | PSMA5 | 0.461 | 1.83E-29 | PDZD2 | -0.425 | 7.50E-25 |
| DTYMK | 0.66 | 3.07E-68 | CDC123 | 0.461 | 1.81E-29 | ADAMTSL3 | -0.425 | 6.57E-25 |
| MTHFD2 | 0.659 | 4.60E-68 | COPS6 | 0.461 | 1.91E-29 | CPAMD8 | -0.425 | 7.61E-25 |
| POLE2 | 0.659 | 7.62E-68 | PSMB3 | 0.461 | 1.45E-29 | IRX5 | -0.425 | 7.53E-25 |
| CKAP2 | 0.659 | 7.57E-68 | RAB10 | 0.46 | 2.39E-29 | NPC2 | -0.426 | 5.30E-25 |
| FANCD2 | 0.659 | 5.19E-68 | PGAM1 | 0.46 | 2.04E-29 | SFTPA1 | -0.426 | 5.63E-25 |
| PDSS1 | 0.659 | 6.75E-68 | STIP1 | 0.459 | 3.18E-29 | ATP13A4 | -0.426 | 5.07E-25 |
| POLA2 | 0.658 | 1.05E-67 | B3GNT5 | 0.459 | 3.31E-29 | CD1E | -0.426 | 5.83E-25 |
| E2F8 | 0.657 | 2.10E-67 | UCHL3 | 0.458 | 4.15E-29 | CD207 | -0.427 | 4.24E-25 |
| DNAJC9 | 0.657 | 2.77E-67 | CDK8 | 0.458 | 3.81E-29 | CAVIN2 | -0.427 | 4.11E-25 |
| NUP37 | 0.656 | 4.19E-67 | KCMF1 | 0.458 | 3.96E-29 | C9orf152 | -0.427 | 3.68E-25 |
| BORA | 0.654 | 1.59E-66 | RCC1 | 0.458 | 3.98E-29 | TXNIP | -0.427 | 3.70E-25 |
| MIS18A | 0.653 | 1.99E-66 | EBNA1BP2 | 0.457 | 5.32E-29 | ARHGAP44 | -0.429 | 2.16E-25 |
| IQGAP3 | 0.653 | 3.25E-66 | NDUFA12 | 0.457 | 5.54E-29 | SLC46A2 | -0.429 | 2.19E-25 |
| KIF18A | 0.652 | 5.98E-66 | NOP56 | 0.456 | 6.88E-29 | ALDH2 | -0.43 | 1.59E-25 |
| CENPL | 0.65 | 1.26E-65 | STOML2 | 0.456 | 8.79E-29 | TCF21 | -0.43 | 1.72E-25 |
| CLSPN | 0.649 | 2.59E-65 | STMP1 | 0.456 | 7.27E-29 | FLRT3 | -0.43 | 1.81E-25 |
| TMPO | 0.649 | 2.79E-65 | BRCA2 | 0.455 | 1.11E-28 | SPARCL1 | -0.43 | 1.79E-25 |
| RFC2 | 0.647 | 1.15E-64 | KIF24 | 0.455 | 1.23E-28 | ATOH8 | -0.43 | 1.91E-25 |
| SUV39H1 | 0.647 | 9.28E-65 | SEM1 | 0.454 | 1.37E-28 | KCNQ1 | -0.431 | 1.17E-25 |
| CENPF | 0.647 | 8.08E-65 | ILF2 | 0.454 | 1.38E-28 | PTPRU | -0.431 | 1.26E-25 |
| DSN1 | 0.646 | 2.10E-64 | POLR2H | 0.454 | 1.54E-28 | EFCC1 | -0.431 | 1.33E-25 |
| LMNB1 | 0.643 | 1.11E-63 | SAAL1 | 0.454 | 1.53E-28 | C4BPA | -0.431 | 1.46E-25 |
| ASPM | 0.642 | 1.54E-63 | YBX1 | 0.453 | 2.14E-28 | TSPAN3 | -0.431 | 1.26E-25 |
| C1orf112 | 0.64 | 6.76E-63 | TAF6 | 0.453 | 1.87E-28 | MR1 | -0.431 | 1.33E-25 |
| ZNF367 | 0.64 | 6.65E-63 | KPNB1 | 0.453 | 1.95E-28 | CX3CR1 | -0.431 | 1.34E-25 |
| PCNA | 0.639 | 1.19E-62 | MTCH2 | 0.453 | 2.07E-28 | SMARCA2 | -0.432 | 1.04E-25 |
| ATAD2 | 0.639 | 8.80E-63 | CD3EAP | 0.453 | 2.12E-28 | VEGFD | -0.432 | 8.60E-26 |
| NDC1 | 0.637 | 2.76E-62 | VPS33A | 0.453 | 1.92E-28 | ZNF540 | -0.432 | 9.58E-26 |
| ECT2 | 0.637 | 3.29E-62 | HAUS1 | 0.453 | 1.75E-28 | PGC | -0.433 | 8.06E-26 |
| FAAP24 | 0.636 | 5.94E-62 | CEBPG | 0.453 | 2.01E-28 | NOD1 | -0.433 | 7.09E-26 |
| LTV1 | 0.636 | 4.98E-62 | USP39 | 0.453 | 1.87E-28 | CD302 | -0.433 | 7.45E-26 |
| DBF4B | 0.636 | 6.05E-62 | MTHFD1 | 0.452 | 2.56E-28 | CALCOCO1 | -0.434 | 5.52E-26 |
| ALYREF | 0.636 | 4.25E-62 | DHX37 | 0.452 | 2.51E-28 | LTBP2 | -0.434 | 5.11E-26 |
| GINS3 | 0.633 | 2.82E-61 | TMEM237 | 0.452 | 2.46E-28 | FOXA2 | -0.434 | 5.79E-26 |
| CDC7 | 0.632 | 5.61E-61 | COA7 | 0.452 | 2.79E-28 | KIAA0319L | -0.434 | 5.24E-26 |
| INCENP | 0.632 | 5.21E-61 | GEMIN2 | 0.451 | 3.39E-28 | A2M | -0.434 | 6.14E-26 |
| HDAC2 | 0.632 | 5.12E-61 | TRIM59 | 0.451 | 3.33E-28 | SEPT4 | -0.435 | 4.50E-26 |
| CCNF | 0.631 | 1.01E-60 | PSMA7 | 0.45 | 5.05E-28 | AL365205.1 | -0.435 | 3.78E-26 |
| STMN1 | 0.63 | 2.13E-60 | FBL | 0.45 | 4.94E-28 | CHIA | -0.435 | 4.17E-26 |
| CHAF1A | 0.63 | 1.40E-60 | AIMP2 | 0.45 | 5.33E-28 | ROS1 | -0.436 | 3.21E-26 |
| PIF1 | 0.629 | 3.40E-60 | EXOSC3 | 0.45 | 4.77E-28 | MECOM | -0.436 | 2.94E-26 |
| CKS2 | 0.628 | 6.29E-60 | EIF2S1 | 0.45 | 5.55E-28 | CEP112 | -0.436 | 2.76E-26 |
| MRPL47 | 0.625 | 2.73E-59 | MRPS22 | 0.45 | 4.59E-28 | ATP1A1 | -0.436 | 3.34E-26 |
| C5orf34 | 0.624 | 3.43E-59 | RPAP3 | 0.449 | 6.88E-28 | PRDM16 | -0.437 | 2.38E-26 |
| WDHD1 | 0.624 | 3.47E-59 | NSD2 | 0.449 | 7.37E-28 | IL6R | -0.438 | 1.52E-26 |
| HMGB2 | 0.621 | 2.03E-58 | CALU | 0.449 | 6.22E-28 | EVA1C | -0.439 | 1.34E-26 |
| PTTG1 | 0.621 | 2.16E-58 | ATP5MC3 | 0.449 | 7.27E-28 | REPS2 | -0.439 | 1.35E-26 |
| RPF2 | 0.621 | 2.90E-58 | C12orf43 | 0.449 | 6.21E-28 | EPHA4 | -0.44 | 8.87E-27 |
| ACTL6A | 0.619 | 7.48E-58 | CPSF4 | 0.449 | 6.14E-28 | FRY | -0.441 | 7.55E-27 |
| NCAPD2 | 0.615 | 5.75E-57 | TRIB3 | 0.448 | 9.85E-28 | CPQ | -0.441 | 7.89E-27 |
| UNG | 0.615 | 6.27E-57 | PSMC2 | 0.448 | 1.01E-27 | PTPN13 | -0.441 | 7.94E-27 |
| GMNN | 0.615 | 6.61E-57 | SHMT2 | 0.448 | 9.69E-28 | PNMA2 | -0.441 | 6.37E-27 |
| CTSV | 0.615 | 7.24E-57 | SLC25A13 | 0.447 | 1.08E-27 | RAB27A | -0.442 | 4.74E-27 |
| TUBA1B | 0.614 | 8.84E-57 | MDH1 | 0.447 | 1.40E-27 | AQP4 | -0.442 | 5.04E-27 |
| RMI2 | 0.614 | 7.83E-57 | WBP11 | 0.447 | 1.30E-27 | GTF2IRD2B | -0.442 | 5.43E-27 |
| PSMD14 | 0.613 | 1.66E-56 | PSPH | 0.447 | 1.05E-27 | IL6ST | -0.443 | 4.02E-27 |
| TUBG1 | 0.613 | 1.32E-56 | ATP1B3 | 0.446 | 1.83E-27 | CYFIP2 | -0.444 | 3.10E-27 |
| SNRPD1 | 0.613 | 1.36E-56 | SCML2 | 0.446 | 1.89E-27 | DUOXA1 | -0.444 | 3.10E-27 |
| TUBA1C | 0.612 | 2.39E-56 | MAD2L2 | 0.446 | 1.53E-27 | RPS6KA2 | -0.445 | 2.33E-27 |
| CSE1L | 0.61 | 7.56E-56 | PGP | 0.446 | 1.61E-27 | PIK3IP1 | -0.445 | 2.31E-27 |
| ARHGEF39 | 0.61 | 6.96E-56 | RAB5IF | 0.445 | 2.47E-27 | SCGB3A1 | -0.445 | 2.05E-27 |
| CCDC58 | 0.61 | 9.45E-56 | CMC2 | 0.445 | 2.39E-27 | PIGR | -0.445 | 2.06E-27 |
| RAN | 0.608 | 1.74E-55 | DEK | 0.445 | 1.99E-27 | IRX3 | -0.445 | 2.34E-27 |
| PRIM2 | 0.607 | 3.09E-55 | DYNLL1 | 0.444 | 2.63E-27 | PHKB | -0.446 | 1.54E-27 |
| RECQL4 | 0.607 | 4.62E-55 | GNPNAT1 | 0.444 | 2.65E-27 | SIAE | -0.446 | 1.89E-27 |
| FUCA2 | 0.606 | 5.42E-55 | GOLT1B | 0.444 | 3.00E-27 | GPRC5C | -0.446 | 1.61E-27 |
| VTA1 | 0.606 | 7.19E-55 | FKBP4 | 0.443 | 3.72E-27 | DOK4 | -0.447 | 1.11E-27 |
| SPDL1 | 0.605 | 9.73E-55 | GPN3 | 0.443 | 4.21E-27 | IVD | -0.447 | 1.34E-27 |
| KIF20B | 0.603 | 3.55E-54 | PFKFB4 | 0.443 | 3.76E-27 | UBL3 | -0.448 | 9.60E-28 |
| SUV39H2 | 0.6 | 1.23E-53 | CCT3 | 0.443 | 4.50E-27 | ITIH5 | -0.448 | 7.89E-28 |
| FANCA | 0.598 | 2.83E-53 | NKIRAS2 | 0.443 | 3.93E-27 | CTDSPL | -0.448 | 8.47E-28 |
| GGH | 0.596 | 1.14E-52 | HEBP2 | 0.442 | 4.66E-27 | SLC34A2 | -0.448 | 9.59E-28 |
| FBXO45 | 0.596 | 8.26E-53 | YKT6 | 0.442 | 6.03E-27 | HNF1B | -0.448 | 1.04E-27 |
| SAPCD2 | 0.594 | 2.88E-52 | TRMT10C | 0.442 | 4.97E-27 | AK1 | -0.449 | 6.62E-28 |
| FAM136A | 0.592 | 8.35E-52 | APOO | 0.442 | 4.84E-27 | FBXW4 | -0.45 | 4.58E-28 |
| DONSON | 0.592 | 7.91E-52 | TMEM185B | 0.442 | 4.69E-27 | BTG2 | -0.45 | 5.67E-28 |
| SASS6 | 0.591 | 1.16E-51 | TIMM50 | 0.441 | 7.10E-27 | ABO | -0.45 | 4.49E-28 |
| PGAM5 | 0.59 | 1.96E-51 | POMP | 0.441 | 6.83E-27 | DELE1 | -0.451 | 3.36E-28 |
| DNA2 | 0.589 | 2.61E-51 | PDRG1 | 0.44 | 1.11E-26 | LMF1 | -0.452 | 3.09E-28 |
| CMSS1 | 0.589 | 2.53E-51 | AGMAT | 0.44 | 9.46E-27 | SPRY4 | -0.452 | 2.37E-28 |
| CHCHD3 | 0.588 | 5.44E-51 | NUS1 | 0.44 | 9.45E-27 | MARC2 | -0.453 | 2.30E-28 |
| HAT1 | 0.587 | 8.42E-51 | NUP93 | 0.439 | 1.30E-26 | CAT | -0.453 | 2.25E-28 |
| CNOT9 | 0.587 | 6.91E-51 | CHTF18 | 0.439 | 1.39E-26 | AHCYL2 | -0.453 | 2.24E-28 |
| CCT5 | 0.587 | 8.18E-51 | AKIRIN2 | 0.439 | 1.16E-26 | FYCO1 | -0.453 | 1.76E-28 |
| HPRT1 | 0.587 | 8.05E-51 | DGUOK | 0.438 | 1.96E-26 | LRRK2 | -0.454 | 1.33E-28 |
| DKC1 | 0.586 | 1.13E-50 | ITGB1BP1 | 0.438 | 1.67E-26 | CGNL1 | -0.455 | 9.21E-29 |
| DENR | 0.586 | 1.49E-50 | C19orf47 | 0.438 | 1.92E-26 | DUOX1 | -0.455 | 1.16E-28 |
| EIF4EBP1 | 0.584 | 3.17E-50 | PATL1 | 0.438 | 1.75E-26 | RAI2 | -0.456 | 9.00E-29 |
| TPRKB | 0.583 | 5.80E-50 | ABCF2 | 0.437 | 2.25E-26 | XPC | -0.456 | 7.26E-29 |
| FAM111B | 0.583 | 5.32E-50 | MMD | 0.437 | 2.54E-26 | LMO3 | -0.457 | 5.75E-29 |
| BRIP1 | 0.582 | 1.01E-49 | MRPL18 | 0.437 | 2.43E-26 | RBMS3 | -0.457 | 6.51E-29 |
| CHEK2 | 0.582 | 8.00E-50 | CBX3 | 0.437 | 2.31E-26 | PARM1 | -0.457 | 5.09E-29 |
| WDR62 | 0.581 | 1.49E-49 | MAIP1 | 0.437 | 2.43E-26 | NEDD9 | -0.458 | 4.90E-29 |
| FARSB | 0.581 | 1.55E-49 | E2F6 | 0.437 | 2.11E-26 | HSD17B4 | -0.458 | 4.82E-29 |
| NUDT1 | 0.58 | 1.82E-49 | C6orf120 | 0.437 | 2.17E-26 | PLA2G10 | -0.459 | 2.68E-29 |
| SKP2 | 0.58 | 1.63E-49 | DPM1 | 0.436 | 3.03E-26 | PLAC9 | -0.459 | 3.39E-29 |
| GMPS | 0.579 | 3.09E-49 | STC2 | 0.436 | 3.28E-26 | NDNF | -0.46 | 2.38E-29 |
| NEIL3 | 0.578 | 5.09E-49 | TBC1D7 | 0.436 | 3.04E-26 | ROBO2 | -0.46 | 2.10E-29 |
| SNRPA1 | 0.577 | 7.00E-49 | NMD3 | 0.436 | 3.37E-26 | MAST4 | -0.461 | 1.72E-29 |
| H2AFX | 0.577 | 9.55E-49 | C17orf58 | 0.436 | 3.23E-26 | TGFBR2 | -0.461 | 1.84E-29 |
| HMGA1 | 0.576 | 1.07E-48 | HMBS | 0.436 | 3.45E-26 | TAPT1 | -0.462 | 1.37E-29 |
| TCF19 | 0.576 | 1.14E-48 | SAE1 | 0.435 | 4.68E-26 | PLA2G1B | -0.462 | 1.20E-29 |
| LRR1 | 0.576 | 1.13E-48 | THOP1 | 0.435 | 4.28E-26 | AQP3 | -0.463 | 7.81E-30 |
| DDX39A | 0.573 | 4.53E-48 | ADA | 0.435 | 3.81E-26 | VWA2 | -0.463 | 7.76E-30 |
| LYAR | 0.573 | 6.17E-48 | ARPC1A | 0.435 | 3.91E-26 | CELF2 | -0.464 | 5.78E-30 |
| ABRACL | 0.573 | 4.80E-48 | HLTF | 0.434 | 5.02E-26 | FMO5 | -0.464 | 6.29E-30 |
| CPSF3 | 0.572 | 9.57E-48 | TFRC | 0.434 | 5.27E-26 | ST6GALNAC6 | -0.464 | 6.66E-30 |
| SNRPF | 0.572 | 7.79E-48 | VDAC3 | 0.434 | 5.62E-26 | RAP1GAP | -0.465 | 5.40E-30 |
| PSMD11 | 0.571 | 1.22E-47 | SEPHS1 | 0.434 | 5.17E-26 | ABCA3 | -0.465 | 4.65E-30 |
| HILPDA | 0.571 | 1.35E-47 | PPIL1 | 0.434 | 5.22E-26 | SNX25 | -0.466 | 3.92E-30 |
| PDCD5 | 0.57 | 2.51E-47 | WTAP | 0.434 | 6.13E-26 | TBX4 | -0.466 | 2.91E-30 |
| RAD54B | 0.57 | 1.81E-47 | GTF3C6 | 0.434 | 5.19E-26 | ABCA8 | -0.466 | 3.34E-30 |
| TOPBP1 | 0.569 | 3.10E-47 | MYBL1 | 0.434 | 5.51E-26 | PBXIP1 | -0.466 | 3.80E-30 |
| WDR53 | 0.569 | 3.83E-47 | YBX2 | 0.433 | 7.93E-26 | CAPN8 | -0.466 | 3.69E-30 |
| C4orf46 | 0.569 | 3.06E-47 | BYSL | 0.433 | 6.62E-26 | ELN | -0.467 | 2.24E-30 |
| MSH2 | 0.568 | 5.60E-47 | EIF5A | 0.433 | 7.72E-26 | EMCN | -0.467 | 2.59E-30 |
| MCM5 | 0.568 | 5.00E-47 | NASP | 0.433 | 6.62E-26 | MFAP4 | -0.467 | 2.52E-30 |
| HELLS | 0.568 | 5.47E-47 | VBP1 | 0.433 | 7.84E-26 | BTD | -0.467 | 2.43E-30 |
| MTHFD1L | 0.568 | 4.34E-47 | CSTF3 | 0.433 | 7.63E-26 | SFTA2 | -0.467 | 2.17E-30 |
| RPL39L | 0.567 | 7.27E-47 | NRAS | 0.433 | 6.87E-26 | EDA2R | -0.468 | 1.82E-30 |
| TIPIN | 0.565 | 1.82E-46 | DUSP14 | 0.433 | 7.28E-26 | RBPMS | -0.468 | 1.60E-30 |
| TESMIN | 0.565 | 2.03E-46 | RIPK2 | 0.432 | 1.00E-25 | FCER1A | -0.468 | 1.73E-30 |
| KNTC1 | 0.565 | 1.84E-46 | ZNF639 | 0.432 | 1.03E-25 | MUC1 | -0.468 | 2.09E-30 |
| NEMP1 | 0.564 | 2.53E-46 | POPDC3 | 0.432 | 9.93E-26 | CTSO | -0.468 | 1.72E-30 |
| TOMM40 | 0.563 | 5.32E-46 | PFKP | 0.431 | 1.32E-25 | MGP | -0.47 | 8.05E-31 |
| PNPT1 | 0.563 | 5.05E-46 | FAM162A | 0.43 | 1.77E-25 | SFTPD | -0.47 | 8.44E-31 |
| RBL1 | 0.561 | 9.68E-46 | EEF1E1 | 0.43 | 1.56E-25 | MTURN | -0.47 | 9.08E-31 |
| MEST | 0.56 | 1.97E-45 | SUZ12 | 0.43 | 1.51E-25 | MAOA | -0.47 | 9.43E-31 |
| SMC2 | 0.56 | 1.63E-45 | TFG | 0.429 | 2.07E-25 | ITGA8 | -0.471 | 6.35E-31 |
| TEX30 | 0.56 | 1.67E-45 | CYREN | 0.429 | 2.02E-25 | ADAMTS8 | -0.471 | 6.12E-31 |
| BUB3 | 0.56 | 1.66E-45 | XPO5 | 0.429 | 2.27E-25 | NICN1 | -0.471 | 6.61E-31 |
| PAICS | 0.557 | 6.06E-45 | ABCE1 | 0.429 | 2.17E-25 | CREBRF | -0.472 | 5.61E-31 |
| TBC1D31 | 0.557 | 6.88E-45 | GTF3C3 | 0.428 | 3.20E-25 | CBX7 | -0.475 | 1.85E-31 |
| CENPQ | 0.556 | 7.90E-45 | SPATS2 | 0.428 | 3.14E-25 | RNASE1 | -0.476 | 1.22E-31 |
| PCMT1 | 0.556 | 8.14E-45 | POLE3 | 0.428 | 3.16E-25 | AKAP13 | -0.476 | 1.22E-31 |
| NUP85 | 0.556 | 8.29E-45 | SRSF2 | 0.428 | 2.90E-25 | RMDN2 | -0.477 | 1.03E-31 |
| EIF2S2 | 0.556 | 9.89E-45 | OPA1 | 0.428 | 2.74E-25 | PLLP | -0.479 | 4.88E-32 |
| E2F7 | 0.556 | 1.06E-44 | AP2S1 | 0.427 | 3.64E-25 | PEBP4 | -0.479 | 5.57E-32 |
| TUBB | 0.555 | 1.23E-44 | MRPS10 | 0.427 | 3.58E-25 | GNG7 | -0.479 | 5.12E-32 |
| LMNB2 | 0.554 | 1.96E-44 | MTRF1L | 0.427 | 4.31E-25 | ESYT3 | -0.481 | 2.48E-32 |
| BRIX1 | 0.553 | 3.09E-44 | GART | 0.427 | 4.56E-25 | NIPAL3 | -0.482 | 1.79E-32 |
| CGAS | 0.553 | 4.15E-44 | ADSL | 0.427 | 4.54E-25 | SCN4B | -0.484 | 8.51E-33 |
| UBA2 | 0.551 | 7.69E-44 | NOP58 | 0.426 | 5.46E-25 | CIRBP | -0.485 | 7.28E-33 |
| DNMT3B | 0.547 | 3.97E-43 | USP14 | 0.426 | 5.64E-25 | CTSH | -0.485 | 6.85E-33 |
| COQ3 | 0.547 | 4.58E-43 | E2F3 | 0.426 | 4.70E-25 | C7 | -0.485 | 7.43E-33 |
| HSPD1 | 0.547 | 4.96E-43 | RTCA | 0.426 | 5.82E-25 | ATP8A1 | -0.485 | 5.96E-33 |
| TOMM5 | 0.546 | 7.00E-43 | DNAJC2 | 0.425 | 6.92E-25 | PLXNA2 | -0.487 | 2.95E-33 |
| MCM3 | 0.545 | 1.17E-42 | PRPF19 | 0.425 | 6.70E-25 | SCNN1B | -0.487 | 3.18E-33 |
| MTBP | 0.545 | 8.79E-43 | GTF2H3 | 0.425 | 7.50E-25 | ABCC6 | -0.488 | 2.74E-33 |
| MRPL11 | 0.545 | 9.64E-43 | SMC4 | 0.425 | 7.04E-25 | TMEM173 | -0.489 | 1.42E-33 |
| LIN9 | 0.545 | 9.23E-43 | SSRP1 | 0.425 | 7.97E-25 | SHH | -0.49 | 1.13E-33 |
| TMEM206 | 0.544 | 1.58E-42 | MTERF3 | 0.425 | 6.93E-25 | TMEM125 | -0.49 | 1.13E-33 |
| MCM8 | 0.544 | 1.31E-42 | DCAF13 | 0.425 | 6.72E-25 | MGLL | -0.491 | 8.74E-34 |
| UTP6 | 0.543 | 1.97E-42 | PDCD2 | 0.424 | 8.82E-25 | ADGRD1 | -0.492 | 4.91E-34 |
| TDG | 0.541 | 5.26E-42 | XPO1 | 0.424 | 9.67E-25 | SLC27A1 | -0.492 | 5.22E-34 |
| ECE2 | 0.541 | 4.40E-42 | PDAP1 | 0.424 | 9.46E-25 | CISH | -0.493 | 3.73E-34 |
| PRTFDC1 | 0.54 | 6.98E-42 | DCLRE1B | 0.424 | 1.05E-24 | ENTPD3 | -0.495 | 2.17E-34 |
| GAS2L3 | 0.539 | 1.36E-41 | NDUFAF4 | 0.424 | 9.30E-25 | SHE | -0.495 | 2.09E-34 |
| CTPS1 | 0.538 | 1.49E-41 | ATIC | 0.424 | 1.05E-24 | SHROOM4 | -0.496 | 1.68E-34 |
| ACAT2 | 0.535 | 6.77E-41 | KPNA4 | 0.424 | 1.03E-24 | SPATA18 | -0.498 | 7.95E-35 |
| C18orf54 | 0.535 | 6.38E-41 | ACOT7 | 0.423 | 1.18E-24 | SLC26A9 | -0.498 | 8.09E-35 |
| EFTUD2 | 0.534 | 9.99E-41 | PSMB5 | 0.423 | 1.38E-24 | VSIG2 | -0.499 | 4.24E-35 |
| ANAPC7 | 0.534 | 1.02E-40 | RIOK1 | 0.423 | 1.29E-24 | SLC22A31 | -0.499 | 4.27E-35 |
| OLA1 | 0.533 | 1.45E-40 | RNFT2 | 0.423 | 1.27E-24 | IGIP | -0.501 | 2.86E-35 |
| NCAPD3 | 0.533 | 1.29E-40 | SMPD4 | 0.423 | 1.14E-24 | GANC | -0.501 | 2.85E-35 |
| SRSF9 | 0.532 | 2.43E-40 | UBE2F | 0.423 | 1.09E-24 | FOLR1 | -0.504 | 9.51E-36 |
| RUVBL1 | 0.532 | 2.44E-40 | TFDP1 | 0.423 | 1.24E-24 | C1QTNF7 | -0.504 | 7.84E-36 |
| MZT1 | 0.532 | 1.75E-40 | MMP12 | 0.423 | 1.23E-24 | CYBRD1 | -0.505 | 6.42E-36 |
| KIF22 | 0.53 | 4.84E-40 | PRMT1 | 0.422 | 1.47E-24 | SCTR | -0.505 | 5.12E-36 |
| LSM12 | 0.53 | 3.76E-40 | PREB | 0.422 | 1.62E-24 | HLF | -0.506 | 4.49E-36 |
| PSAT1 | 0.529 | 7.38E-40 | CCT8 | 0.422 | 1.48E-24 | MAMDC2 | -0.506 | 4.22E-36 |
| SSBP1 | 0.527 | 1.45E-39 | FAM210A | 0.422 | 1.85E-24 | TNS1 | -0.508 | 2.15E-36 |
| IGF2BP3 | 0.527 | 1.73E-39 | ACTR6 | 0.421 | 2.17E-24 | GGTLC1 | -0.508 | 1.63E-36 |
| DCUN1D5 | 0.527 | 1.51E-39 | POLD3 | 0.421 | 2.27E-24 | SCGB3A2 | -0.508 | 2.26E-36 |
| NUP205 | 0.527 | 1.65E-39 | PUS7 | 0.421 | 2.04E-24 | TEF | -0.509 | 1.31E-36 |
| BOLA3 | 0.527 | 1.32E-39 | POFUT1 | 0.421 | 2.05E-24 | SCN7A | -0.512 | 3.71E-37 |
| MARS | 0.527 | 1.48E-39 | KNOP1 | 0.421 | 1.95E-24 | VIPR1 | -0.513 | 2.93E-37 |
| TARS | 0.526 | 1.96E-39 | GARS | 0.421 | 2.36E-24 | SFTA3 | -0.513 | 3.19E-37 |
| LRRC42 | 0.525 | 3.55E-39 | TAF5 | 0.421 | 2.39E-24 | GPD1L | -0.516 | 1.02E-37 |
| TMEM106C | 0.525 | 3.14E-39 | SCNM1 | 0.421 | 2.40E-24 | HOPX | -0.521 | 1.32E-38 |
| RRM1 | 0.525 | 3.70E-39 | UTP11 | 0.421 | 1.91E-24 | ADH1B | -0.527 | 1.26E-39 |
| CCDC59 | 0.524 | 4.68E-39 | CCDC167 | 0.421 | 1.92E-24 | MALL | -0.528 | 9.41E-40 |
| PSMC4 | 0.523 | 5.66E-39 | ATP5F1B | 0.42 | 2.63E-24 | SNX30 | -0.529 | 6.87E-40 |
| ZC3HC1 | 0.523 | 7.35E-39 | SNRPC | 0.42 | 3.04E-24 | SLC22A3 | -0.531 | 3.09E-40 |
| HBS1L | 0.523 | 7.18E-39 | PSMB7 | 0.42 | 2.88E-24 | SFTPB | -0.532 | 2.29E-40 |
| TBRG4 | 0.523 | 6.31E-39 | TMEM69 | 0.42 | 2.50E-24 | TMPRSS2 | -0.533 | 1.53E-40 |
| PNO1 | 0.522 | 1.05E-38 | MRPL52 | 0.42 | 2.76E-24 | DPYSL2 | -0.536 | 4.32E-41 |
| TIMM8A | 0.522 | 1.20E-38 | PSMD1 | 0.42 | 3.21E-24 | RFTN1 | -0.536 | 4.32E-41 |
| EIF4A3 | 0.522 | 9.09E-39 | ALKBH2 | 0.42 | 3.16E-24 | ITGA9 | -0.537 | 2.28E-41 |
| ARL6IP6 | 0.522 | 9.49E-39 | GSS | 0.419 | 3.39E-24 | BTBD9 | -0.537 | 2.97E-41 |
| PPM1G | 0.521 | 1.58E-38 | ACLY | 0.419 | 3.56E-24 | TNS2 | -0.539 | 1.10E-41 |
| CCT4 | 0.521 | 1.64E-38 | DDX18 | 0.418 | 5.18E-24 | NFIX | -0.542 | 4.14E-42 |
| POLE | 0.521 | 1.45E-38 | NOP2 | 0.418 | 4.91E-24 | DLC1 | -0.542 | 3.38E-42 |
| PFDN4 | 0.52 | 2.59E-38 | EXOSC9 | 0.418 | 4.67E-24 | SUSD2 | -0.543 | 2.39E-42 |
| EXOSC2 | 0.52 | 2.59E-38 | TOMM70 | 0.417 | 7.09E-24 | RHOBTB2 | -0.544 | 1.74E-42 |
| METTL5 | 0.52 | 2.50E-38 | TEDC1 | 0.417 | 5.65E-24 | INMT | -0.545 | 1.22E-42 |
| HYLS1 | 0.52 | 2.17E-38 | DHFR | 0.417 | 5.80E-24 | ZBTB4 | -0.546 | 6.60E-43 |
| REXO5 | 0.519 | 2.79E-38 | POLD1 | 0.416 | 9.02E-24 | TLR5 | -0.546 | 5.92E-43 |
| UTP18 | 0.519 | 3.32E-38 | SF3B6 | 0.416 | 7.58E-24 | ST3GAL5 | -0.548 | 3.16E-43 |
| TBP | 0.519 | 2.90E-38 | YEATS4 | 0.416 | 9.58E-24 | TMEM163 | -0.555 | 1.67E-44 |
| SNRPG | 0.519 | 3.33E-38 | YARS2 | 0.416 | 9.38E-24 | CFAP221 | -0.555 | 1.21E-44 |
| PFN2 | 0.518 | 4.14E-38 | RMI1 | 0.416 | 8.86E-24 | NR3C2 | -0.557 | 6.44E-45 |
| HSPE1 | 0.518 | 4.98E-38 | MAGEA3 | 0.416 | 9.29E-24 | SNED1 | -0.558 | 4.89E-45 |
| LSM5 | 0.517 | 7.03E-38 | EPOP | 0.416 | 7.75E-24 | NAPSA | -0.561 | 1.09E-45 |
| CCDC34 | 0.517 | 6.46E-38 | DDX52 | 0.416 | 8.84E-24 | ADGRF5 | -0.568 | 4.68E-47 |
| SLC2A1 | 0.517 | 5.76E-38 | RALA | 0.415 | 1.08E-23 | CACNA2D2 | -0.576 | 1.56E-48 |
| PA2G4 | 0.517 | 6.18E-38 | PSMA4 | 0.415 | 1.00E-23 | SELENBP1 | -0.58 | 2.16E-49 |
| CYCS | 0.517 | 7.00E-38 | DNTTIP2 | 0.415 | 1.10E-23 | CYP4B1 | -0.583 | 5.72E-50 |
| MRPL3 | 0.516 | 1.14E-37 | ANAPC5 | 0.415 | 1.25E-23 | C1orf116 | -0.585 | 1.60E-50 |
| CCNE2 | 0.516 | 1.10E-37 | ERH | 0.415 | 1.04E-23 | CRY2 | -0.628 | 3.92E-60 |
| PSMB1 | 0.514 | 1.84E-37 | EMC9 | 0.415 | 1.17E-23 | C16orf89 | -0.655 | 7.35E-67 |
| MRPL37 | 0.514 | 1.90E-37 | RBM28 | 0.415 | 1.16E-23 |  |  |  |
