# Supplementary material for: Evaluation of clinical value and potential mechanism of MTFR2 in lung adenocarcinoma via bioinformatics
Source: BMC Cancer. 2021 May 26;21:619. doi: 10.1186/s12885-021-08378-3 (PMC8157440; doi:10.1186/s12885-021-08378-3)
Supplement: Supplementary file 2 — Additional file 2: Table S2. GO analysis of MTFR2 co-expressed genes. [file 12885_2021_8378_MOESM2_ESM.docx]

Table S2. GO analysis of MTFR2 co-expressed genes.

| Ontology | ID | Description | Count | *p* value | *p* adjust |
| --- | --- | --- | --- | --- | --- |
| BP | GO:0006260 | DNA replication | 93 | 6.60E-49 | 3.04E-45 |
| BP | GO:0007059 | chromosome segregation | 84 | 3.37E-38 | 7.76E-35 |
| BP | GO:0006261 | DNA-dependent DNA replication | 56 | 7.47E-35 | 1.15E-31 |
| BP | GO:0000280 | nuclear division | 89 | 7.86E-33 | 9.05E-30 |
| BP | GO:0140014 | mitotic nuclear division | 72 | 1.11E-32 | 1.02E-29 |
| BP | GO:0048285 | organelle fission | 93 | 3.65E-32 | 2.80E-29 |
| BP | GO:0000819 | sister chromatid segregation | 58 | 1.10E-31 | 7.24E-29 |
| BP | GO:0098813 | nuclear chromosome segregation | 68 | 1.97E-31 | 1.13E-28 |
| BP | GO:0000070 | mitotic sister chromatid segregation | 53 | 3.51E-31 | 1.79E-28 |
| BP | GO:1901990 | regulation of mitotic cell cycle phase transition | 94 | 5.65E-30 | 2.60E-27 |
| BP | GO:1901987 | regulation of cell cycle phase transition | 97 | 4.13E-29 | 1.73E-26 |
| BP | GO:0044839 | cell cycle G2/M phase transition | 68 | 6.22E-27 | 2.39E-24 |
| BP | GO:0000086 | G2/M transition of mitotic cell cycle | 65 | 1.21E-26 | 4.28E-24 |
| BP | GO:1902749 | regulation of cell cycle G2/M phase transition | 56 | 1.84E-23 | 6.05E-21 |
| BP | GO:0010389 | regulation of G2/M transition of mitotic cell cycle | 53 | 5.28E-23 | 1.62E-20 |
| BP | GO:0045930 | negative regulation of mitotic cell cycle | 69 | 9.39E-23 | 2.70E-20 |
| BP | GO:0044843 | cell cycle G1/S phase transition | 66 | 2.19E-22 | 5.92E-20 |
| BP | GO:0000082 | G1/S transition of mitotic cell cycle | 63 | 5.96E-22 | 1.53E-19 |
| BP | GO:0007051 | spindle organization | 46 | 6.90E-22 | 1.67E-19 |
| BP | GO:0010948 | negative regulation of cell cycle process | 72 | 7.45E-22 | 1.72E-19 |
| BP | GO:0000075 | cell cycle checkpoint | 53 | 1.32E-21 | 2.88E-19 |
| BP | GO:0044786 | cell cycle DNA replication | 29 | 3.31E-21 | 6.92E-19 |
| BP | GO:1901988 | negative regulation of cell cycle phase transition | 60 | 3.67E-21 | 7.35E-19 |
| BP | GO:1901991 | negative regulation of mitotic cell cycle phase transition | 57 | 9.33E-21 | 1.72E-18 |
| BP | GO:0051052 | regulation of DNA metabolic process | 77 | 9.35E-21 | 1.72E-18 |
| BP | GO:0000226 | microtubule cytoskeleton organization | 83 | 1.23E-20 | 2.18E-18 |
| BP | GO:0033260 | nuclear DNA replication | 26 | 2.24E-20 | 3.82E-18 |
| BP | GO:0007093 | mitotic cell cycle checkpoint | 44 | 3.87E-20 | 6.24E-18 |
| BP | GO:0031145 | anaphase-promoting complex-dependent catabolic process | 33 | 3.93E-20 | 6.24E-18 |
| BP | GO:0034502 | protein localization to chromosome | 33 | 6.22E-20 | 9.55E-18 |
| BP | GO:1902850 | microtubule cytoskeleton organization involved in mitosis | 38 | 4.40E-19 | 6.54E-17 |
| BP | GO:0051983 | regulation of chromosome segregation | 34 | 6.27E-19 | 9.03E-17 |
| BP | GO:0006403 | RNA localization | 48 | 9.63E-17 | 1.34E-14 |
| BP | GO:0007052 | mitotic spindle organization | 32 | 1.10E-16 | 1.49E-14 |
| BP | GO:0071103 | DNA conformation change | 56 | 2.14E-16 | 2.82E-14 |
| BP | GO:0000723 | telomere maintenance | 40 | 4.76E-16 | 6.08E-14 |
| BP | GO:0006310 | DNA recombination | 51 | 8.42E-16 | 1.05E-13 |
| BP | GO:0007088 | regulation of mitotic nuclear division | 39 | 1.07E-15 | 1.30E-13 |
| BP | GO:0006270 | DNA replication initiation | 18 | 2.28E-15 | 2.69E-13 |
| BP | GO:0006275 | regulation of DNA replication | 31 | 8.60E-15 | 9.90E-13 |
| BP | GO:0032200 | telomere organization | 40 | 9.39E-15 | 1.05E-12 |
| BP | GO:0071897 | DNA biosynthetic process | 43 | 1.21E-14 | 1.33E-12 |
| BP | GO:1902750 | negative regulation of cell cycle G2/M phase transition | 30 | 1.38E-14 | 1.47E-12 |
| BP | GO:0072331 | signal transduction by p53 class mediator | 51 | 2.40E-14 | 2.51E-12 |
| BP | GO:0031570 | DNA integrity checkpoint | 36 | 2.49E-14 | 2.55E-12 |
| BP | GO:0007091 | metaphase/anaphase transition of mitotic cell cycle | 21 | 2.82E-14 | 2.77E-12 |
| BP | GO:0010965 | regulation of mitotic sister chromatid separation | 21 | 2.82E-14 | 2.77E-12 |
| BP | GO:0051054 | positive regulation of DNA metabolic process | 46 | 3.11E-14 | 2.99E-12 |
| BP | GO:0090329 | regulation of DNA-dependent DNA replication | 21 | 4.75E-14 | 4.46E-12 |
| BP | GO:0033045 | regulation of sister chromatid segregation | 25 | 5.25E-14 | 4.84E-12 |
| BP | GO:0044784 | metaphase/anaphase transition of cell cycle | 21 | 7.85E-14 | 6.95E-12 |
| BP | GO:0051306 | mitotic sister chromatid separation | 21 | 7.85E-14 | 6.95E-12 |
| BP | GO:0090068 | positive regulation of cell cycle process | 52 | 1.09E-13 | 9.45E-12 |
| BP | GO:0032201 | telomere maintenance via semi-conservative replication | 16 | 1.11E-13 | 9.45E-12 |
| BP | GO:0051783 | regulation of nuclear division | 39 | 1.37E-13 | 1.14E-11 |
| BP | GO:0010972 | negative regulation of G2/M transition of mitotic cell cycle | 27 | 1.39E-13 | 1.14E-11 |
| BP | GO:1905818 | regulation of chromosome separation | 21 | 3.26E-13 | 2.64E-11 |
| BP | GO:0071459 | protein localization to chromosome, centromeric region | 14 | 3.75E-13 | 2.98E-11 |
| BP | GO:0033044 | regulation of chromosome organization | 54 | 3.99E-13 | 3.11E-11 |
| BP | GO:0000725 | recombinational repair | 30 | 4.29E-13 | 3.29E-11 |
| BP | GO:0045787 | positive regulation of cell cycle | 60 | 6.14E-13 | 4.64E-11 |
| BP | GO:0030071 | regulation of mitotic metaphase/anaphase transition | 19 | 1.03E-12 | 7.64E-11 |
| BP | GO:0006302 | double-strand break repair | 42 | 1.69E-12 | 1.23E-10 |
| BP | GO:0000724 | double-strand break repair via homologous recombination | 29 | 2.08E-12 | 1.48E-10 |
| BP | GO:0034501 | protein localization to kinetochore | 12 | 2.09E-12 | 1.48E-10 |
| BP | GO:1902099 | regulation of metaphase/anaphase transition of cell cycle | 19 | 2.71E-12 | 1.88E-10 |
| BP | GO:0033047 | regulation of mitotic sister chromatid segregation | 21 | 2.74E-12 | 1.88E-10 |
| BP | GO:0006323 | DNA packaging | 39 | 7.65E-12 | 5.18E-10 |
| BP | GO:0051321 | meiotic cell cycle | 40 | 8.01E-12 | 5.34E-10 |
| BP | GO:0034660 | ncRNA metabolic process | 67 | 8.39E-12 | 5.52E-10 |
| BP | GO:0008608 | attachment of spindle microtubules to kinetochore | 15 | 9.75E-12 | 6.32E-10 |
| BP | GO:0051310 | metaphase plate congression | 20 | 1.20E-11 | 7.70E-10 |
| BP | GO:0022613 | ribonucleoprotein complex biogenesis | 58 | 1.53E-11 | 9.67E-10 |
| BP | GO:0051304 | chromosome separation | 22 | 1.68E-11 | 1.04E-09 |
| BP | GO:0000077 | DNA damage checkpoint | 31 | 2.05E-11 | 1.26E-09 |
| BP | GO:0051169 | nuclear transport | 50 | 2.65E-11 | 1.61E-09 |
| BP | GO:1904874 | positive regulation of telomerase RNA localization to Cajal body | 11 | 2.73E-11 | 1.63E-09 |
| BP | GO:0051225 | spindle assembly | 25 | 3.07E-11 | 1.82E-09 |
| BP | GO:0006336 | DNA replication-independent nucleosome assembly | 19 | 5.26E-11 | 3.03E-09 |
| BP | GO:0034724 | DNA replication-independent nucleosome organization | 19 | 5.26E-11 | 3.03E-09 |
| BP | GO:0050000 | chromosome localization | 22 | 5.99E-11 | 3.30E-09 |
| BP | GO:0051303 | establishment of chromosome localization | 22 | 5.99E-11 | 3.30E-09 |
| BP | GO:0045841 | negative regulation of mitotic metaphase/anaphase transition | 15 | 6.02E-11 | 3.30E-09 |
| BP | GO:2000816 | negative regulation of mitotic sister chromatid separation | 15 | 6.02E-11 | 3.30E-09 |
| BP | GO:0006913 | nucleocytoplasmic transport | 49 | 6.11E-11 | 3.31E-09 |
| BP | GO:0033046 | negative regulation of sister chromatid segregation | 16 | 6.29E-11 | 3.37E-09 |
| BP | GO:1903405 | protein localization to nuclear body | 9 | 7.06E-11 | 3.65E-09 |
| BP | GO:1904851 | positive regulation of establishment of protein localization to telomere | 9 | 7.06E-11 | 3.65E-09 |
| BP | GO:1904867 | protein localization to Cajal body | 9 | 7.06E-11 | 3.65E-09 |
| BP | GO:0034508 | centromere complex assembly | 19 | 7.68E-11 | 3.93E-09 |
| BP | GO:0072401 | signal transduction involved in DNA integrity checkpoint | 22 | 8.11E-11 | 4.06E-09 |
| BP | GO:0072422 | signal transduction involved in DNA damage checkpoint | 22 | 8.11E-11 | 4.06E-09 |
| BP | GO:0070200 | establishment of protein localization to telomere | 11 | 8.26E-11 | 4.09E-09 |
| BP | GO:0051985 | negative regulation of chromosome segregation | 16 | 1.03E-10 | 5.01E-09 |
| BP | GO:1902100 | negative regulation of metaphase/anaphase transition of cell cycle | 15 | 1.05E-10 | 5.01E-09 |
| BP | GO:1905819 | negative regulation of chromosome separation | 15 | 1.05E-10 | 5.01E-09 |
| BP | GO:0072395 | signal transduction involved in cell cycle checkpoint | 22 | 1.09E-10 | 5.18E-09 |
| BP | GO:0050657 | nucleic acid transport | 35 | 1.24E-10 | 5.78E-09 |
| BP | GO:0050658 | RNA transport | 35 | 1.24E-10 | 5.78E-09 |
| BP | GO:1903046 | meiotic cell cycle process | 32 | 1.39E-10 | 6.41E-09 |
| BP | GO:0000083 | regulation of transcription involved in G1/S transition of mitotic cell cycle | 14 | 1.61E-10 | 7.34E-09 |
| BP | GO:0044774 | mitotic DNA integrity checkpoint | 24 | 1.94E-10 | 8.76E-09 |
| BP | GO:0051236 | establishment of RNA localization | 35 | 2.02E-10 | 8.96E-09 |
| BP | GO:1901796 | regulation of signal transduction by p53 class mediator | 35 | 2.02E-10 | 8.96E-09 |
| BP | GO:0045839 | negative regulation of mitotic nuclear division | 17 | 2.09E-10 | 9.17E-09 |
| BP | GO:0006405 | RNA export from nucleus | 28 | 2.82E-10 | 1.22E-08 |
| BP | GO:0033048 | negative regulation of mitotic sister chromatid segregation | 15 | 2.93E-10 | 1.26E-08 |
| BP | GO:0031055 | chromatin remodeling at centromere | 17 | 3.14E-10 | 1.34E-08 |
| BP | GO:0070203 | regulation of establishment of protein localization to telomere | 9 | 3.68E-10 | 1.55E-08 |
| BP | GO:0044773 | mitotic DNA damage checkpoint | 23 | 4.34E-10 | 1.82E-08 |
| BP | GO:1904872 | regulation of telomerase RNA localization to Cajal body | 11 | 5.40E-10 | 2.24E-08 |
| BP | GO:0140013 | meiotic nuclear division | 30 | 5.66E-10 | 2.33E-08 |
| BP | GO:0034080 | CENP-A containing nucleosome assembly | 16 | 6.17E-10 | 2.49E-08 |
| BP | GO:0061641 | CENP-A containing chromatin organization | 16 | 6.17E-10 | 2.49E-08 |
| BP | GO:0009124 | nucleoside monophosphate biosynthetic process | 36 | 6.89E-10 | 2.76E-08 |
| BP | GO:0007098 | centrosome cycle | 26 | 9.34E-10 | 3.71E-08 |
| BP | GO:0043161 | proteasome-mediated ubiquitin-dependent protein catabolic process | 49 | 1.14E-09 | 4.48E-08 |
| BP | GO:0090670 | RNA localization to Cajal body | 11 | 1.22E-09 | 4.62E-08 |
| BP | GO:0090671 | telomerase RNA localization to Cajal body | 11 | 1.22E-09 | 4.62E-08 |
| BP | GO:0090672 | telomerase RNA localization | 11 | 1.22E-09 | 4.62E-08 |
| BP | GO:0090685 | RNA localization to nucleus | 11 | 1.22E-09 | 4.62E-08 |
| BP | GO:0065004 | protein-DNA complex assembly | 37 | 1.34E-09 | 5.06E-08 |
| BP | GO:0070202 | regulation of establishment of protein localization to chromosome | 9 | 1.40E-09 | 5.14E-08 |
| BP | GO:1904816 | positive regulation of protein localization to chromosome, telomeric region | 9 | 1.40E-09 | 5.14E-08 |
| BP | GO:1990173 | protein localization to nucleoplasm | 9 | 1.40E-09 | 5.14E-08 |
| BP | GO:0019882 | antigen processing and presentation | 38 | 1.44E-09 | 5.26E-08 |
| BP | GO:0043044 | ATP-dependent chromatin remodeling | 21 | 1.68E-09 | 6.10E-08 |
| BP | GO:0070199 | establishment of protein localization to chromosome | 12 | 2.01E-09 | 7.23E-08 |
| BP | GO:0071426 | ribonucleoprotein complex export from nucleus | 26 | 2.09E-09 | 7.46E-08 |
| BP | GO:0071166 | ribonucleoprotein complex localization | 26 | 2.54E-09 | 8.99E-08 |
| BP | GO:0051383 | kinetochore organization | 11 | 2.56E-09 | 8.99E-08 |
| BP | GO:0032392 | DNA geometric change | 22 | 2.60E-09 | 9.06E-08 |
| BP | GO:0051784 | negative regulation of nuclear division | 17 | 2.82E-09 | 9.77E-08 |
| BP | GO:1903311 | regulation of mRNA metabolic process | 43 | 2.93E-09 | 1.01E-07 |
| BP | GO:2000573 | positive regulation of DNA biosynthetic process | 19 | 3.71E-09 | 1.27E-07 |
| BP | GO:0007094 | mitotic spindle assembly checkpoint | 13 | 3.89E-09 | 1.29E-07 |
| BP | GO:0031577 | spindle checkpoint | 13 | 3.89E-09 | 1.29E-07 |
| BP | GO:0071173 | spindle assembly checkpoint | 13 | 3.89E-09 | 1.29E-07 |
| BP | GO:0071174 | mitotic spindle checkpoint | 13 | 3.89E-09 | 1.29E-07 |
| BP | GO:0007080 | mitotic metaphase plate congression | 15 | 4.11E-09 | 1.35E-07 |
| BP | GO:0043487 | regulation of RNA stability | 31 | 4.40E-09 | 1.44E-07 |
| BP | GO:0042254 | ribosome biogenesis | 35 | 5.86E-09 | 1.89E-07 |
| BP | GO:0002478 | antigen processing and presentation of exogenous peptide antigen | 32 | 5.88E-09 | 1.89E-07 |
| BP | GO:0031023 | microtubule organizing center organization | 26 | 6.51E-09 | 2.08E-07 |
| BP | GO:0140053 | mitochondrial gene expression | 29 | 6.81E-09 | 2.16E-07 |
| BP | GO:0042770 | signal transduction in response to DNA damage | 27 | 7.20E-09 | 2.27E-07 |
| BP | GO:0043486 | histone exchange | 17 | 7.43E-09 | 2.33E-07 |
| BP | GO:0019884 | antigen processing and presentation of exogenous antigen | 32 | 7.86E-09 | 2.43E-07 |
| BP | GO:0051168 | nuclear export | 32 | 7.86E-09 | 2.43E-07 |
| BP | GO:0032508 | DNA duplex unwinding | 20 | 8.06E-09 | 2.47E-07 |
| BP | GO:0043488 | regulation of mRNA stability | 30 | 8.11E-09 | 2.47E-07 |
| BP | GO:0031146 | SCF-dependent proteasomal ubiquitin-dependent protein catabolic process | 19 | 8.40E-09 | 2.54E-07 |
| BP | GO:0061418 | regulation of transcription from RNA polymerase II promoter in response to hypoxia | 20 | 1.03E-08 | 3.10E-07 |
| BP | GO:0061013 | regulation of mRNA catabolic process | 32 | 1.04E-08 | 3.12E-07 |
| BP | GO:0032543 | mitochondrial translation | 26 | 1.12E-08 | 3.31E-07 |
| BP | GO:0048002 | antigen processing and presentation of peptide antigen | 33 | 1.14E-08 | 3.35E-07 |
| BP | GO:1904814 | regulation of protein localization to chromosome, telomeric region | 9 | 1.14E-08 | 3.35E-07 |
| BP | GO:0006409 | tRNA export from nucleus | 13 | 1.58E-08 | 4.54E-07 |
| BP | GO:0051031 | tRNA transport | 13 | 1.58E-08 | 4.54E-07 |
| BP | GO:0071431 | tRNA-containing ribonucleoprotein complex export from nucleus | 13 | 1.58E-08 | 4.54E-07 |
| BP | GO:0071824 | protein-DNA complex subunit organization | 38 | 1.72E-08 | 4.91E-07 |
| BP | GO:0015931 | nucleobase-containing compound transport | 36 | 1.91E-08 | 5.44E-07 |
| BP | GO:0006338 | chromatin remodeling | 29 | 2.35E-08 | 6.64E-07 |
| BP | GO:0072413 | signal transduction involved in mitotic cell cycle checkpoint | 17 | 2.42E-08 | 6.72E-07 |
| BP | GO:1902402 | signal transduction involved in mitotic DNA damage checkpoint | 17 | 2.42E-08 | 6.72E-07 |
| BP | GO:1902403 | signal transduction involved in mitotic DNA integrity checkpoint | 17 | 2.42E-08 | 6.72E-07 |
| BP | GO:0010498 | proteasomal protein catabolic process | 51 | 2.52E-08 | 6.95E-07 |
| BP | GO:2000278 | regulation of DNA biosynthetic process | 23 | 3.37E-08 | 9.25E-07 |
| BP | GO:0006301 | postreplication repair | 15 | 3.50E-08 | 9.54E-07 |
| BP | GO:0045740 | positive regulation of DNA replication | 13 | 3.65E-08 | 9.87E-07 |
| BP | GO:1901293 | nucleoside phosphate biosynthetic process | 49 | 3.97E-08 | 1.07E-06 |
| BP | GO:0006521 | regulation of cellular amino acid metabolic process | 17 | 4.19E-08 | 1.12E-06 |
| BP | GO:2001251 | negative regulation of chromosome organization | 25 | 4.84E-08 | 1.29E-06 |
| BP | GO:0097064 | ncRNA export from nucleus | 13 | 5.40E-08 | 1.43E-06 |
| BP | GO:0071456 | cellular response to hypoxia | 34 | 5.51E-08 | 1.45E-06 |
| BP | GO:0051302 | regulation of cell division | 29 | 5.59E-08 | 1.46E-06 |
| BP | GO:0043618 | regulation of transcription from RNA polymerase II promoter in response to stress | 23 | 5.85E-08 | 1.52E-06 |
| BP | GO:0022616 | DNA strand elongation | 9 | 5.87E-08 | 1.52E-06 |
| BP | GO:0016072 | rRNA metabolic process | 32 | 5.95E-08 | 1.53E-06 |
| BP | GO:0034404 | nucleobase-containing small molecule biosynthetic process | 34 | 6.21E-08 | 1.59E-06 |
| BP | GO:0006611 | protein export from nucleus | 29 | 6.43E-08 | 1.64E-06 |
| BP | GO:0045931 | positive regulation of mitotic cell cycle | 28 | 6.70E-08 | 1.70E-06 |
| BP | GO:0009165 | nucleotide biosynthetic process | 48 | 6.76E-08 | 1.70E-06 |
| BP | GO:0031571 | mitotic G1 DNA damage checkpoint | 17 | 7.09E-08 | 1.76E-06 |
| BP | GO:0044819 | mitotic G1/S transition checkpoint | 17 | 7.09E-08 | 1.76E-06 |
| BP | GO:0006977 | DNA damage response, signal transduction by p53 class mediator resulting in cell cycle arrest | 16 | 7.12E-08 | 1.76E-06 |
| BP | GO:0044783 | G1 DNA damage checkpoint | 17 | 9.13E-08 | 2.25E-06 |
| BP | GO:0072431 | signal transduction involved in mitotic G1 DNA damage checkpoint | 16 | 9.37E-08 | 2.28E-06 |
| BP | GO:1902400 | intracellular signal transduction involved in G1 DNA damage checkpoint | 16 | 9.37E-08 | 2.28E-06 |
| BP | GO:1902036 | regulation of hematopoietic stem cell differentiation | 18 | 1.06E-07 | 2.57E-06 |
| BP | GO:0030261 | chromosome condensation | 12 | 1.09E-07 | 2.62E-06 |
| BP | GO:0031503 | protein-containing complex localization | 36 | 1.33E-07 | 3.19E-06 |
| BP | GO:0009156 | ribonucleoside monophosphate biosynthetic process | 31 | 1.38E-07 | 3.30E-06 |
| BP | GO:0070316 | regulation of G0 to G1 transition | 14 | 1.44E-07 | 3.42E-06 |
| BP | GO:0006520 | cellular amino acid metabolic process | 46 | 1.50E-07 | 3.55E-06 |
| BP | GO:0034470 | ncRNA processing | 41 | 1.64E-07 | 3.84E-06 |
| BP | GO:0070507 | regulation of microtubule cytoskeleton organization | 29 | 1.65E-07 | 3.84E-06 |
| BP | GO:0043620 | regulation of DNA-templated transcription in response to stress | 23 | 1.65E-07 | 3.84E-06 |
| BP | GO:0061640 | cytoskeleton-dependent cytokinesis | 19 | 1.74E-07 | 4.02E-06 |
| BP | GO:0036294 | cellular response to decreased oxygen levels | 34 | 1.75E-07 | 4.03E-06 |
| BP | GO:0009123 | nucleoside monophosphate metabolic process | 46 | 1.78E-07 | 4.09E-06 |
| BP | GO:0070646 | protein modification by small protein removal | 39 | 1.90E-07 | 4.33E-06 |
| BP | GO:0002479 | antigen processing and presentation of exogenous peptide antigen via MHC class I, TAP-dependent | 18 | 2.07E-07 | 4.70E-06 |
| BP | GO:0070198 | protein localization to chromosome, telomeric region | 11 | 2.12E-07 | 4.79E-06 |
| BP | GO:0031497 | chromatin assembly | 27 | 2.16E-07 | 4.84E-06 |
| BP | GO:0006334 | nucleosome assembly | 25 | 2.32E-07 | 5.20E-06 |
| BP | GO:0006297 | nucleotide-excision repair, DNA gap filling | 10 | 2.45E-07 | 5.44E-06 |
| BP | GO:0000018 | regulation of DNA recombination | 19 | 2.61E-07 | 5.77E-06 |
| BP | GO:0045023 | G0 to G1 transition | 14 | 2.64E-07 | 5.82E-06 |
| BP | GO:0009127 | purine nucleoside monophosphate biosynthetic process | 29 | 2.75E-07 | 6.01E-06 |
| BP | GO:0009168 | purine ribonucleoside monophosphate biosynthetic process | 29 | 2.75E-07 | 6.01E-06 |
| BP | GO:0070498 | interleukin-1-mediated signaling pathway | 21 | 2.85E-07 | 6.18E-06 |
| BP | GO:0000281 | mitotic cytokinesis | 17 | 3.00E-07 | 6.46E-06 |
| BP | GO:0000377 | RNA splicing, via transesterification reactions with bulged adenosine as nucleophile | 45 | 3.01E-07 | 6.46E-06 |
| BP | GO:0000398 | mRNA splicing, via spliceosome | 45 | 3.01E-07 | 6.46E-06 |
| BP | GO:0070317 | negative regulation of G0 to G1 transition | 13 | 3.14E-07 | 6.69E-06 |
| BP | GO:0009308 | amine metabolic process | 24 | 3.18E-07 | 6.71E-06 |
| BP | GO:1901532 | regulation of hematopoietic progenitor cell differentiation | 19 | 3.18E-07 | 6.71E-06 |
| BP | GO:0009262 | deoxyribonucleotide metabolic process | 11 | 3.24E-07 | 6.81E-06 |
| BP | GO:0006401 | RNA catabolic process | 47 | 3.36E-07 | 7.04E-06 |
| BP | GO:0000910 | cytokinesis | 26 | 3.44E-07 | 7.17E-06 |
| BP | GO:0071453 | cellular response to oxygen levels | 35 | 3.57E-07 | 7.41E-06 |
| BP | GO:0042590 | antigen processing and presentation of exogenous peptide antigen via MHC class I | 18 | 3.90E-07 | 8.05E-06 |
| BP | GO:0000375 | RNA splicing, via transesterification reactions | 45 | 4.20E-07 | 8.63E-06 |
| BP | GO:0009141 | nucleoside triphosphate metabolic process | 44 | 4.30E-07 | 8.80E-06 |
| BP | GO:0006333 | chromatin assembly or disassembly | 29 | 4.51E-07 | 9.20E-06 |
| BP | GO:2001252 | positive regulation of chromosome organization | 28 | 4.99E-07 | 1.01E-05 |
| BP | GO:0006399 | tRNA metabolic process | 27 | 5.46E-07 | 1.10E-05 |
| BP | GO:0070126 | mitochondrial translational termination | 19 | 5.64E-07 | 1.13E-05 |
| BP | GO:0007076 | mitotic chromosome condensation | 8 | 5.73E-07 | 1.15E-05 |
| BP | GO:0033238 | regulation of cellular amine metabolic process | 18 | 5.83E-07 | 1.16E-05 |
| BP | GO:0009116 | nucleoside metabolic process | 22 | 5.84E-07 | 1.16E-05 |
| BP | GO:0000076 | DNA replication checkpoint | 7 | 5.97E-07 | 1.17E-05 |
| BP | GO:2000105 | positive regulation of DNA-dependent DNA replication | 7 | 5.97E-07 | 1.17E-05 |
| BP | GO:0007062 | sister chromatid cohesion | 14 | 6.15E-07 | 1.20E-05 |
| BP | GO:0036297 | interstrand cross-link repair | 14 | 6.15E-07 | 1.20E-05 |
| BP | GO:1901989 | positive regulation of cell cycle phase transition | 20 | 6.27E-07 | 1.22E-05 |
| BP | GO:0006406 | mRNA export from nucleus | 21 | 7.84E-07 | 1.51E-05 |
| BP | GO:0071427 | mRNA-containing ribonucleoprotein complex export from nucleus | 21 | 7.84E-07 | 1.51E-05 |
| BP | GO:0006364 | rRNA processing | 26 | 8.73E-07 | 1.68E-05 |
| BP | GO:0006284 | base-excision repair | 12 | 9.41E-07 | 1.80E-05 |
| BP | GO:0060218 | hematopoietic stem cell differentiation | 18 | 1.04E-06 | 1.97E-05 |
| BP | GO:0090307 | mitotic spindle assembly | 14 | 1.04E-06 | 1.97E-05 |
| BP | GO:0006415 | translational termination | 20 | 1.04E-06 | 1.97E-05 |
| BP | GO:0051028 | mRNA transport | 25 | 1.08E-06 | 2.02E-05 |
| BP | GO:0016579 | protein deubiquitination | 36 | 1.19E-06 | 2.23E-05 |
| BP | GO:0051988 | regulation of attachment of spindle microtubules to kinetochore | 7 | 1.36E-06 | 2.54E-05 |
| BP | GO:0006298 | mismatch repair | 10 | 1.46E-06 | 2.69E-05 |
| BP | GO:1902751 | positive regulation of cell cycle G2/M phase transition | 10 | 1.46E-06 | 2.69E-05 |
| BP | GO:0044106 | cellular amine metabolic process | 22 | 1.68E-06 | 3.09E-05 |
| BP | GO:0006282 | regulation of DNA repair | 21 | 1.71E-06 | 3.14E-05 |
| BP | GO:0019985 | translesion synthesis | 12 | 1.74E-06 | 3.18E-05 |
| BP | GO:0070125 | mitochondrial translational elongation | 18 | 1.80E-06 | 3.27E-05 |
| BP | GO:2001020 | regulation of response to DNA damage stimulus | 30 | 1.93E-06 | 3.49E-05 |
| BP | GO:0002474 | antigen processing and presentation of peptide antigen via MHC class I | 19 | 1.93E-06 | 3.49E-05 |
| BP | GO:0032212 | positive regulation of telomere maintenance via telomerase | 11 | 2.05E-06 | 3.68E-05 |
| BP | GO:1901657 | glycosyl compound metabolic process | 23 | 2.11E-06 | 3.78E-05 |
| BP | GO:0009163 | nucleoside biosynthetic process | 12 | 2.33E-06 | 4.16E-05 |
| BP | GO:0006414 | translational elongation | 22 | 2.56E-06 | 4.56E-05 |
| BP | GO:0072522 | purine-containing compound biosynthetic process | 38 | 2.59E-06 | 4.59E-05 |
| BP | GO:0030330 | DNA damage response, signal transduction by p53 class mediator | 20 | 2.72E-06 | 4.79E-05 |
| BP | GO:1901659 | glycosyl compound biosynthetic process | 12 | 3.09E-06 | 5.44E-05 |
| BP | GO:0009142 | nucleoside triphosphate biosynthetic process | 27 | 3.27E-06 | 5.73E-05 |
| BP | GO:0034728 | nucleosome organization | 26 | 3.32E-06 | 5.79E-05 |
| BP | GO:0051782 | negative regulation of cell division | 8 | 3.33E-06 | 5.79E-05 |
| BP | GO:0075733 | intracellular transport of virus | 14 | 3.44E-06 | 5.96E-05 |
| BP | GO:0044766 | multi-organism transport | 16 | 3.54E-06 | 6.07E-05 |
| BP | GO:1902579 | multi-organism localization | 16 | 3.54E-06 | 6.07E-05 |
| BP | GO:0018205 | peptidyl-lysine modification | 45 | 3.55E-06 | 6.07E-05 |
| BP | GO:0060249 | anatomical structure homeostasis | 48 | 3.85E-06 | 6.56E-05 |
| BP | GO:0000731 | DNA synthesis involved in DNA repair | 13 | 3.87E-06 | 6.58E-05 |
| BP | GO:0006397 | mRNA processing | 55 | 3.94E-06 | 6.66E-05 |
| BP | GO:0071158 | positive regulation of cell cycle arrest | 17 | 3.97E-06 | 6.66E-05 |
| BP | GO:1901992 | positive regulation of mitotic cell cycle phase transition | 17 | 3.97E-06 | 6.66E-05 |
| BP | GO:0001666 | response to hypoxia | 43 | 3.98E-06 | 6.66E-05 |
| BP | GO:1900182 | positive regulation of protein localization to nucleus | 16 | 4.27E-06 | 7.13E-05 |
| BP | GO:0010971 | positive regulation of G2/M transition of mitotic cell cycle | 9 | 4.38E-06 | 7.29E-05 |
| BP | GO:0007143 | female meiotic nuclear division | 10 | 4.42E-06 | 7.32E-05 |
| BP | GO:0032886 | regulation of microtubule-based process | 29 | 4.62E-06 | 7.62E-05 |
| BP | GO:0032465 | regulation of cytokinesis | 17 | 4.73E-06 | 7.78E-05 |
| BP | GO:1904358 | positive regulation of telomere maintenance via telomere lengthening | 11 | 5.21E-06 | 8.53E-05 |
| BP | GO:0046653 | tetrahydrofolate metabolic process | 8 | 5.46E-06 | 8.92E-05 |
| BP | GO:0009260 | ribonucleotide biosynthetic process | 36 | 6.21E-06 | 0.000101109 |
| BP | GO:0006188 | IMP biosynthetic process | 6 | 6.59E-06 | 0.000106137 |
| BP | GO:0046040 | IMP metabolic process | 6 | 6.59E-06 | 0.000106137 |
| BP | GO:0046794 | transport of virus | 14 | 6.59E-06 | 0.000106137 |
| BP | GO:0042769 | DNA damage response, detection of DNA damage | 11 | 6.94E-06 | 0.000111391 |
| BP | GO:0032210 | regulation of telomere maintenance via telomerase | 13 | 7.80E-06 | 0.000124669 |
| BP | GO:0000966 | RNA 5'-end processing | 8 | 8.64E-06 | 0.000137196 |
| BP | GO:0070482 | response to oxygen levels | 45 | 8.66E-06 | 0.000137196 |
| BP | GO:0036293 | response to decreased oxygen levels | 43 | 8.67E-06 | 0.000137196 |
| BP | GO:0046390 | ribose phosphate biosynthetic process | 36 | 9.29E-06 | 0.000146471 |
| BP | GO:0009200 | deoxyribonucleoside triphosphate metabolic process | 7 | 9.47E-06 | 0.000148764 |
| BP | GO:0009161 | ribonucleoside monophosphate metabolic process | 40 | 9.69E-06 | 0.000151699 |
| BP | GO:0010833 | telomere maintenance via telomere lengthening | 16 | 1.04E-05 | 0.000162549 |
| BP | GO:0006457 | protein folding | 26 | 1.10E-05 | 0.000171851 |
| BP | GO:0045005 | DNA-dependent DNA replication maintenance of fidelity | 10 | 1.16E-05 | 0.000179812 |
| BP | GO:0006760 | folic acid-containing compound metabolic process | 9 | 1.34E-05 | 0.000206532 |
| BP | GO:0009394 | 2'-deoxyribonucleotide metabolic process | 9 | 1.34E-05 | 0.000206532 |
| BP | GO:2000779 | regulation of double-strand break repair | 15 | 1.37E-05 | 0.000209832 |
| BP | GO:0071156 | regulation of cell cycle arrest | 19 | 1.38E-05 | 0.000211104 |
| BP | GO:0002244 | hematopoietic progenitor cell differentiation | 24 | 1.48E-05 | 0.000225751 |
| BP | GO:0051653 | spindle localization | 11 | 1.55E-05 | 0.00023581 |
| BP | GO:0090175 | regulation of establishment of planar polarity | 19 | 1.58E-05 | 0.000239547 |
| BP | GO:0006402 | mRNA catabolic process | 40 | 1.59E-05 | 0.000239714 |
| BP | GO:0051382 | kinetochore assembly | 7 | 1.60E-05 | 0.000239714 |
| BP | GO:0099116 | tRNA 5'-end processing | 7 | 1.60E-05 | 0.000239714 |
| BP | GO:0034504 | protein localization to nucleus | 30 | 1.60E-05 | 0.000239899 |
| BP | GO:0006164 | purine nucleotide biosynthetic process | 35 | 1.67E-05 | 0.000248535 |
| BP | GO:0010639 | negative regulation of organelle organization | 41 | 1.83E-05 | 0.000271684 |
| BP | GO:0040001 | establishment of mitotic spindle localization | 9 | 1.87E-05 | 0.000277559 |
| BP | GO:0009167 | purine ribonucleoside monophosphate metabolic process | 38 | 1.93E-05 | 0.000283739 |
| BP | GO:0006278 | RNA-dependent DNA biosynthetic process | 15 | 1.93E-05 | 0.000283739 |
| BP | GO:1902807 | negative regulation of cell cycle G1/S phase transition | 21 | 1.93E-05 | 0.000283739 |
| BP | GO:1904666 | regulation of ubiquitin protein ligase activity | 8 | 1.97E-05 | 0.000288635 |
| BP | GO:0009126 | purine nucleoside monophosphate metabolic process | 38 | 2.07E-05 | 0.000301608 |
| BP | GO:0006289 | nucleotide-excision repair | 18 | 2.21E-05 | 0.000320932 |
| BP | GO:0043624 | cellular protein complex disassembly | 27 | 2.50E-05 | 0.000361881 |
| BP | GO:0007004 | telomere maintenance via telomerase | 14 | 2.54E-05 | 0.000365525 |
| BP | GO:0045132 | meiotic chromosome segregation | 14 | 2.54E-05 | 0.000365525 |
| BP | GO:0009144 | purine nucleoside triphosphate metabolic process | 38 | 2.55E-05 | 0.000366532 |
| BP | GO:0019692 | deoxyribose phosphate metabolic process | 9 | 2.58E-05 | 0.000367576 |
| BP | GO:0031297 | replication fork processing | 9 | 2.58E-05 | 0.000367576 |
| BP | GO:0046112 | nucleobase biosynthetic process | 6 | 2.62E-05 | 0.000372266 |
| BP | GO:0002223 | stimulatory C-type lectin receptor signaling pathway | 19 | 2.68E-05 | 0.000379373 |
| BP | GO:0032206 | positive regulation of telomere maintenance | 12 | 2.73E-05 | 0.000385953 |
| BP | GO:0043687 | post-translational protein modification | 41 | 3.10E-05 | 0.000435268 |
| BP | GO:2000134 | negative regulation of G1/S transition of mitotic cell cycle | 20 | 3.10E-05 | 0.000435268 |
| BP | GO:0051656 | establishment of organelle localization | 48 | 3.34E-05 | 0.000468009 |
| BP | GO:0009152 | purine ribonucleotide biosynthetic process | 33 | 3.36E-05 | 0.000468349 |
| BP | GO:0090263 | positive regulation of canonical Wnt signaling pathway | 21 | 3.46E-05 | 0.000481925 |
| BP | GO:0031572 | G2 DNA damage checkpoint | 9 | 3.49E-05 | 0.000484612 |
| BP | GO:0002220 | innate immune response activating cell surface receptor signaling pathway | 19 | 3.90E-05 | 0.000536695 |
| BP | GO:2000736 | regulation of stem cell differentiation | 19 | 3.90E-05 | 0.000536695 |
| BP | GO:0090305 | nucleic acid phosphodiester bond hydrolysis | 33 | 3.90E-05 | 0.000536695 |
| BP | GO:1904356 | regulation of telomere maintenance via telomere lengthening | 13 | 3.94E-05 | 0.00054047 |
| BP | GO:0034471 | ncRNA 5'-end processing | 7 | 4.01E-05 | 0.000546368 |
| BP | GO:0042276 | error-prone translesion synthesis | 7 | 4.01E-05 | 0.000546368 |
| BP | GO:0051984 | positive regulation of chromosome segregation | 8 | 4.09E-05 | 0.000555682 |
| BP | GO:0031123 | RNA 3'-end processing | 18 | 4.30E-05 | 0.000580277 |
| BP | GO:0060071 | Wnt signaling pathway, planar cell polarity pathway | 18 | 4.30E-05 | 0.000580277 |
| BP | GO:0009411 | response to UV | 21 | 4.33E-05 | 0.000582905 |
| BP | GO:0008380 | RNA splicing | 47 | 4.34E-05 | 0.000582905 |
| BP | GO:1900180 | regulation of protein localization to nucleus | 19 | 4.41E-05 | 0.000589975 |
| BP | GO:0022411 | cellular component disassembly | 51 | 4.61E-05 | 0.000614953 |
| BP | GO:0006369 | termination of RNA polymerase II transcription | 9 | 4.67E-05 | 0.00062157 |
| BP | GO:0031124 | mRNA 3'-end processing | 16 | 4.93E-05 | 0.000653937 |
| BP | GO:0046605 | regulation of centrosome cycle | 12 | 5.02E-05 | 0.000664593 |
| BP | GO:0070987 | error-free translesion synthesis | 7 | 6.03E-05 | 0.000795793 |
| BP | GO:0061982 | meiosis I cell cycle process | 16 | 6.52E-05 | 0.000857591 |
| BP | GO:1902806 | regulation of cell cycle G1/S phase transition | 26 | 6.71E-05 | 0.000880877 |
| BP | GO:0006283 | transcription-coupled nucleotide-excision repair | 14 | 6.93E-05 | 0.000906065 |
| BP | GO:0007050 | cell cycle arrest | 29 | 7.95E-05 | 0.001035976 |
| BP | GO:0009201 | ribonucleoside triphosphate biosynthetic process | 23 | 7.96E-05 | 0.001035976 |
| BP | GO:0042558 | pteridine-containing compound metabolic process | 9 | 8.04E-05 | 0.001040098 |
| BP | GO:0043094 | cellular metabolic compound salvage | 9 | 8.04E-05 | 0.001040098 |
| BP | GO:1901292 | nucleoside phosphate catabolic process | 24 | 8.06E-05 | 0.001040219 |
| BP | GO:0001736 | establishment of planar polarity | 19 | 8.87E-05 | 0.001137525 |
| BP | GO:0007164 | establishment of tissue polarity | 19 | 8.87E-05 | 0.001137525 |
| BP | GO:0010569 | regulation of double-strand break repair via homologous recombination | 10 | 9.18E-05 | 0.00117392 |
| BP | GO:0006732 | coenzyme metabolic process | 40 | 9.63E-05 | 0.001228305 |
| BP | GO:2000045 | regulation of G1/S transition of mitotic cell cycle | 24 | 9.67E-05 | 0.001229727 |
| BP | GO:0031109 | microtubule polymerization or depolymerization | 16 | 9.75E-05 | 0.001236598 |
| BP | GO:0071347 | cellular response to interleukin-1 | 22 | 0.000103874 | 0.001314118 |
| BP | GO:0009112 | nucleobase metabolic process | 8 | 0.000105605 | 0.001328718 |
| BP | GO:0015949 | nucleobase-containing small molecule interconversion | 8 | 0.000105605 | 0.001328718 |
| BP | GO:0072527 | pyrimidine-containing compound metabolic process | 16 | 0.000110988 | 0.001388863 |
| BP | GO:0097711 | ciliary basal body-plasma membrane docking | 16 | 0.000110988 | 0.001388863 |
| BP | GO:0009199 | ribonucleoside triphosphate metabolic process | 36 | 0.000111326 | 0.001389307 |
| BP | GO:0000387 | spliceosomal snRNP assembly | 11 | 0.000113293 | 0.001410034 |
| BP | GO:0006575 | cellular modified amino acid metabolic process | 24 | 0.000115477 | 0.001433348 |
| BP | GO:0016570 | histone modification | 44 | 0.000118013 | 0.001460893 |
| BP | GO:0016569 | covalent chromatin modification | 45 | 0.000119427 | 0.001474421 |
| BP | GO:0046931 | pore complex assembly | 6 | 0.000121922 | 0.001501199 |
| BP | GO:0051298 | centrosome duplication | 12 | 0.000125263 | 0.001538224 |
| BP | GO:0050852 | T cell receptor signaling pathway | 24 | 0.00012605 | 0.001542703 |
| BP | GO:0009166 | nucleotide catabolic process | 23 | 0.000126632 | 0.001542703 |
| BP | GO:0090090 | negative regulation of canonical Wnt signaling pathway | 23 | 0.000126632 | 0.001542703 |
| BP | GO:0009162 | deoxyribonucleoside monophosphate metabolic process | 5 | 0.000130575 | 0.00157821 |
| BP | GO:0009263 | deoxyribonucleotide biosynthetic process | 5 | 0.000130575 | 0.00157821 |
| BP | GO:0051231 | spindle elongation | 5 | 0.000130575 | 0.00157821 |
| BP | GO:0032984 | protein-containing complex disassembly | 34 | 0.000138277 | 0.001665878 |
| BP | GO:0030177 | positive regulation of Wnt signaling pathway | 23 | 0.000138552 | 0.001665878 |
| BP | GO:0071168 | protein localization to chromatin | 8 | 0.000140349 | 0.001683091 |
| BP | GO:0016925 | protein sumoylation | 14 | 0.000146204 | 0.001748747 |
| BP | GO:0006353 | DNA-templated transcription, termination | 13 | 0.000150631 | 0.001797041 |
| BP | GO:0007127 | meiosis I | 15 | 0.000156879 | 0.001866737 |
| BP | GO:0000209 | protein polyubiquitination | 33 | 0.000158943 | 0.001886424 |
| BP | GO:0001738 | morphogenesis of a polarized epithelium | 20 | 0.000159793 | 0.00189164 |
| BP | GO:0072528 | pyrimidine-containing compound biosynthetic process | 11 | 0.000164643 | 0.001944058 |
| BP | GO:0051293 | establishment of spindle localization | 9 | 0.000167621 | 0.00197229 |
| BP | GO:0032204 | regulation of telomere maintenance | 14 | 0.000168319 | 0.00197229 |
| BP | GO:1900034 | regulation of cellular response to heat | 14 | 0.000168319 | 0.00197229 |
| BP | GO:0006165 | nucleoside diphosphate phosphorylation | 19 | 0.000169287 | 0.001978595 |
| BP | GO:0042398 | cellular modified amino acid biosynthetic process | 10 | 0.000173233 | 0.002019584 |
| BP | GO:0001824 | blastocyst development | 16 | 0.000182761 | 0.002125293 |
| BP | GO:0046134 | pyrimidine nucleoside biosynthetic process | 8 | 0.000183993 | 0.002134231 |
| BP | GO:0043174 | nucleoside salvage | 6 | 0.000185486 | 0.002146136 |
| BP | GO:0046939 | nucleotide phosphorylation | 19 | 0.000207751 | 0.002397732 |
| BP | GO:0006296 | nucleotide-excision repair, DNA incision, 5'-to lesion | 9 | 0.000210163 | 0.002419501 |
| BP | GO:0022618 | ribonucleoprotein complex assembly | 26 | 0.00021119 | 0.002419732 |
| BP | GO:0043039 | tRNA aminoacylation | 10 | 0.000211234 | 0.002419732 |
| BP | GO:0071826 | ribonucleoprotein complex subunit organization | 27 | 0.000217206 | 0.002481969 |
| BP | GO:0001682 | tRNA 5'-leader removal | 5 | 0.000227907 | 0.002585009 |
| BP | GO:0007100 | mitotic centrosome separation | 5 | 0.000227907 | 0.002585009 |
| BP | GO:0016446 | somatic hypermutation of immunoglobulin genes | 5 | 0.000227907 | 0.002585009 |
| BP | GO:0044818 | mitotic G2/M transition checkpoint | 7 | 0.00023896 | 0.002703707 |
| BP | GO:0043038 | amino acid activation | 10 | 0.000255989 | 0.002889293 |
| BP | GO:0009314 | response to radiation | 44 | 0.000258477 | 0.002910238 |
| BP | GO:0010212 | response to ionizing radiation | 20 | 0.000259198 | 0.002911238 |
| BP | GO:0016572 | histone phosphorylation | 9 | 0.000261301 | 0.002927716 |
| BP | GO:0009148 | pyrimidine nucleoside triphosphate biosynthetic process | 6 | 0.000272586 | 0.003046742 |
| BP | GO:0016445 | somatic diversification of immunoglobulins | 11 | 0.000277915 | 0.003098788 |
| BP | GO:0000079 | regulation of cyclin-dependent protein serine/threonine kinase activity | 14 | 0.000288108 | 0.003204679 |
| BP | GO:0051188 | cofactor biosynthetic process | 34 | 0.000292578 | 0.003246557 |
| BP | GO:1901976 | regulation of cell cycle checkpoint | 8 | 0.000304802 | 0.003374066 |
| BP | GO:0002204 | somatic recombination of immunoglobulin genes involved in immune response | 10 | 0.000308414 | 0.003381544 |
| BP | GO:0002208 | somatic diversification of immunoglobulins involved in immune response | 10 | 0.000308414 | 0.003381544 |
| BP | GO:0045190 | isotype switching | 10 | 0.000308414 | 0.003381544 |
| BP | GO:0051438 | regulation of ubiquitin-protein transferase activity | 10 | 0.000308414 | 0.003381544 |
| BP | GO:0033683 | nucleotide-excision repair, DNA incision | 9 | 0.000322329 | 0.003525713 |
| BP | GO:0031100 | animal organ regeneration | 13 | 0.000357042 | 0.003896158 |
| BP | GO:0009206 | purine ribonucleoside triphosphate biosynthetic process | 21 | 0.000370515 | 0.003993038 |
| BP | GO:0009205 | purine ribonucleoside triphosphate metabolic process | 34 | 0.00037085 | 0.003993038 |
| BP | GO:0000212 | meiotic spindle organization | 5 | 0.00037199 | 0.003993038 |
| BP | GO:0002566 | somatic diversification of immune receptors via somatic mutation | 5 | 0.00037199 | 0.003993038 |
| BP | GO:0007077 | mitotic nuclear envelope disassembly | 5 | 0.00037199 | 0.003993038 |
| BP | GO:0051299 | centrosome separation | 5 | 0.00037199 | 0.003993038 |
| BP | GO:1904668 | positive regulation of ubiquitin protein ligase activity | 5 | 0.00037199 | 0.003993038 |
| BP | GO:0042772 | DNA damage response, signal transduction resulting in transcription | 6 | 0.000388827 | 0.00416407 |
| BP | GO:0009145 | purine nucleoside triphosphate biosynthetic process | 21 | 0.000403626 | 0.004312519 |
| BP | GO:0070555 | response to interleukin-1 | 23 | 0.000412515 | 0.004397299 |
| BP | GO:0034605 | cellular response to heat | 17 | 0.000430165 | 0.004574852 |
| BP | GO:0002200 | somatic diversification of immune receptors | 12 | 0.000436903 | 0.004635805 |
| BP | GO:0016447 | somatic recombination of immunoglobulin gene segments | 10 | 0.00044035 | 0.004661633 |
| BP | GO:0006754 | ATP biosynthetic process | 20 | 0.000446706 | 0.00471808 |
| BP | GO:1904029 | regulation of cyclin-dependent protein kinase activity | 14 | 0.0004746 | 0.005001224 |
| BP | GO:0090224 | regulation of spindle organization | 9 | 0.000479853 | 0.00504503 |
| BP | GO:0042455 | ribonucleoside biosynthetic process | 8 | 0.000483599 | 0.005072829 |
| BP | GO:0006220 | pyrimidine nucleotide metabolic process | 11 | 0.000525357 | 0.005498337 |
| BP | GO:0030178 | negative regulation of Wnt signaling pathway | 24 | 0.000533197 | 0.00556774 |
| BP | GO:0051053 | negative regulation of DNA metabolic process | 19 | 0.00053752 | 0.005587536 |
| BP | GO:0140056 | organelle localization by membrane tethering | 19 | 0.00053752 | 0.005587536 |
| BP | GO:0009147 | pyrimidine nucleoside triphosphate metabolic process | 6 | 0.00054045 | 0.005592751 |
| BP | GO:0046655 | folic acid metabolic process | 6 | 0.00054045 | 0.005592751 |
| BP | GO:0001833 | inner cell mass cell proliferation | 5 | 0.000575581 | 0.005929648 |
| BP | GO:0042559 | pteridine-containing compound biosynthetic process | 5 | 0.000575581 | 0.005929648 |
| BP | GO:0009132 | nucleoside diphosphate metabolic process | 20 | 0.000578401 | 0.005945392 |
| BP | GO:0009119 | ribonucleoside metabolic process | 14 | 0.000601167 | 0.006165642 |
| BP | GO:0002562 | somatic diversification of immune receptors via germline recombination within a single locus | 11 | 0.000609982 | 0.006228311 |
| BP | GO:0016444 | somatic cell DNA recombination | 11 | 0.000609982 | 0.006228311 |
| BP | GO:0006213 | pyrimidine nucleoside metabolic process | 10 | 0.00061606 | 0.006276454 |
| BP | GO:0006096 | glycolytic process | 16 | 0.000619903 | 0.006287782 |
| BP | GO:1903578 | regulation of ATP metabolic process | 16 | 0.000619903 | 0.006287782 |
| BP | GO:0046434 | organophosphate catabolic process | 26 | 0.000623429 | 0.006309647 |
| BP | GO:0046031 | ADP metabolic process | 17 | 0.000638927 | 0.006452324 |
| BP | GO:0006757 | ATP generation from ADP | 16 | 0.000685981 | 0.006912349 |
| BP | GO:0007339 | binding of sperm to zona pellucida | 8 | 0.000739136 | 0.00741551 |
| BP | GO:1901998 | toxin transport | 8 | 0.000739136 | 0.00741551 |
| BP | GO:0035567 | non-canonical Wnt signaling pathway | 19 | 0.000761956 | 0.007627839 |
| BP | GO:0072425 | signal transduction involved in G2 DNA damage checkpoint | 5 | 0.000852605 | 0.008461738 |
| BP | GO:0090231 | regulation of spindle checkpoint | 5 | 0.000852605 | 0.008461738 |
| BP | GO:0090266 | regulation of mitotic cell cycle spindle assembly checkpoint | 5 | 0.000852605 | 0.008461738 |
| BP | GO:1903504 | regulation of mitotic spindle checkpoint | 5 | 0.000852605 | 0.008461738 |
| BP | GO:0051443 | positive regulation of ubiquitin-protein transferase activity | 7 | 0.000893806 | 0.008851564 |
| BP | GO:0008156 | negative regulation of DNA replication | 8 | 0.000902425 | 0.008898649 |
| BP | GO:0051973 | positive regulation of telomerase activity | 8 | 0.000902425 | 0.008898649 |
| BP | GO:0042866 | pyruvate biosynthetic process | 16 | 0.00092142 | 0.009066537 |
| BP | GO:0051170 | import into nucleus | 17 | 0.000929114 | 0.009122748 |
| BP | GO:0007568 | aging | 33 | 0.000943017 | 0.009239561 |
| BP | GO:2000104 | negative regulation of DNA-dependent DNA replication | 6 | 0.000977785 | 0.009559874 |
| BP | GO:0051781 | positive regulation of cell division | 13 | 0.001088103 | 0.010564048 |
| BP | GO:0062012 | regulation of small molecule metabolic process | 42 | 0.001088419 | 0.010564048 |
| BP | GO:0032467 | positive regulation of cytokinesis | 8 | 0.001093394 | 0.010564048 |
| BP | GO:0032506 | cytokinetic process | 8 | 0.001093394 | 0.010564048 |
| BP | GO:0060236 | regulation of mitotic spindle organization | 8 | 0.001093394 | 0.010564048 |
| BP | GO:0008033 | tRNA processing | 15 | 0.001094279 | 0.010564048 |
| BP | GO:0009108 | coenzyme biosynthetic process | 27 | 0.00109655 | 0.010564048 |
| BP | GO:0002381 | immunoglobulin production involved in immunoglobulin mediated immune response | 10 | 0.001142099 | 0.01097989 |
| BP | GO:0022406 | membrane docking | 19 | 0.001151972 | 0.011051727 |
| BP | GO:0006144 | purine nucleobase metabolic process | 5 | 0.001217947 | 0.011636194 |
| BP | GO:0043101 | purine-containing compound salvage | 5 | 0.001217947 | 0.011636194 |
| BP | GO:0006110 | regulation of glycolytic process | 12 | 0.00124119 | 0.011833706 |
| BP | GO:0009303 | rRNA transcription | 7 | 0.001387926 | 0.013205372 |
| BP | GO:0046034 | ATP metabolic process | 30 | 0.001403006 | 0.013321329 |
| BP | GO:0019886 | antigen processing and presentation of exogenous peptide antigen via MHC class II | 14 | 0.001431088 | 0.013554343 |
| BP | GO:0048863 | stem cell differentiation | 26 | 0.001433434 | 0.013554343 |
| BP | GO:0009150 | purine ribonucleotide metabolic process | 46 | 0.001453258 | 0.013713636 |
| BP | GO:0030811 | regulation of nucleotide catabolic process | 12 | 0.001569459 | 0.014779877 |
| BP | GO:0042278 | purine nucleoside metabolic process | 11 | 0.001577738 | 0.014827517 |
| BP | GO:0002377 | immunoglobulin production | 14 | 0.00158214 | 0.014838609 |
| BP | GO:0051972 | regulation of telomerase activity | 9 | 0.001593499 | 0.014914765 |
| BP | GO:0060828 | regulation of canonical Wnt signaling pathway | 28 | 0.00160389 | 0.014981564 |
| BP | GO:0000729 | DNA double-strand break processing | 6 | 0.001645854 | 0.01528056 |
| BP | GO:0006458 | 'de novo' protein folding | 6 | 0.001645854 | 0.01528056 |
| BP | GO:0007064 | mitotic sister chromatid cohesion | 6 | 0.001645854 | 0.01528056 |
| BP | GO:0008340 | determination of adult lifespan | 5 | 0.001687247 | 0.015508531 |
| BP | GO:0009219 | pyrimidine deoxyribonucleotide metabolic process | 5 | 0.001687247 | 0.015508531 |
| BP | GO:0030397 | membrane disassembly | 5 | 0.001687247 | 0.015508531 |
| BP | GO:0033262 | regulation of nuclear cell cycle DNA replication | 5 | 0.001687247 | 0.015508531 |
| BP | GO:0051081 | nuclear envelope disassembly | 5 | 0.001687247 | 0.015508531 |
| BP | GO:0007099 | centriole replication | 7 | 0.001703837 | 0.01559875 |
| BP | GO:0045910 | negative regulation of DNA recombination | 7 | 0.001703837 | 0.01559875 |
| BP | GO:0031398 | positive regulation of protein ubiquitination | 15 | 0.001764252 | 0.016119806 |
| BP | GO:0043467 | regulation of generation of precursor metabolites and energy | 18 | 0.001783821 | 0.016266328 |
| BP | GO:0051255 | spindle midzone assembly | 4 | 0.001807709 | 0.016419128 |
| BP | GO:0051315 | attachment of mitotic spindle microtubules to kinetochore | 4 | 0.001807709 | 0.016419128 |
| BP | GO:1903829 | positive regulation of cellular protein localization | 32 | 0.00184364 | 0.016712524 |
| BP | GO:0009135 | purine nucleoside diphosphate metabolic process | 17 | 0.001854292 | 0.016743164 |
| BP | GO:0009179 | purine ribonucleoside diphosphate metabolic process | 17 | 0.001854292 | 0.016743164 |
| BP | GO:0010824 | regulation of centrosome duplication | 8 | 0.00186575 | 0.016813657 |
| BP | GO:0002495 | antigen processing and presentation of peptide antigen via MHC class II | 14 | 0.001924224 | 0.017306745 |
| BP | GO:2001021 | negative regulation of response to DNA damage stimulus | 12 | 0.001966079 | 0.017648723 |
| BP | GO:1905330 | regulation of morphogenesis of an epithelium | 21 | 0.002001353 | 0.017930409 |
| BP | GO:0051196 | regulation of coenzyme metabolic process | 13 | 0.002072027 | 0.018527542 |
| BP | GO:0000132 | establishment of mitotic spindle orientation | 6 | 0.002087635 | 0.018594893 |
| BP | GO:0008334 | histone mRNA metabolic process | 6 | 0.002087635 | 0.018594893 |
| BP | GO:0002504 | antigen processing and presentation of peptide or polysaccharide antigen via MHC class II | 14 | 0.002116991 | 0.018819967 |
| BP | GO:0009408 | response to heat | 19 | 0.002126136 | 0.018864851 |
| BP | GO:0009185 | ribonucleoside diphosphate metabolic process | 17 | 0.002179578 | 0.019301836 |
| BP | GO:0002639 | positive regulation of immunoglobulin production | 8 | 0.002201725 | 0.019460544 |
| BP | GO:0006978 | DNA damage response, signal transduction by p53 class mediator resulting in transcription of p21 class mediator | 5 | 0.00227669 | 0.020007931 |
| BP | GO:0009264 | deoxyribonucleotide catabolic process | 5 | 0.00227669 | 0.020007931 |
| BP | GO:0034629 | cellular protein-containing complex localization | 5 | 0.00227669 | 0.020007931 |
| BP | GO:0050821 | protein stabilization | 20 | 0.002299028 | 0.020165761 |
| BP | GO:0002312 | B cell activation involved in immune response | 11 | 0.002544273 | 0.022274478 |
| BP | GO:0006221 | pyrimidine nucleotide biosynthetic process | 8 | 0.002583409 | 0.022531432 |
| BP | GO:0006418 | tRNA aminoacylation for protein translation | 8 | 0.002583409 | 0.022531432 |
| BP | GO:0006730 | one-carbon metabolic process | 6 | 0.002613326 | 0.022706349 |
| BP | GO:0045830 | positive regulation of isotype switching | 6 | 0.002613326 | 0.022706349 |
| BP | GO:0008655 | pyrimidine-containing compound salvage | 4 | 0.002710452 | 0.023286626 |
| BP | GO:0009143 | nucleoside triphosphate catabolic process | 4 | 0.002710452 | 0.023286626 |
| BP | GO:0009151 | purine deoxyribonucleotide metabolic process | 4 | 0.002710452 | 0.023286626 |
| BP | GO:0035404 | histone-serine phosphorylation | 4 | 0.002710452 | 0.023286626 |
| BP | GO:0043097 | pyrimidine nucleoside salvage | 4 | 0.002710452 | 0.023286626 |
| BP | GO:0048875 | chemical homeostasis within a tissue | 4 | 0.002710452 | 0.023286626 |
| BP | GO:0098781 | ncRNA transcription | 14 | 0.002793302 | 0.023953736 |
| BP | GO:0070192 | chromosome organization involved in meiotic cell cycle | 9 | 0.002838857 | 0.02429914 |
| BP | GO:0050851 | antigen receptor-mediated signaling pathway | 24 | 0.002921397 | 0.024959246 |
| BP | GO:0007569 | cell aging | 15 | 0.002988872 | 0.025480022 |
| BP | GO:0007019 | microtubule depolymerization | 7 | 0.002993418 | 0.025480022 |
| BP | GO:0006312 | mitotic recombination | 5 | 0.003002791 | 0.025512639 |
| BP | GO:0002821 | positive regulation of adaptive immune response | 13 | 0.003066948 | 0.026009749 |
| BP | GO:1900542 | regulation of purine nucleotide metabolic process | 16 | 0.003123913 | 0.026444157 |
| BP | GO:0006767 | water-soluble vitamin metabolic process | 12 | 0.003325694 | 0.028049124 |
| BP | GO:0043470 | regulation of carbohydrate catabolic process | 12 | 0.003325694 | 0.028049124 |
| BP | GO:0046394 | carboxylic acid biosynthetic process | 39 | 0.003497099 | 0.029440847 |
| BP | GO:0098534 | centriole assembly | 7 | 0.003556989 | 0.029890392 |
| BP | GO:0016053 | organic acid biosynthetic process | 39 | 0.003639608 | 0.030528951 |
| BP | GO:0016571 | histone methylation | 16 | 0.003650295 | 0.030556509 |
| BP | GO:0002218 | activation of innate immune response | 31 | 0.003656164 | 0.030556509 |
| BP | GO:0010565 | regulation of cellular ketone metabolic process | 20 | 0.003667814 | 0.030598342 |
| BP | GO:0046128 | purine ribonucleoside metabolic process | 10 | 0.003687753 | 0.030709044 |
| BP | GO:0006999 | nuclear pore organization | 4 | 0.003879683 | 0.032038567 |
| BP | GO:0051095 | regulation of helicase activity | 4 | 0.003879683 | 0.032038567 |
| BP | GO:0001832 | blastocyst growth | 5 | 0.003882198 | 0.032038567 |
| BP | GO:0007095 | mitotic G2 DNA damage checkpoint | 5 | 0.003882198 | 0.032038567 |
| BP | GO:0046386 | deoxyribose phosphate catabolic process | 5 | 0.003882198 | 0.032038567 |
| BP | GO:0038061 | NIK/NF-kappaB signaling | 20 | 0.003909809 | 0.03220871 |
| BP | GO:0042273 | ribosomal large subunit biogenesis | 6 | 0.003954317 | 0.032517197 |
| BP | GO:0033209 | tumor necrosis factor-mediated signaling pathway | 19 | 0.003972387 | 0.032607559 |
| BP | GO:0006733 | oxidoreduction coenzyme metabolic process | 21 | 0.004063891 | 0.033299322 |
| BP | GO:0035690 | cellular response to drug | 34 | 0.004142892 | 0.033886352 |
| BP | GO:0007548 | sex differentiation | 27 | 0.004156657 | 0.033938666 |
| BP | GO:0045911 | positive regulation of DNA recombination | 7 | 0.004197746 | 0.034213486 |
| BP | GO:0006140 | regulation of nucleotide metabolic process | 16 | 0.004247485 | 0.034496771 |
| BP | GO:0007292 | female gamete generation | 16 | 0.004247485 | 0.034496771 |
| BP | GO:0034968 | histone lysine methylation | 14 | 0.004308178 | 0.034928098 |
| BP | GO:0006890 | retrograde vesicle-mediated transport, Golgi to ER | 11 | 0.004371483 | 0.035379052 |
| BP | GO:0006479 | protein methylation | 19 | 0.00452575 | 0.036499262 |
| BP | GO:0008213 | protein alkylation | 19 | 0.00452575 | 0.036499262 |
| BP | GO:0072525 | pyridine-containing compound biosynthetic process | 17 | 0.00460601 | 0.0370816 |
| BP | GO:0002758 | innate immune response-activating signal transduction | 29 | 0.004736017 | 0.038061707 |
| BP | GO:0051294 | establishment of spindle orientation | 6 | 0.004789002 | 0.038420477 |
| BP | GO:0034644 | cellular response to UV | 11 | 0.004838487 | 0.038702197 |
| BP | GO:0007006 | mitochondrial membrane organization | 15 | 0.004840926 | 0.038702197 |
| BP | GO:0002714 | positive regulation of B cell mediated immunity | 7 | 0.004922095 | 0.039147228 |
| BP | GO:0002891 | positive regulation of immunoglobulin mediated immune response | 7 | 0.004922095 | 0.039147228 |
| BP | GO:0051567 | histone H3-K9 methylation | 7 | 0.004922095 | 0.039147228 |
| BP | GO:0031396 | regulation of protein ubiquitination | 21 | 0.00515792 | 0.040952109 |
| BP | GO:0042176 | regulation of protein catabolic process | 33 | 0.005245351 | 0.041574596 |
| BP | GO:0072698 | protein localization to microtubule cytoskeleton | 8 | 0.005325568 | 0.042080177 |
| BP | GO:0072524 | pyridine-containing compound metabolic process | 20 | 0.005327414 | 0.042080177 |
| BP | GO:0002921 | negative regulation of humoral immune response | 4 | 0.005348173 | 0.042171808 |
| BP | GO:0002824 | positive regulation of adaptive immune response based on somatic recombination of immune receptors built from immunoglobulin superfamily domains | 12 | 0.005360021 | 0.042192989 |
| BP | GO:0030111 | regulation of Wnt signaling pathway | 32 | 0.00536991 | 0.042198696 |
| BP | GO:0001701 | in utero embryonic development | 32 | 0.005604559 | 0.043967626 |
| BP | GO:0001825 | blastocyst formation | 7 | 0.005736535 | 0.044926436 |
| BP | GO:0071478 | cellular response to radiation | 19 | 0.00582379 | 0.045532351 |
| BP | GO:2001169 | regulation of ATP biosynthetic process | 12 | 0.005865867 | 0.045783587 |
| BP | GO:0045840 | positive regulation of mitotic nuclear division | 8 | 0.006071981 | 0.047232221 |
| BP | GO:0061647 | histone H3-K9 modification | 8 | 0.006071981 | 0.047232221 |
| BP | GO:0007063 | regulation of sister chromatid cohesion | 5 | 0.006167032 | 0.047729719 |
| BP | GO:0042451 | purine nucleoside biosynthetic process | 5 | 0.006167032 | 0.047729719 |
| BP | GO:0046129 | purine ribonucleoside biosynthetic process | 5 | 0.006167032 | 0.047729719 |
| BP | GO:0009988 | cell-cell recognition | 10 | 0.006375401 | 0.04909485 |
| BP | GO:0031110 | regulation of microtubule polymerization or depolymerization | 10 | 0.006375401 | 0.04909485 |
| BP | GO:1903312 | negative regulation of mRNA metabolic process | 10 | 0.006375401 | 0.04909485 |
| BP | GO:0051193 | regulation of cofactor metabolic process | 14 | 0.006429562 | 0.049429268 |
| CC | GO:0098687 | chromosomal region | 99 | 1.31E-43 | 7.44E-41 |
| CC | GO:0000775 | chromosome, centromeric region | 68 | 4.51E-37 | 1.28E-34 |
| CC | GO:0000793 | condensed chromosome | 70 | 1.09E-36 | 2.06E-34 |
| CC | GO:0000776 | kinetochore | 52 | 1.57E-30 | 2.23E-28 |
| CC | GO:0000779 | condensed chromosome, centromeric region | 46 | 2.47E-28 | 2.80E-26 |
| CC | GO:0000777 | condensed chromosome kinetochore | 43 | 3.58E-27 | 3.38E-25 |
| CC | GO:0005819 | spindle | 74 | 5.87E-25 | 4.76E-23 |
| CC | GO:0044454 | nuclear chromosome part | 81 | 3.29E-19 | 2.33E-17 |
| CC | GO:0000922 | spindle pole | 39 | 1.24E-15 | 7.82E-14 |
| CC | GO:0005813 | centrosome | 73 | 9.71E-15 | 5.50E-13 |
| CC | GO:0072686 | mitotic spindle | 27 | 3.74E-12 | 1.93E-10 |
| CC | GO:0005874 | microtubule | 56 | 6.98E-12 | 3.30E-10 |
| CC | GO:0000794 | condensed nuclear chromosome | 23 | 1.64E-11 | 7.16E-10 |
| CC | GO:0000940 | condensed chromosome outer kinetochore | 10 | 2.30E-11 | 9.30E-10 |
| CC | GO:0000781 | chromosome, telomeric region | 33 | 5.78E-11 | 2.19E-09 |
| CC | GO:0005657 | replication fork | 19 | 6.43E-11 | 2.28E-09 |
| CC | GO:0000785 | chromatin | 67 | 8.74E-11 | 2.92E-09 |
| CC | GO:0042555 | MCM complex | 9 | 3.35E-10 | 1.06E-08 |
| CC | GO:0000502 | proteasome complex | 19 | 7.07E-10 | 2.11E-08 |
| CC | GO:1905369 | endopeptidase complex | 19 | 9.65E-10 | 2.74E-08 |
| CC | GO:1905368 | peptidase complex | 20 | 2.41E-09 | 6.52E-08 |
| CC | GO:0030496 | midbody | 32 | 2.94E-09 | 7.57E-08 |
| CC | GO:0005876 | spindle microtubule | 17 | 3.36E-09 | 8.28E-08 |
| CC | GO:0000784 | nuclear chromosome, telomeric region | 25 | 2.00E-08 | 4.73E-07 |
| CC | GO:0101031 | chaperone complex | 10 | 2.21E-08 | 5.02E-07 |
| CC | GO:0000790 | nuclear chromatin | 43 | 7.27E-08 | 1.59E-06 |
| CC | GO:0005681 | spliceosomal complex | 28 | 1.25E-07 | 2.63E-06 |
| CC | GO:0005635 | nuclear envelope | 51 | 3.81E-07 | 7.72E-06 |
| CC | GO:0071007 | U2-type catalytic step 2 spliceosome | 11 | 4.36E-07 | 8.53E-06 |
| CC | GO:0034708 | methyltransferase complex | 19 | 4.81E-07 | 9.09E-06 |
| CC | GO:0005759 | mitochondrial matrix | 53 | 5.16E-07 | 9.37E-06 |
| CC | GO:0045120 | pronucleus | 8 | 5.29E-07 | 9.37E-06 |
| CC | GO:0000792 | heterochromatin | 17 | 9.27E-07 | 1.59E-05 |
| CC | GO:0034709 | methylosome | 7 | 1.27E-06 | 2.11E-05 |
| CC | GO:0034719 | SMN-Sm protein complex | 8 | 1.80E-06 | 2.84E-05 |
| CC | GO:0042599 | lamellar body | 8 | 1.80E-06 | 2.84E-05 |
| CC | GO:0022624 | proteasome accessory complex | 9 | 2.65E-06 | 4.06E-05 |
| CC | GO:0098798 | mitochondrial protein complex | 32 | 4.42E-06 | 6.22E-05 |
| CC | GO:0044215 | other organism | 16 | 4.50E-06 | 6.22E-05 |
| CC | GO:0044216 | other organism cell | 16 | 4.50E-06 | 6.22E-05 |
| CC | GO:0044217 | other organism part | 16 | 4.50E-06 | 6.22E-05 |
| CC | GO:0005643 | nuclear pore | 14 | 4.73E-06 | 6.29E-05 |
| CC | GO:0000313 | organellar ribosome | 17 | 4.88E-06 | 6.29E-05 |
| CC | GO:0005761 | mitochondrial ribosome | 17 | 4.88E-06 | 6.29E-05 |
| CC | GO:0031965 | nuclear membrane | 36 | 5.82E-06 | 7.33E-05 |
| CC | GO:0000152 | nuclear ubiquitin ligase complex | 11 | 6.28E-06 | 7.74E-05 |
| CC | GO:0018995 | host | 15 | 6.97E-06 | 8.23E-05 |
| CC | GO:0043657 | host cell | 15 | 6.97E-06 | 8.23E-05 |
| CC | GO:1904813 | ficolin-1-rich granule lumen | 21 | 7.82E-06 | 8.88E-05 |
| CC | GO:0005721 | pericentric heterochromatin | 8 | 7.99E-06 | 8.88E-05 |
| CC | GO:0005839 | proteasome core complex | 8 | 7.99E-06 | 8.88E-05 |
| CC | GO:0005732 | small nucleolar ribonucleoprotein complex | 9 | 8.65E-06 | 9.44E-05 |
| CC | GO:0005720 | nuclear heterochromatin | 10 | 1.06E-05 | 0.000113113 |
| CC | GO:0090734 | site of DNA damage | 14 | 1.57E-05 | 0.000164775 |
| CC | GO:0000307 | cyclin-dependent protein kinase holoenzyme complex | 9 | 2.37E-05 | 0.000239649 |
| CC | GO:0005684 | U2-type spliceosomal complex | 15 | 2.39E-05 | 0.000239649 |
| CC | GO:0000780 | condensed nuclear chromosome, centromeric region | 7 | 2.41E-05 | 0.000239649 |
| CC | GO:0019866 | organelle inner membrane | 51 | 3.01E-05 | 0.000294137 |
| CC | GO:0046540 | U4/U6 x U5 tri-snRNP complex | 10 | 3.22E-05 | 0.000304543 |
| CC | GO:0097526 | spliceosomal tri-snRNP complex | 10 | 3.22E-05 | 0.000304543 |
| CC | GO:0042575 | DNA polymerase complex | 6 | 4.36E-05 | 0.000404878 |
| CC | GO:0000315 | organellar large ribosomal subunit | 12 | 5.50E-05 | 0.000494873 |
| CC | GO:0005762 | mitochondrial large ribosomal subunit | 12 | 5.50E-05 | 0.000494873 |
| CC | GO:0071013 | catalytic step 2 spliceosome | 15 | 6.07E-05 | 0.000537527 |
| CC | GO:0010369 | chromocenter | 6 | 7.25E-05 | 0.000624232 |
| CC | GO:0097431 | mitotic spindle pole | 8 | 7.27E-05 | 0.000624232 |
| CC | GO:0030894 | replisome | 7 | 8.24E-05 | 0.000697556 |
| CC | GO:0051233 | spindle midzone | 8 | 9.81E-05 | 0.000817729 |
| CC | GO:0120114 | Sm-like protein family complex | 16 | 0.000111683 | 0.000917743 |
| CC | GO:0061695 | transferase complex, transferring phosphorus-containing groups | 24 | 0.000117356 | 0.000938459 |
| CC | GO:0005680 | anaphase-promoting complex | 7 | 0.000117514 | 0.000938459 |
| CC | GO:0035098 | ESC/E(Z) complex | 6 | 0.000174997 | 0.001373882 |
| CC | GO:0005743 | mitochondrial inner membrane | 45 | 0.000176884 | 0.001373882 |
| CC | GO:0005687 | U4 snRNP | 5 | 0.000216811 | 0.001639093 |
| CC | GO:0019774 | proteasome core complex, beta-subunit complex | 5 | 0.000216811 | 0.001639093 |
| CC | GO:0031970 | organelle envelope lumen | 14 | 0.000226949 | 0.001693159 |
| CC | GO:0060205 | cytoplasmic vesicle lumen | 36 | 0.000285778 | 0.002104363 |
| CC | GO:0005689 | U12-type spliceosomal complex | 7 | 0.000300401 | 0.002171073 |
| CC | GO:0031983 | vesicle lumen | 36 | 0.000302495 | 0.002171073 |
| CC | GO:0034399 | nuclear periphery | 18 | 0.000321842 | 0.002281056 |
| CC | GO:0101002 | ficolin-1-rich granule | 23 | 0.000416802 | 0.002917614 |
| CC | GO:0034774 | secretory granule lumen | 34 | 0.000460433 | 0.003183727 |
| CC | GO:0031519 | PcG protein complex | 9 | 0.000536823 | 0.003667209 |
| CC | GO:0035861 | site of double-strand break | 10 | 0.000567318 | 0.0038294 |
| CC | GO:0031968 | organelle outer membrane | 23 | 0.000655529 | 0.004366216 |
| CC | GO:0043596 | nuclear replication fork | 7 | 0.000662248 | 0.004366216 |
| CC | GO:0005741 | mitochondrial outer membrane | 21 | 0.000677897 | 0.004390143 |
| CC | GO:0035578 | azurophil granule lumen | 14 | 0.000681363 | 0.004390143 |
| CC | GO:0019867 | outer membrane | 23 | 0.000757996 | 0.004829028 |
| CC | GO:0000800 | lateral element | 5 | 0.000812362 | 0.005061641 |
| CC | GO:0030677 | ribonuclease P complex | 5 | 0.000812362 | 0.005061641 |
| CC | GO:0005758 | mitochondrial intermembrane space | 12 | 0.000886767 | 0.005465187 |
| CC | GO:0030532 | small nuclear ribonucleoprotein complex | 13 | 0.001236097 | 0.007536202 |
| CC | GO:0071005 | U2-type precatalytic spliceosome | 9 | 0.001480794 | 0.008838 |
| CC | GO:0071011 | precatalytic spliceosome | 9 | 0.001480794 | 0.008838 |
| CC | GO:0097525 | spliceosomal snRNP complex | 12 | 0.001608978 | 0.009503028 |
| CC | GO:0005838 | proteasome regulatory particle | 4 | 0.001737726 | 0.009952432 |
| CC | GO:0030681 | multimeric ribonuclease P complex | 4 | 0.001737726 | 0.009952432 |
| CC | GO:0031080 | nuclear pore outer ring | 4 | 0.001737726 | 0.009952432 |
| CC | GO:0044450 | microtubule organizing center part | 20 | 0.00189747 | 0.010758654 |
| CC | GO:0005652 | nuclear lamina | 4 | 0.002606821 | 0.014350168 |
| CC | GO:0017101 | aminoacyl-tRNA synthetase multienzyme complex | 4 | 0.002606821 | 0.014350168 |
| CC | GO:1990023 | mitotic spindle midzone | 4 | 0.002606821 | 0.014350168 |
| CC | GO:0043601 | nuclear replisome | 5 | 0.002866956 | 0.015630426 |
| CC | GO:1990752 | microtubule end | 6 | 0.003065698 | 0.016554769 |
| CC | GO:0015030 | Cajal body | 11 | 0.003638106 | 0.019460436 |
| CC | GO:0043073 | germ cell nucleus | 5 | 0.003708475 | 0.019651451 |
| CC | GO:0035097 | histone methyltransferase complex | 10 | 0.003837986 | 0.019964567 |
| CC | GO:1902554 | serine/threonine protein kinase complex | 10 | 0.003837986 | 0.019964567 |
| CC | GO:0005685 | U1 snRNP | 6 | 0.004546817 | 0.023225632 |
| CC | GO:0090543 | Flemming body | 6 | 0.004546817 | 0.023225632 |
| CC | GO:0032153 | cell division site | 9 | 0.005001867 | 0.025097865 |
| CC | GO:0032155 | cell division site part | 9 | 0.005001867 | 0.025097865 |
| CC | GO:0005814 | centriole | 15 | 0.005121021 | 0.02547034 |
| CC | GO:0044452 | nucleolar part | 17 | 0.006241242 | 0.030772039 |
| CC | GO:1904949 | ATPase complex | 12 | 0.006444743 | 0.031484506 |
| CC | GO:0000803 | sex chromosome | 6 | 0.006496803 | 0.031484506 |
| CC | GO:0035267 | NuA4 histone acetyltransferase complex | 4 | 0.006883355 | 0.032797162 |
| CC | GO:0043189 | H4/H2A histone acetyltransferase complex | 4 | 0.006883355 | 0.032797162 |
| CC | GO:0001673 | male germ cell nucleus | 4 | 0.008964189 | 0.042355794 |
| CC | GO:0015934 | large ribosomal subunit | 13 | 0.009161737 | 0.042931447 |
| CC | GO:0016607 | nuclear speck | 34 | 0.009265536 | 0.043061956 |
| CC | GO:0009295 | nucleoid | 7 | 0.00948748 | 0.043382266 |
| CC | GO:0042645 | mitochondrial nucleoid | 7 | 0.00948748 | 0.043382266 |
| CC | GO:0000151 | ubiquitin ligase complex | 22 | 0.010296032 | 0.046702799 |
| CC | GO:0044391 | ribosomal subunit | 18 | 0.011035994 | 0.049661973 |
| MF | GO:0016887 | ATPase activity | 54 | 4.28E-14 | 1.65E-11 |
| MF | GO:0008094 | DNA-dependent ATPase activity | 26 | 4.45E-14 | 1.65E-11 |
| MF | GO:0140097 | catalytic activity, acting on DNA | 36 | 1.51E-12 | 3.74E-10 |
| MF | GO:0043142 | single-stranded DNA-dependent ATPase activity | 10 | 4.44E-11 | 8.25E-09 |
| MF | GO:0042623 | ATPase activity, coupled | 41 | 1.15E-10 | 1.71E-08 |
| MF | GO:0003684 | damaged DNA binding | 19 | 1.55E-09 | 1.93E-07 |
| MF | GO:0003697 | single-stranded DNA binding | 23 | 3.37E-09 | 3.58E-07 |
| MF | GO:0004386 | helicase activity | 26 | 5.74E-08 | 5.34E-06 |
| MF | GO:0051082 | unfolded protein binding | 20 | 1.02E-07 | 8.45E-06 |
| MF | GO:0003682 | chromatin binding | 56 | 1.45E-07 | 1.08E-05 |
| MF | GO:0000217 | DNA secondary structure binding | 10 | 4.11E-07 | 2.78E-05 |
| MF | GO:0008017 | microtubule binding | 26 | 1.01E-05 | 0.000624352 |
| MF | GO:0015631 | tubulin binding | 34 | 1.36E-05 | 0.000781028 |
| MF | GO:0004298 | threonine-type endopeptidase activity | 8 | 1.99E-05 | 0.000941103 |
| MF | GO:0070003 | threonine-type peptidase activity | 8 | 1.99E-05 | 0.000941103 |
| MF | GO:0016879 | ligase activity, forming carbon-nitrogen bonds | 11 | 2.02E-05 | 0.000941103 |
| MF | GO:0000400 | four-way junction DNA binding | 7 | 2.30E-05 | 0.001007451 |
| MF | GO:0003678 | DNA helicase activity | 11 | 3.35E-05 | 0.001382613 |
| MF | GO:0016874 | ligase activity | 21 | 6.92E-05 | 0.002686354 |
| MF | GO:0004003 | ATP-dependent DNA helicase activity | 9 | 7.22E-05 | 0.002686354 |
| MF | GO:0000287 | magnesium ion binding | 28 | 8.66E-05 | 0.003067499 |
| MF | GO:0016893 | endonuclease activity, active with either ribo- or deoxyribonucleic acids and producing 5'-phosphomonoesters | 10 | 9.25E-05 | 0.003129333 |
| MF | GO:0035173 | histone kinase activity | 6 | 0.000105159 | 0.003401654 |
| MF | GO:0030983 | mismatched DNA binding | 5 | 0.000170324 | 0.005280034 |
| MF | GO:0008026 | ATP-dependent helicase activity | 11 | 0.000267942 | 0.007667253 |
| MF | GO:0070035 | purine NTP-dependent helicase activity | 11 | 0.000267942 | 0.007667253 |
| MF | GO:0043021 | ribonucleoprotein complex binding | 17 | 0.000279296 | 0.007696169 |
| MF | GO:0070182 | DNA polymerase binding | 6 | 0.00036928 | 0.009812289 |
| MF | GO:0016891 | endoribonuclease activity, producing 5'-phosphomonoesters | 8 | 0.00044472 | 0.011409369 |
| MF | GO:0140098 | catalytic activity, acting on RNA | 31 | 0.000543347 | 0.013470882 |
| MF | GO:0034061 | DNA polymerase activity | 8 | 0.000561287 | 0.013470882 |
| MF | GO:0004540 | ribonuclease activity | 15 | 0.00059633 | 0.013864682 |
| MF | GO:0004518 | nuclease activity | 22 | 0.000830481 | 0.018723565 |
| MF | GO:0008266 | poly(U) RNA binding | 6 | 0.000986076 | 0.021577658 |
| MF | GO:0004521 | endoribonuclease activity | 10 | 0.001111561 | 0.022972253 |
| MF | GO:0042054 | histone methyltransferase activity | 10 | 0.001111561 | 0.022972253 |
| MF | GO:0016853 | isomerase activity | 17 | 0.001298587 | 0.024976085 |
| MF | GO:0003887 | DNA-directed DNA polymerase activity | 6 | 0.00130923 | 0.024976085 |
| MF | GO:0070717 | poly-purine tract binding | 6 | 0.00130923 | 0.024976085 |
| MF | GO:0008187 | poly-pyrimidine tract binding | 6 | 0.001707369 | 0.031757054 |
| MF | GO:0140142 | nucleocytoplasmic carrier activity | 6 | 0.002191018 | 0.037617767 |
| MF | GO:0004523 | RNA-DNA hybrid ribonuclease activity | 4 | 0.002224707 | 0.037617767 |
| MF | GO:0033204 | ribonuclease P RNA binding | 4 | 0.002224707 | 0.037617767 |
| MF | GO:0046974 | histone methyltransferase activity (H3-K9 specific) | 4 | 0.002224707 | 0.037617767 |
| MF | GO:0042393 | histone binding | 21 | 0.002406414 | 0.039661712 |
| MF | GO:0004527 | exonuclease activity | 11 | 0.002452203 | 0.039661712 |
| MF | GO:0140101 | catalytic activity, acting on a tRNA | 14 | 0.002919633 | 0.04621717 |
| MF | GO:0016741 | transferase activity, transferring one-carbon groups | 23 | 0.003069178 | 0.047572259 |
| MF | GO:0001094 | TFIID-class transcription factor complex binding | 4 | 0.00332658 | 0.049499508 |
| MF | GO:0032404 | mismatch repair complex binding | 4 | 0.00332658 | 0.049499508 |

Note: BP, biological processes; CC, cellular component; MF, molecular function.
