# Supplementary material for: Evaluation of clinical value and potential mechanism of MTFR2 in lung adenocarcinoma via bioinformatics
Source: BMC Cancer. 2021 May 26;21:619. doi: 10.1186/s12885-021-08378-3 (PMC8157440; doi:10.1186/s12885-021-08378-3)

**Figure S. PPI network displayed the relationship among MTFR2 coexpressed genes.** (A) PPI network; (B) Relationship networe of Hub genes.

Note: red represents positive related genes, blue represents negative related genes, and other colors represent other Hub genes.


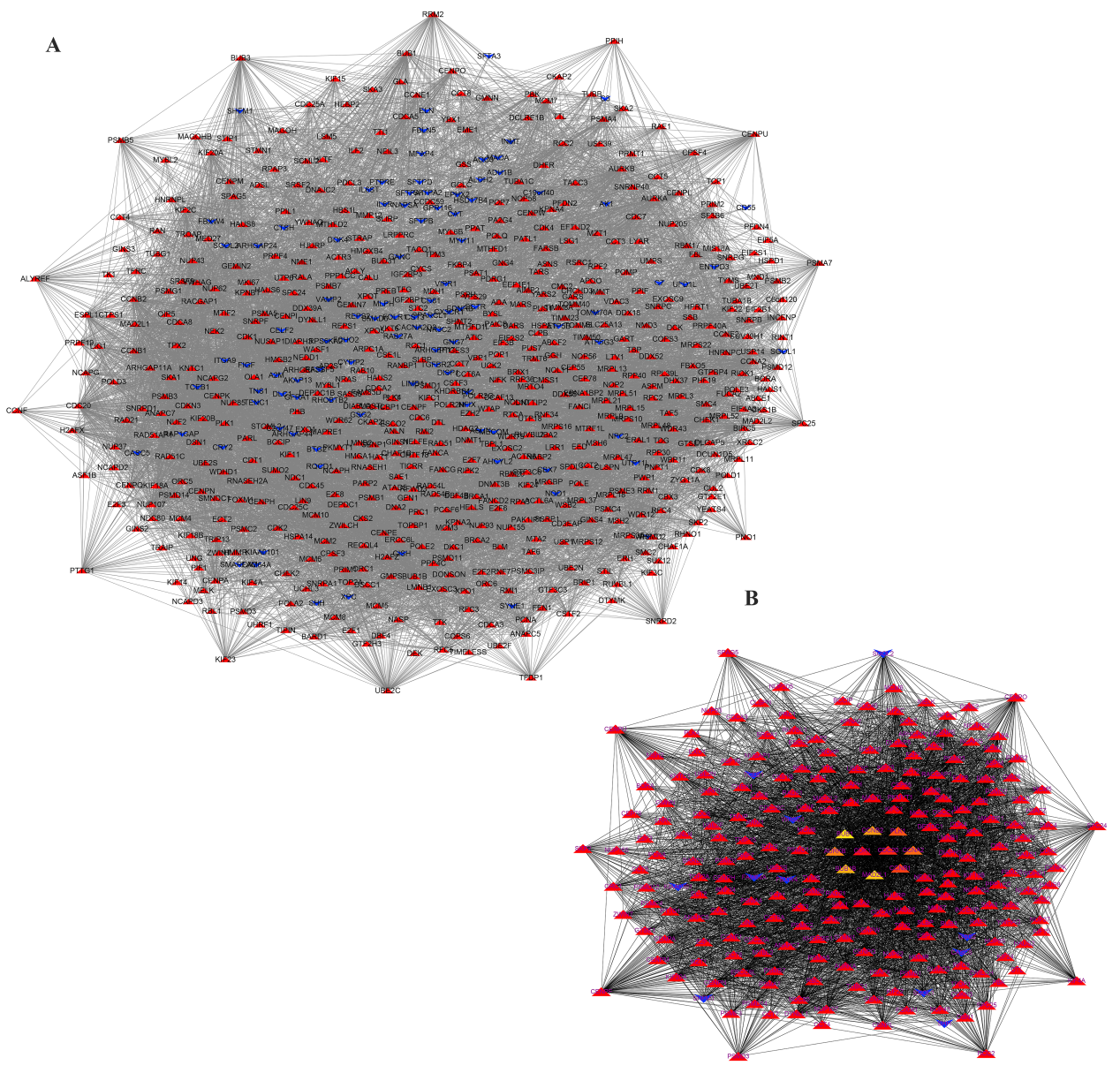

Supplement: Supplementary file 3 — Additional file 3: Figure S1. PPI network displayed the relationship among MTFR2 coexpressed genes. [file 12885_2021_8378_MOESM3_ESM.docx]
